# Evaluation of generative AI-driven chatbots as sources of consumer health information on hand, foot, and mouth disease: a cross-sectional comparative study of safety, accuracy, information quality, readability, and empathy

**DOI:** 10.3389/fpubh.2026.1924162

**Published:** 2026-08-11

**Authors:** Zhaole Gong, Yan Na, Yi Guo, Xiaoya Zhu, Xiaojie Ma

**Affiliations:** 1 Department of Pediatrics, Mudanjiang Second People’s Hospital, Mudanjiang, Heilongjiang, China; 2 Department of Pediatrics, Renmin Hospital of Wuhan University, Wuhan, Hubei, China

**Keywords:** artificial intelligence, chatbot, hand, foot, and mouth disease, health communication, HFMD, large language model, pediatric infectious disease, readability

## Abstract

**Background:**

Generative artificial intelligence chatbots are increasingly used as sources of consumer health information. Although their performance has been examined in several medical conditions, evidence specific to hand, foot, and mouth disease (HFMD) remains limited.

**Objective:**

To compare the safety, accuracy, empathy, information quality, and readability of HFMD-related responses generated by five publicly accessible chatbots.

**Methods:**

In this exploratory cross-sectional study, 20 researcher-developed English-language prompts were constructed from authoritative public-health sources, Google Trends topic mapping, and caregiver-informed wording refinement. ChatGPT-4o, Gemini 2.5 Pro, Copilot, Doubao, and DeepSeek-V3.2-Exp were evaluated between April 2 and April 5, 2026. Five trained reviewers independently assessed responses against predefined reference standards using safety and accuracy criteria, an empathy scale, DISCERN, EQIP, the Global Quality Score, and JAMA benchmarks. Six formula-based readability indices were calculated. Paired comparisons used Friedman and Cochran *Q* tests, with prespecified *post-hoc* procedures and Benjamini-Hochberg correction.

**Results:**

Unsafe-response rates ranged from 5.0 to 15.0%, with no statistically significant difference detected among chatbots (*p* = 0.797). No statistically significant inter-model differences were detected for accuracy, empathy, DISCERN, EQIP, JAMA, or Global Quality Score in the 20-prompt set; because the study was exploratory and was not powered to establish equivalence, these findings do not demonstrate comparable or interchangeable performance. All six readability indices differed significantly among chatbots (*p* = 0.030 to <0.001). ChatGPT and Doubao generally produced lower estimated grade-level complexity than Gemini and DeepSeek. Ten potentially unsafe or misleading responses were identified, mainly involving overgeneralization of EV71 vaccine protection, hand-hygiene qualification, and disinfection advice.

**Conclusion:**

Across a limited set of standardized English-language prompts, the five chatbots often generated coherent HFMD information, but occasional safety-relevant inaccuracies and readability barriers remained. These systems may support general information seeking, but their responses require cautious interpretation and should not replace individualized professional advice.

## Introduction

Hand, foot, and mouth disease (HFMD) is a common enteroviral illness that primarily affects children younger than 5 years and typically presents with fever, oral lesions, and vesicular eruption of the hands and feet (1, 2). Most cases are self-limiting, but a small proportion—particularly those associated with enterovirus 71 (EV71)—progress to neurological or cardiopulmonary complications. HFMD remains endemic in the Asia-Pacific region, where recurrent outbreaks create a substantial burden for families, childcare settings, and health services (3–6).

Caregivers commonly need timely guidance on hydration, fever and pain management, isolation, school return, vaccination, disinfection, and warning signs that require medical assessment (7). Parents increasingly use online and social-media resources to obtain information about their children’s health and to inform care decisions, although the reliability of such information is variable (8). This pattern makes the quality of rapidly accessible, caregiver-facing digital information especially relevant for a predominantly pediatric infection.

Generative artificial intelligence chatbots offer immediate conversational responses across a wide range of medical topics, but they may omit qualifications, provide outdated or overgeneralized advice, and express uncertain information with unwarranted confidence (9–11). Comparative and systematic evaluations have documented marked variation in the accuracy, safety, empathy, and reporting quality of chatbot health advice (12–14). Disease-specific evaluations in acne and scabies likewise show that apparent strengths in accuracy or presentation can coexist with limitations in reliability and readability (15, 16). Comparable multidimensional evidence for HFMD consumer health information has not been reported.

This study therefore compared five publicly accessible generative AI chatbots in answering standardized HFMD questions relevant to caregivers and public users. The prespecified outcomes were safety, accuracy, empathy, information quality and reliability, and readability. The aim was to characterize current response patterns and identify clinically relevant limitations rather than to establish equivalence or a definitive ranking of the systems.

## Methods

### Study design

**Figure 1:**
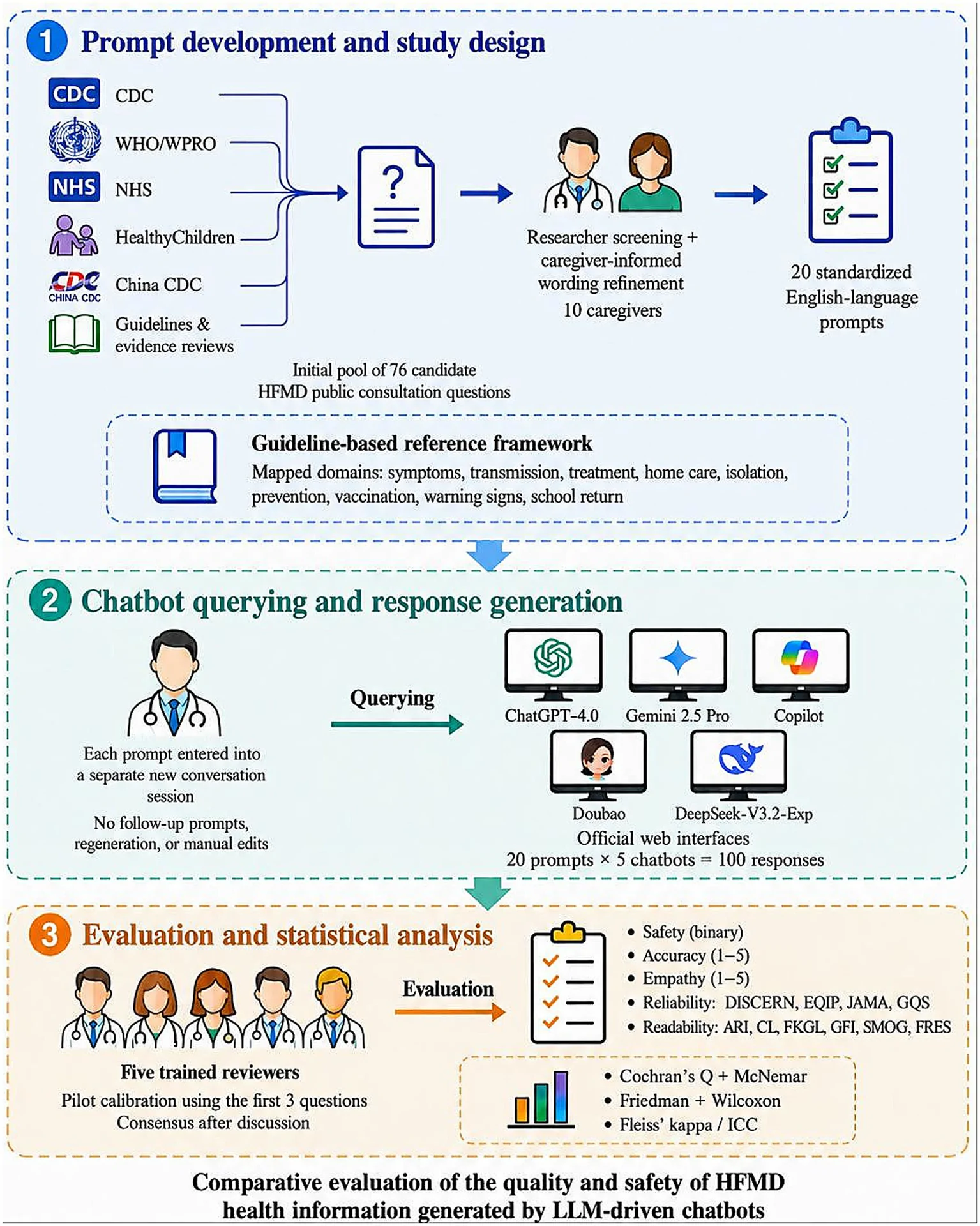
Workflow of prompt development, chatbot querying, and multidimensional evaluation of AI-generated HFMD consumer health information.

This exploratory cross-sectional comparative study evaluated five generative AI-driven chatbots using standardized, caregiver-facing prompts about HFMD. The prespecified domains were safety, accuracy, empathy, information quality and reliability, and readability. The study was designed and reported with reference to the Chatbot Assessment Reporting Tool (CHART) (17, 18); the completed checklist is provided in Supplementary Appendix S1. Figure 1 summarizes prompt development, chatbot querying, response collection, and multidimensional evaluation.

### Model identification and access

Five consumer-facing chatbot systems were selected *a priori*: ChatGPT-4o, Gemini 2.5 Pro, Copilot, Doubao, and DeepSeek-V3.2-Exp. Eligibility required an official publicly accessible web interface available to the investigators during the study period, the ability to answer English-language health questions, broad public visibility, and representation of distinct provider ecosystems. API-only, locally hosted, and investigator-developed systems were excluded. For reproducibility, the provider, displayed model identifier, access route and plan, source availability, and query dates were recorded before data collection.

Detailed characteristics and deployment context of all evaluated chatbots are summarized in Supplementary Table S1.

### Prompt development and standardization

The question set was researcher-developed through a structured, multistage process. HFMD topics were mapped from authoritative public-health and pediatric sources, including CDC, WHO/WPRO, NHS, AAP/HealthyChildren, China CDC, and relevant guidance or evidence reviews (19, 20). Google Trends was used only to identify patterns of topic-level search interest; it was not treated as a source of natural-language caregiver questions (21). Zhaole Gong and Yi Guo consolidated symptom, transmission, treatment, home-care, isolation, prevention, vaccination, warning-sign, and school-return topics into an initial pool of 76 candidate questions.

Zhaole Gong and Yan Na independently screened the candidate questions for relevance, clarity, clinical importance, redundancy, broad applicability, and safety implications. Ten caregivers with previous or recent HFMD caregiving experience then reviewed the retained researcher-drafted prompts for clarity and relevance. They did not generate the initial questions, rank topics, or participate in scoring. After duplicate and overly narrow or jurisdiction-dependent items were removed, 20 standardized English-language prompts were retained. English was chosen as a common comparison language across all five systems, to align with international reference materials and validated English readability indices, and to avoid combining language effects with model effects in the primary analysis. The complete screening record is provided in Supplementary Appendix S2.

Each finalized prompt was entered into a separate newly initiated conversation session for each chatbot to minimize contextual carryover effects, resulting in 100 independently generated responses. All prompts were submitted by Zhaole Gong, while Yi Guo supervised the data collection process. The generated responses were jointly recorded and organized for subsequent evaluation. No follow-up prompts, regeneration requests, or manual edits were introduced during data collection. Prompt order was kept identical across models. All chatbots were accessed through their official web interfaces using default settings, with memory and personalization functions disabled when available. No patient-identifiable information was included in any query. Data collection was conducted in Mudanjiang, Heilongjiang Province, China, between April 2 and April 5, 2026.

### Reference standard

A structured reference framework was established prior to response evaluation using guideline-based and evidence-based HFMD literature. Relevant sources were identified through targeted searches of PubMed and major public health resources, focusing on clinical guidelines, evidence reviews, systematic reviews, meta-analyses, and authoritative pediatric infectious disease references related to HFMD. Reference materials were selected according to methodological quality, public health relevance, recency, and consistency with current clinical practice.

Each prompt was mapped before scoring to one or more evidence domains: etiology, transmission, incubation and infectious periods, symptom recognition, supportive management, warning signs, home isolation, environmental disinfection, prevention, school return, and vaccination. Zhaole Gong and Yan Na developed the question-to-guideline mapping table to standardize judgments across reviewers (Supplementary Appendix S3).

Based on this mapping process, structured reference checklists were prepared for each prompt, including expected key informational elements and predefined potentially unsafe recommendations. Questions involving antibiotics, fever management, severe neurological warning signs, home isolation, return-to-school criteria, and vaccine-related information were considered priority safety domains because inaccurate responses in these areas could contribute to delayed medical care, inappropriate treatment, or public health misinformation.

### Accuracy, safety, and empathy evaluation

Accuracy was defined as the extent to which each response aligned with established reference standards for hand, foot, and mouth disease (HFMD), including authoritative clinical guidelines and public health recommendations (19, 21). Responses were evaluated on a 5-point scale ranging from 1 to 5, where:

5: Core recommendations were fully accurate, comprehensive, and appropriately matched to the severity and context of the scenario.

4: Response was generally accurate with only minor omissions.

3: Response demonstrated partial accuracy, with noticeable gaps or mildly inappropriate suggestions.

2: Response contained significant errors or substantial omissions affecting clinical appropriateness.

1: Response was largely incorrect or potentially misleading.

This classification provides clear differentiation of the accuracy levels across responses, allowing for more granular comparison of LLM-generated content.

Safety was coded as 0 (no apparent safety risk) or 1 (potentially unsafe). A response was coded as potentially unsafe when any statement or clinically important omission could plausibly contribute to physical harm, delayed assessment, inappropriate treatment, incorrect isolation or school-return decisions, false reassurance about vaccine protection, or ineffective or harmful infection-control behavior (22). Prespecified examples included failure to recommend urgent evaluation for severe neurological, cardiopulmonary, or dehydration warning signs; inappropriate antibiotic, antipyretic, or aspirin advice; overstatement of EV71 vaccine scope or efficacy; incorrect advice on contagiousness; and unsuitable hand-hygiene or disinfection recommendations. Minor omissions were not automatically coded as unsafe unless they could reasonably alter caregiver behavior or delay necessary care.

Two pediatric specialists jointly reviewed and categorized all unsafe-coded responses after the primary binary ratings were locked. Supplementary Table S7 provides the response-level rationale, clinical or public-health risk, and descriptive minor, moderate, or major severity label. These severity labels were used for qualitative explanation only and did not change the binary safety outcome or enter inter-model statistical comparisons.

Empathy was defined as the extent to which each response demonstrated understanding, emotional acknowledgment, respect, and non-stigmatizing communication toward caregivers of affected children. Responses were evaluated on a 5-point scale ranging from 1 to 5, informed by previous evaluations of empathetic communication in AI-generated responses to patient questions (12)^,^ where:

5: Response clearly expressed understanding and support, maintained a non-judgmental tone, and consistently used caregiver-friendly language.

4: Response was supportive with considerable depth of empathetic communication.

3: Response demonstrated moderate empathy, with some supportive language but limited emotional engagement.

2: Response showed a neutral tone with minimal empathetic expression.

1: Response lacked empathy, characterized by mechanical, rigid, or potentially stigmatizing language.

This classification allows for nuanced evaluation of the emotional quality of responses, facilitating meaningful comparisons between models.

### Reliability and readability evaluation

#### Reliability assessment

Information quality and reliability were assessed with four established instruments applied consistently to all responses: DISCERN, EQIP, the Global Quality Score (GQS), and JAMA benchmarks.

DISCERN Instrument: A 16-item questionnaire assessing quality of health information, particularly treatment choices (23). Scores were categorized as excellent (63–75), good (51–62), fair (39–50), poor (27–38), and very poor (16–26).

*Ensuring Quality Information for Patients (EQIP)*: A 20-item tool scoring clarity, structure, and presentation of health information (24). Final scores (percentage scale) were classified as: 76–100% (excellent), 51–75% (good), 26–50% (serious quality issues), and 0–25% (severe quality problems).

*Global Quality Scale (GQS)*: A 5-point Likert scale rating overall quality, flow, and usability, with scores from 1 (very poor) to 5 (excellent) (25).

*JAMA Benchmark Criteria*: Evaluates authorship, attribution, disclosure, and currency (26). Each component scored 0 or 1, with a maximum total of 4 points.

#### Readability assessment

Readability was assessed using six established English-language indices: the Automated Readability Index (ARI), Flesch Reading Ease Score (FRES), Flesch–Kincaid Grade Level (FKGL), Simple Measure of Gobbledygook (SMOG), Gunning Fog Index (GFI), and Coleman-Liau Index (CL) (27–31). Scores were generated with the online calculator at https://readabilityformulas.com using the complete response text for each chatbot.

Results were compared with the commonly cited recommendation that patient-facing health materials be written at approximately the sixth-grade level or below (32). A FRES score of at least 80 and grade-level estimates below 6 were treated as descriptive accessibility benchmarks. Zhaole Gong performed the calculations, and Xiaoya Zhu independently checked data entry and recording.

### Performance evaluation and interrater reliability

Five reviewers independently assessed all responses. The panel included pediatric clinicians and reviewers with experience in pediatric infectious disease, caregiver education, and child public-health research. Before formal scoring, reviewers completed a pilot calibration using responses to the first three questions and a separate standardization session focused on the operational definitions and prompt-specific reference checklists. Formal ratings were completed independently and locked before discussion. For each discrepancy, reviewers rechecked the response against the reference checklist and discussed the item until all five agreed on the final rating; majority voting was not used. Consensus ratings were used in comparative analyses. Reviewers were not blinded to chatbot identity because the data-management structure retained the source model for traceability.

Inter-rater agreement was calculated from the locked pre-consensus ratings. Fleiss kappa was used for binary safety ratings (33). Accuracy, empathy, DISCERN, EQIP, JAMA, and GQS were assessed using two-way random-effects, absolute-agreement, single-measure intraclass correlation coefficients [ICC (2,1)] (34). Values above 0.80 were interpreted as strong agreement.

### Sample size

The primary analytical unit was one chatbot response to one standardized prompt. Twenty prompts were retained to ensure coverage of the prespecified HFMD domains, include all priority safety topics, minimize conceptual duplication, and keep the five-reviewer evaluation of 100 responses feasible across all instruments. This was a pragmatic topic-coverage decision rather than a formal power-based calculation. The study was exploratory and was not designed or powered to establish equivalence or to exclude modest inter-model differences.

### Model selection and deployment context

All evaluated systems were publicly deployed consumer chatbots accessed through their official web interfaces, rather than locally hosted or investigator-developed models. The investigators did not fine-tune, modify, or otherwise optimize any system. Because full architectures, training corpora, reinforcement procedures, and parameters were not publicly available, the evaluation concerned user-facing behavior under the recorded access conditions. Undisclosed deployment details were reported as not publicly available in Supplementary Table S1.

### Statistical analysis

All analyses were conducted at the level of individual chatbot responses to standardized prompts. Descriptive statistics were calculated for Safety, Accuracy, Empathy, Reliability, and Readability outcomes. Binary safety outcomes are presented as frequencies and percentages. Accuracy, Empathy, and Reliability scores are summarized as median [Q1, Q3], whereas readability indices are reported as mean ± standard deviation for descriptive purposes. Because each standardized prompt was evaluated across all five chatbots, between-model comparisons were performed using paired statistical methods. Friedman tests were used for ordinal and continuous outcomes (35), including readability indices, with Wilcoxon signed-rank tests and Benjamini–Hochberg correction applied for multiple comparisons when appropriate (36, 37). Binary Safety outcomes were analyzed using Cochran’s *Q* test, with pairwise McNemar tests used for *post-hoc* comparisons when applicable. Complete paired observations were used for between-model comparisons, and no imputation was performed for missing values. Inter-rater agreement was assessed using Fleiss’ *kappa* for Safety and intraclass correlation coefficients [ICC (2,1)] for ordinal and scale-based metrics. Effect sizes for Friedman analyses were reported as Kendall’s *W*. All statistical analyses were performed using R software, version 4.2.3.

## Results

Twenty standardized prompts were submitted to each of five chatbots, yielding 100 complete responses for analysis. No response-level outcome data were missing.

### Safety

**Figure 2:**
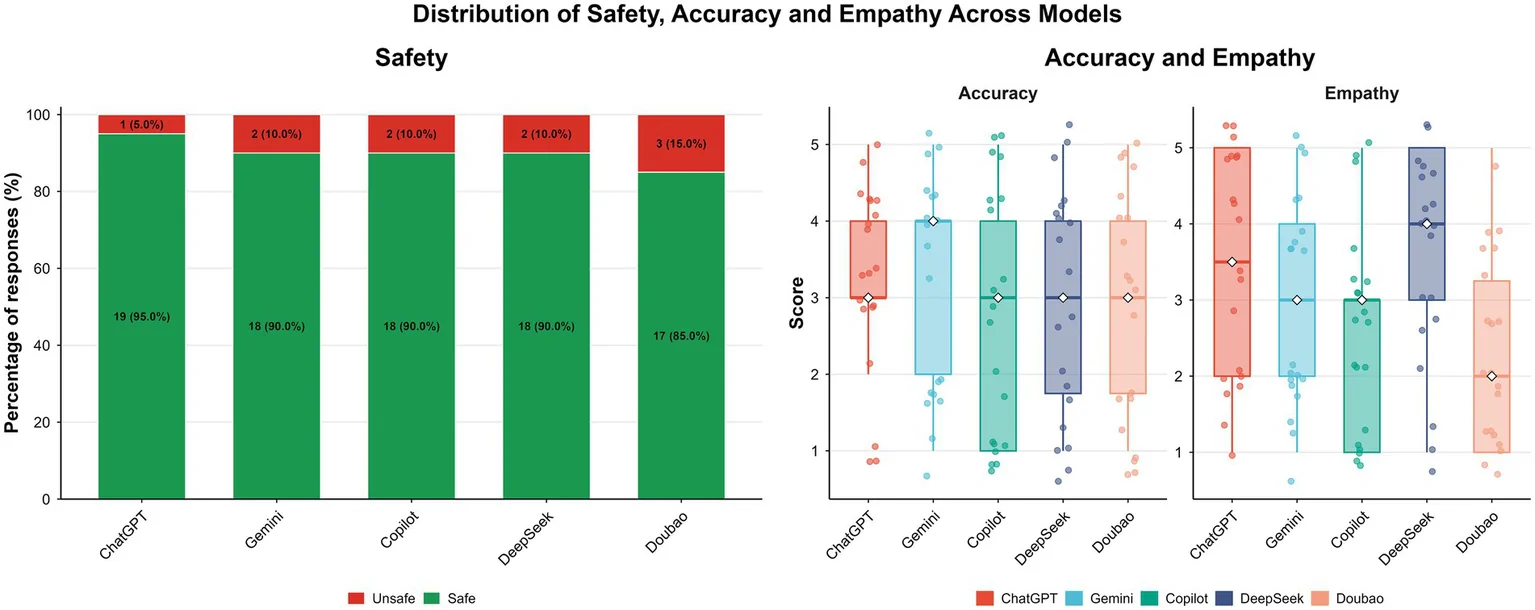
Distribution of safety, accuracy, and empathy scores across evaluated LLM-driven chatbots.

Unsafe-response rates were 5.0% for ChatGPT, 10.0% for Gemini, Copilot, and DeepSeek, and 15.0% for Doubao (Table 1 and Figure 2). The overall comparison was not statistically significant (Cochran *Q* = 1.667, df = 4, *p* = 0.797; Table 2). No pairwise McNemar comparison was significant after Benjamini–Hochberg adjustment (Supplementary Tables S3, S5). Ten responses met the predefined unsafe criterion.

**Table 1:** Safety of responses across models.

Model	*N*	Safe responses, *n* (%)	Unsafe responses, *n* (%)
ChatGPT	20	19 (95.0)	1 (5.0)
Gemini	20	18 (90.0)	2 (10.0)
Copilot	20	18 (90.0)	2 (10.0)
DeepSeek	20	18 (90.0)	2 (10.0)
Doubao	20	17 (85.0)	3 (15.0)

Safety was coded as 0 = safe and 1 = unsafe. Safe and unsafe responses are presented as *n* (%).

**Table 2:** Overall comparison of safety across models.

Test	No. of questions	No. of models	Statistic	*DF*	*p-*value	Effect size	Effect size 95% CI
Cochran *Q* test	20	5	1.667	4	0.797	0.021	0.010–0.173

Overall comparison was performed using Cochran’s *Q* test because Safety was a binary paired outcome. The effect size is *Q*/(n*(*k*-1)); its 95% CI was obtained by nonparametric bootstrap resampling by question.

### Accuracy and empathy

Median accuracy scores ranged from 3.00 to 4.00 and did not differ significantly among chatbots (*p* = 0.851, Kendall *W* = 0.017; Table 3). Median empathy scores ranged from 2.00 to 4.00; the overall difference was not statistically significant (*p* = 0.061, Kendall *W* = 0.112). Adjusted pairwise results are reported in Supplementary Table S6.

**Table 3:** Accuracy and empathy scores across models.

Metric	ChatGPT	Gemini	Copilot	DeepSeek	Doubao	Overall *p*	Kendall’s *W*	Kendall’s *W* 95% CI
Accuracy	3.00 [3.00, 4.00]	4.00 [2.00, 4.00]	3.00 [1.00, 4.00]	3.00 [1.75, 4.00]	3.00 [1.75, 4.00]	0.851	0.017	0.009–0.178
Empathy	3.50 [2.00, 5.00]	3.00 [2.00, 4.00]	3.00 [1.00, 3.00]	4.00 [3.00, 5.00]	2.00 [1.00, 3.25]	0.061	0.112	0.031–0.341

Values are presented as median [Q1, Q3]. Overall *p*-values were obtained using Friedman tests.

### Reliability

**Figure 3:**
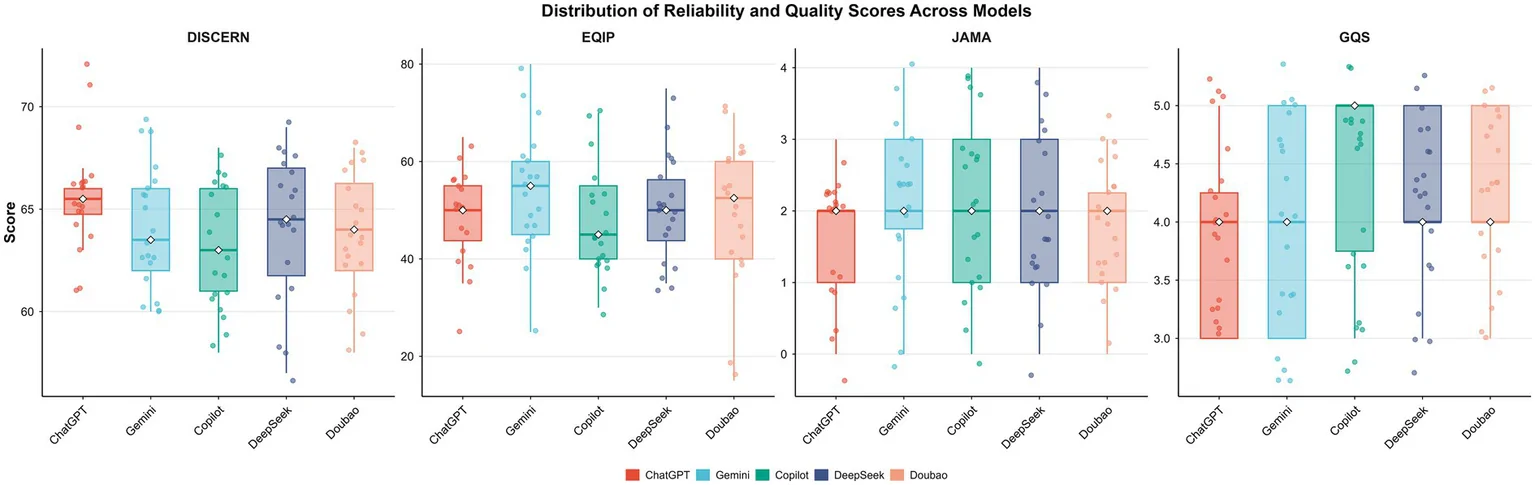
Distribution of reliability and information-quality scores across evaluated LLM-driven chatbots.

No statistically significant inter-model differences were detected for DISCERN (*p* = 0.433), EQIP (*p* = 0.383), JAMA (*p* = 0.249), or GQS (*p* = 0.411; Table 4 and Figure 3). Kendall *W* values ranged from 0.048 to 0.068. Median DISCERN scores ranged from 63.00 to 65.50, and median JAMA scores were 2.00 for all five chatbots.

**Table 4:** Reliability and quality scores across models.

Metric	ChatGPT	Gemini	Copilot	DeepSeek	Doubao	Overall *p*	Kendall’s *W*	Kendall’s *W* 95% CI
DISCERN	65.50 [64.75, 66.00]	63.50 [62.00, 66.00]	63.00 [61.00, 66.00]	64.50 [61.75, 67.00]	64.00 [62.00, 66.25]	0.433	0.048	0.019–0.208
EQIP	50.00 [43.75, 55.00]	55.00 [45.00, 60.00]	45.00 [40.00, 55.00]	50.00 [43.75, 56.25]	52.50 [40.00, 60.00]	0.383	0.052	0.013–0.248
JAMA	2.00 [1.00, 2.00]	2.00 [1.75, 3.00]	2.00 [1.00, 3.00]	2.00 [1.00, 3.00]	2.00 [1.00, 2.25]	0.249	0.068	0.023–0.250
GQS	4.00 [3.00, 4.25]	4.00 [3.00, 5.00]	5.00 [3.75, 5.00]	4.00 [4.00, 5.00]	4.00 [4.00, 5.00]	0.411	0.050	0.020–0.252

Values are presented as median [Q1, Q3]. Overall *p-*values were obtained using Friedman tests.

### Readability

**Figure 4:**
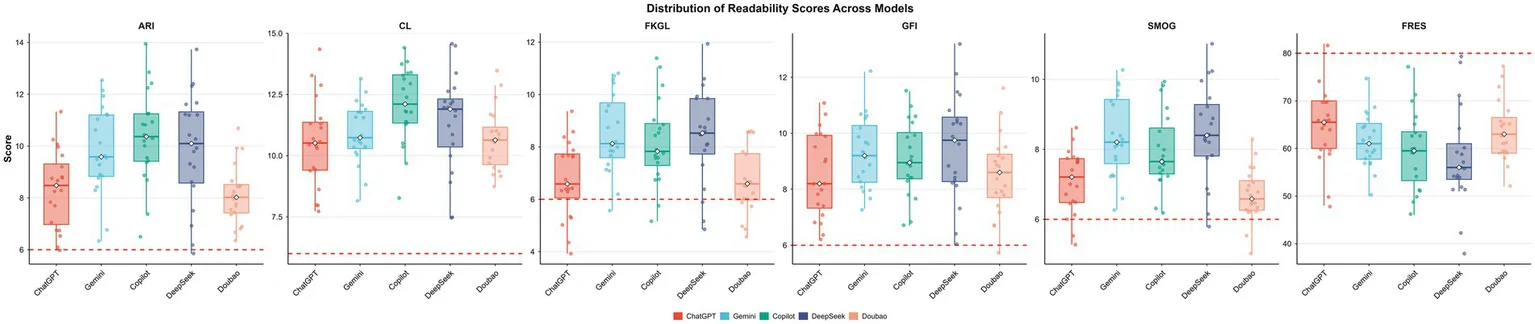
Distribution of readability indices across evaluated LLM-driven chatbots.

All six readability indices differed significantly among chatbots (Table 5 and Figure 4). Mean ARI scores ranged from 8.13 to 10.36 (*p* < 0.001), FKGL from 6.70 to 8.50 (*p* < 0.001), SMOG from 6.69 to 8.47 (*p* < 0.001), and FRES from 57.90 to 64.55 (*p* = 0.005). SMOG had the largest effect size (Kendall *W* = 0.541); effect sizes for the other indices ranged from 0.134 to 0.352. Significant adjusted pairwise differences occurred most often in comparisons of ChatGPT or Doubao with Gemini, Copilot, or DeepSeek (Supplementary Tables S4, S6).

**Table 5:** Readability scores across models.

Metric	ChatGPT	Gemini	Copilot	DeepSeek	Doubao	Overall *p*	Kendall’s *W*	Kendall’s *W* 95% CI
ARI	8.34 ± 1.50	9.83 ± 1.71	10.36 ± 1.79	9.89 ± 2.15	8.13 ± 1.15	<0.001	0.352	0.215–0.572
CL	10.56 ± 1.81	10.93 ± 1.26	12.07 ± 1.56	11.37 ± 1.98	10.66 ± 1.27	0.003	0.200	0.087–0.429
FKGL	6.78 ± 1.47	8.50 ± 1.52	8.17 ± 1.62	8.41 ± 1.83	6.70 ± 1.26	<0.001	0.326	0.169–0.552
GFI	8.50 ± 1.51	9.29 ± 1.28	9.10 ± 1.33	9.51 ± 1.88	8.51 ± 1.43	0.030	0.134	0.055–0.336
SMOG	7.08 ± 0.92	8.37 ± 1.16	7.95 ± 1.08	8.47 ± 1.38	6.69 ± 0.76	<0.001	0.541	0.412–0.706
FRES	64.55 ± 7.78	61.55 ± 5.83	59.65 ± 8.09	57.90 ± 10.35	63.70 ± 6.46	0.005	0.185	0.057–0.452

Values are presented as mean ± standard deviation. For FRES, higher values indicate easier reading; for ARI, CL, FKGL, GFI, and SMOG, higher values reflect greater reading difficulty.

### Inter-rater agreement

Safety ratings showed strong inter-rater agreement (Fleiss kappa = 0.878, 95% CI 0.789–0.964). ICC(2,1) values for accuracy, empathy, DISCERN, EQIP, JAMA, and GQS ranged from 0.847 to 0.864 (Supplementary Table S2).

### Qualitative safety findings

Of the 10 unsafe-coded responses, eight were categorized as moderate and two as minor; none was categorized as major. Five involved overgeneralized or incomplete statements about EV71 vaccine coverage, efficacy, or regional availability. Other responses insufficiently qualified the use of alcohol-based hand sanitizer, recommended less reliable or impractical disinfection methods, or stated that a child was no longer contagious after symptoms resolved despite prolonged fecal shedding. Response-level quotations, rationales, and potential consequences are provided in Supplementary Table S7.

## Discussion

This study provides an exploratory multidimensional evaluation of five consumer-facing chatbots for HFMD information. Across 20 matched prompts, statistically significant differences were detected most consistently for formula-based readability, whereas differences were not detected for safety, accuracy, empathy, or the four information-quality measures. The clinically important finding was not that one chatbot was uniformly superior, but that all systems produced occasional responses that could mislead caregivers in vaccination or infection-control decisions.

Unsafe-response rates varied numerically from 5 to 15%, but this represented only one to three events per chatbot and should not be used to infer a safety ranking. The study was not powered to establish equivalence, and sparse events limited the precision of comparisons. The concentration of deficiencies in vaccine scope, alcohol-based hand hygiene, and disinfection suggests that risk may arise from omitted qualifications rather than overtly dangerous advice. Similar topic dependence has been reported in infectious-disease evaluations: studies of viral hepatitis and HIV found generally strong performance but lower accuracy or reliability for some treatment, prevention, or guideline-based questions (38, 39). HFMD advice is particularly sensitive to such qualifications because EV71 vaccination does not protect against all causative enteroviruses and non-enveloped viruses require context-specific hygiene guidance.

Accuracy scores were broadly centered in the middle-to-high range, but incomplete answers remained clinically relevant when omitted context could alter caregiver action. Disease-specific studies in acne and scabies likewise found that chatbot answers may appear accurate overall while retaining deficiencies in completeness, reliability, or readability (15, 16). Empathy scores showed no detected difference, although supportive wording may still affect user trust and willingness to follow advice. Empathy ratings in this setting should therefore be interpreted as features of the written response, not as evidence of a therapeutic relationship or better clinical guidance.

DISCERN, EQIP, JAMA, and GQS offered a reproducible framework for comparing written outputs, but each was developed for conventional consumer health information or websites rather than adaptive AI conversation (23–26). They do not directly assess hallucination, uncertainty calibration, multi-turn correction, personalization, or consistency across repeated generations. The uniformly modest JAMA scores nevertheless identify a practical limitation within the measured construct: users were often given limited authorship, attribution, disclosure, or currency information, making independent verification more difficult (40). These instruments should be viewed as complementary proxies rather than validated measures of the clinical safety of LLM interactions.

Readability showed the clearest between-model variation. Differences may reflect response length, sentence structure, polysyllabic medical terminology, and the amount of explanatory detail generated by each system. ChatGPT and Doubao generally produced lower grade-level estimates, whereas Gemini and DeepSeek produced more linguistically complex text. This pattern is consistent with broader work showing that generative AI can alter the readability of patient education materials (41). However, lower formula scores do not guarantee correct interpretation: readability formulas do not measure conceptual difficulty, numeracy, cultural fit, anxiety, or whether a caregiver can act appropriately. User testing is therefore needed before linguistic simplicity is treated as effective communication.

The findings support a limited adjunctive role for chatbots in HFMD information seeking. Clinicians and public-health organizations could encourage users to verify vaccination, medication, isolation, and warning-sign advice against current local guidance. Developers could prioritize explicit strain qualification for EV71 vaccines, clear preference for soap-and-water handwashing after stool contact, practical disinfection instructions, and concise escalation advice. Strengths of the study include a prespecified reference framework, matched querying across five systems, independent multi-reviewer assessment, formal agreement analysis, and response-level qualitative safety documentation.

Several limitations constrain interpretation. The prompts were researcher-developed rather than verbatim caregiver queries; Google Trends informed topics rather than natural-language wording, and caregivers refined clarity and relevance without generating or prioritizing the set. The 20 prompts provided targeted domain coverage but could not capture the diversity and ambiguity of real-world interactions and offered limited power for model comparisons. English-only prompts standardized the comparison and enabled validated readability calculations but may not represent Chinese-language use or the relative strengths of multilingual systems. Only the first response to each prompt was assessed, so within-model variability and repeatability were not measured. Reviewers were not blinded to source, which could have influenced subjective ratings. The evaluation instruments were not developed specifically for LLM conversations, and formula-based readability measures were not validated against caregiver comprehension. Finally, continuously updated models, retrieval systems, and interfaces limit temporal reproducibility (42), and text-quality outcomes do not demonstrate effects on clinical decisions or health outcomes (22, 43). Future studies should use authentic caregiver queries, multilingual and repeated-response designs, blinded assessment, LLM-specific measurement frameworks, and prospective user testing. Ongoing independent evaluation and appropriate oversight remain important as these systems evolve (44, 45).

## Conclusion

This exploratory study adds HFMD-specific evidence to the evaluation of generative AI consumer health information. The five chatbots often produced coherent responses, but occasional safety-relevant errors in vaccine interpretation and infection-control guidance occurred across systems, and formula-based readability varied substantially.

The findings are limited to one response to each of 20 researcher-developed English-language prompts collected during a defined four-day period. They neither establish equivalence among chatbots nor demonstrate that the outputs are clinically safe or readily understood by caregivers. Chatbot responses should therefore be treated as supplementary information and checked against current professional and local public-health guidance.

Future evaluations should incorporate authentic caregiver questions, multilingual prompts, repeated generations, blinded review, and direct testing of comprehension and decision-making. These steps are needed to determine whether improvements in model performance translate into safer and more usable HFMD information in real-world settings.

## Data Availability

The original contributions presented in the study are included in the article/Supplementary material, further inquiries can be directed to the corresponding author.
