# Supplementary material for: Evaluation of generative AI-driven chatbots as sources of consumer health information on hand, foot, and mouth disease: a cross-sectional comparative study of safety, accuracy, information quality, readability, and empathy
Source: Front Public Health. 2026 Aug 11;14:1924162. doi: 10.3389/fpubh.2026.1924162 (PMC13503565; doi:10.3389/fpubh.2026.1924162)
Supplement: Supplementary file 1 [file Supplementary_file_1.docx]

**Supplementary Appendix S1. CHART Checklist**

Completed according to the manuscript: Evaluation of Generative AI-Driven Chatbots in Providing Health Advice on Hand, Foot, and Mouth Disease: A Cross-Sectional Comparative Study of Safety, Accuracy, Reliability, Readability, and Empathy.

Checklist used: Chatbot Assessment Reporting Tool (CHART). Page numbers and locations refer to the revised manuscript PDF. Supplementary materials include Supplementary Table S1, Supplementary Tables S2-S7, and Supplementary Appendices S2-S4.

| **Heading** | **No** | **CHART checklist item** | **Reported page/location** |
| --- | --- | --- | --- |
| **Title and abstract** |  |  |  |
| Title | 1a | State that the study is assessing one or more generative AI-driven chatbots for clinical evidence or health advice. | Title, p. 1; Abstract, pp. 1-2. The title and abstract state that the study evaluates generative AI-driven chatbots for HFMD health advice. |
| Abstract/summary | 1b | Apply a structured format, if applicable. | Abstract, pp. 1-2. Structured abstract includes Background, Objective, Methods, Results, and Conclusions. |
| **Introduction** |  |  |  |
| Background | 2a | State the scientific background, rationale, and healthcare context for evaluating the generative AI-driven chatbot(s), referencing relevant literature when applicable. | Introduction, pp. 2-3. The manuscript describes HFMD epidemiology, clinical/public-health relevance, caregiver information needs, and rationale for evaluating LLM-generated HFMD guidance. |
|  | 2b | State the aims and research questions including the target audience, intervention, comparator(s), and outcome(s). | Abstract Objective, p. 1; Introduction, p. 3. Target audience: caregivers/public users; intervention/comparators: five chatbot systems; outcomes: safety, accuracy, empathy, reliability/information quality, and readability. |
| **Methods** |  |  |  |
| Model identifiers | 3a | State the name and version identifier(s) of the generative AI model(s) and chatbot(s) under evaluation, as well as their date of release or last update. | Methods: Model identification and access, p. 5; Model characteristics and deployment context, p. 11; Supplementary Table S1. Model names/version identifiers, access route, access plan, query dates, and availability of update details are reported. |
|  | 3b | State whether the generative AI model(s) and chatbot(s) are open-source or closed-source/proprietary. | Methods: Model identification and access, p. 5; Model characteristics and deployment context, p. 11; Supplementary Table S1. Evaluated systems are reported as proprietary deployed web-based chatbot products. |
| Model details | 4a | State whether the generative AI model was a base model or a novel base model, tuned model, or fine-tuned model. | Methods: Model characteristics and deployment context, p. 11. The evaluated systems are described as publicly deployed user-facing chatbot products rather than locally hosted or investigator-developed models. |
|  | 4b | If a base model is used, cite its development in sufficient detail to identify the model. | Partially reported. Methods: Model characteristics and deployment context, p. 11; Supplementary Table S1. Public model identifiers are provided, while complete architectures, training corpora, reinforcement-learning procedures, and parameter specifications were not accessible. |
|  | 4c | If a novel base model, tuned model, or fine-tuned model is used, describe the pre- and/or post-implementation/deployment data and parameters. | Not applicable. Methods: Model characteristics and deployment context, p. 11. The investigators did not develop, fine-tune, modify, or optimize any model. |
| Prompt engineering | 5a | Describe the evolution of study prompt development. | Methods: Prompt development and standardization, pp. 6-7; Figure 1, p. 5; Supplementary Appendix S2. The manuscript reports source mapping, 76-question pool generation, independent review, caregiver-informed wording refinement, and final selection of 20 prompts. |
|  | 5ai | Describe the sources of prompts. | Methods: Prompt development and standardization, p. 6; Supplementary Appendix S2. Sources included CDC, WHO/WPRO, NHS, AAP/HealthyChildren, China CDC, clinical guidelines/evidence reviews, and Google Trends-informed search-interest patterns. |
|  | 5aii | State the number and characteristics of the individual(s) involved in prompt engineering. | Methods: Prompt development and standardization, p. 6. Zhaole Gong and Yi Guo collected/consolidated questions; Zhaole Gong and Yan Na independently reviewed candidate questions; 10 caregivers with previous or recent HFMD caregiving experience reviewed clarity/relevance. |
|  | 5aiii | Provide details of any patient and public involvement during prompt engineering. | Methods: Prompt development and standardization, p. 6; Ethics, p. 19. Caregivers provided non-identifiable feedback on prompt clarity and relevance only and did not participate in scoring or outcome evaluation. |
|  | 5b | Provide study prompts. | Supplementary Appendix S2 and Supplementary Appendix S4; Methods, pp. 6 and 11. The final question set and cleaned chatbot response records are provided in the supplementary materials. |
| Query strategy | 6a | State route of access to generative AI model. | Methods: Model identification and access, p. 5; Prompt development and standardization, pp. 6-7; Supplementary Table S1. Chatbots were accessed through official web interfaces using default settings. |
|  | 6b | State the date(s) and location(s) of queries for the generative AI-driven chatbot(s) including the day, month, and year as well as city and country. | Methods: Prompt development and standardization, p. 7; Supplementary Table S1. Queries were conducted in Mudanjiang, Heilongjiang Province, China, between April 2 and April 5, 2026. |
|  | 6c | Describe whether prompts were input into separate chat session(s). | Methods: Prompt development and standardization, p. 7. Each finalized prompt was entered into a separate newly initiated conversation session for each chatbot to minimize contextual carryover. |
|  | 6d | Provide all generative AI-driven chatbot outputs/responses. | Methods: Model characteristics and deployment context, p. 11; Supplementary Appendix S4. Cleaned response records are provided; no follow-up prompts, regeneration requests, or manual edits were introduced. |
| Performance evaluation | 7a | Define the ground truth or reference standard used to define successful generative AI-driven chatbot performance. | Methods: Reference standard, pp. 7-8; Supplementary Appendix S3. A structured guideline-based reference framework and prompt-specific checklists were prepared, including expected key elements and predefined potentially unsafe recommendations. |
|  | 7b | Describe the process undertaken for generative AI-driven chatbot performance evaluation. | Methods: Accuracy, safety, and empathy evaluation, pp. 7-8; Reliability and Readability evaluation, pp. 8-10; Performance evaluation and interrater reliability, p. 10. The manuscript describes scoring domains, instruments, calibration, independent review, consensus scoring, and inter-rater reliability. |
|  | 7bi | State the number and characteristics of team members involved in performance evaluation. | Methods: Performance evaluation and interrater reliability, p. 10. Five trained reviewers independently assessed all responses; pediatric/public-health expertise involved in prompt and reference development is described in Methods. |
|  | 7bii | Provide details of any patients and public involvement during the evaluation process. | Methods: Prompt development and standardization, p. 6; Ethics, p. 19. Caregivers were involved only in prompt wording refinement; no patients or public participants were involved in formal performance evaluation. |
|  | 7biii | State whether evaluators were blinded to the identity of the generative AI-driven chatbot(s) under assessment. | Methods: Performance evaluation and interrater reliability, p. 10; Discussion limitations, p. 18. Evaluators were not blinded; the manuscript explains traceability reasons and discusses potential assessor expectation bias. |
| Sample size | 8 | Report how the sample size was determined. | Methods: Sample size, p. 11. Sample size was determined pragmatically by topic coverage, high-priority HFMD public health domains, and feasibility of expert evaluation; 20 prompts x 5 chatbots generated 100 responses. |
| Data analysis | 9a | Describe statistical analysis methods, including any evaluation of reproducibility of generative AI-driven chatbot responses. | Methods: Statistical analysis, pp. 11-12. The manuscript describes descriptive statistics, paired tests, post hoc procedures, multiple-comparison correction, effect sizes, complete-pair handling, inter-rater agreement, and R version. Repeated-query reproducibility was not assessed. |
|  | 9ai | Report the measures used for performance evaluation. | Methods: Accuracy, safety, and empathy evaluation, pp. 7-8; Reliability and Readability evaluation, pp. 8-10; Statistical analysis, pp. 11-12. Measures include Safety, Accuracy, Empathy, DISCERN, EQIP, JAMA, GQS, ARI, FRES, FKGL, SMOG, GFI, and CL. |
| **Results** |  |  |  |
|  | 10a | Report the performance evaluation undertaken including the alignment between generative AI-driven chatbot output and ground truth or reference standard using quantitative or mixed methods approaches as applicable. | Results, pp. 12-16; Tables 1-5; Figures 2-4. Quantitative performance evaluation is reported for safety, accuracy, empathy, reliability/information quality, readability, and inter-rater agreement. |
|  | 10b | For responses deviating from the ground truth or reference standard, state the nature of the difference(s). | Results: Safety, p. 12; Discussion, pp. 16-17; Supplementary Table S7. Deviations involved EV71 vaccine interpretation, suboptimal disinfection practices, and incomplete hand hygiene guidance. |
|  | 10c | Report the evaluation for potentially harmful, biased, or misleading responses. | Results: Safety, p. 12; Tables 1-2; Supplementary Table S7. Potentially unsafe or misleading responses were quantified and qualitatively summarized. |
| **Discussion** |  |  |  |
|  | 11a | Interpret study findings in the context of relevant evidence. | Discussion, pp. 16-18. Findings are interpreted in relation to LLM health advice, safety, source transparency, readability, and public health communication literature. |
|  | 11b | Describe the strengths and limitations of the study. | Discussion limitations, pp. 17-18. Limitations include limited English-language prompt set, model/version changes, lack of evaluator blinding, formula-based readability assessment, no user comprehension testing, and subjective judgment. |
|  | 11c | Describe the potential implications for practice, education, policy, regulation, and research. | Discussion, pp. 17-18; Conclusions, pp. 18-19. Implications include plain-language communication, source transparency, context-sensitive safety guidance, human oversight, and continued evaluation across clinical scenarios, languages, and healthcare contexts. |
| **Open science** |  |  |  |
| Disclosures | 12a | Report any relevant conflicts of interest for all authors. | Conflicts of interest, p. 20. Authors declare no competing interests. |
| Funding | 12b | Report sources of funding and their role in the conduct and reporting of the study. | Funding, p. 20. Authors report no specific funding. |
| Ethics | 12c | Describe the process undertaken for ethical approval. | Ethics approval and consent to participate, p. 19. Formal institutional ethics approval and written informed consent were waived according to local institutional policy for minimal-risk, non-identifiable public involvement. |
|  | 12ci | Describe the measures taken to safeguard data privacy of patient health information, as applicable. | Data privacy and confidentiality, p. 19. No patient-identifiable information, personal health records, or protected clinical data were entered into chatbot systems. |
|  | 12cii | State whether permission/licensing was obtained for the use of original, copyrighted data. | Use of copyrighted materials, p. 19. Prompts were investigator-developed using publicly available health-information topics and caregiver-informed refinement; no copyrighted proprietary datasets, patient records, or licensed commercial databases were reproduced. |
| Protocol | 12d | Provide a study protocol. | Protocol registration, p. 20. No formal prospective protocol registration was performed for this exploratory cross-sectional evaluation study. |
| Data availability | 12e | State where study data, code repository, and model parameters can be accessed. | Data availability, p. 20; Model characteristics and deployment context, p. 11. Datasets, prompts, scoring frameworks, pairwise tables, and chatbot responses are available in supplementary materials or from the corresponding author; complete model parameters/architectures were not accessible. |

Footer Note: AI, artificial intelligence; LLM, large language model; HFMD, hand, foot, and mouth disease.
