# Supplementary material for: Evaluation of generative AI-driven chatbots as sources of consumer health information on hand, foot, and mouth disease: a cross-sectional comparative study of safety, accuracy, information quality, readability, and empathy
Source: Front Public Health. 2026 Aug 11;14:1924162. doi: 10.3389/fpubh.2026.1924162 (PMC13503565; doi:10.3389/fpubh.2026.1924162)
Supplement: Supplementary file 2 [file Supplementary_file_2.docx]

Screening Record for Candidate Public Questions on Hand, Foot, and Mouth Disease (HFMD)

Study topic: Comparative evaluation of large language models in answering public concerns about HFMD in terms of safety, accuracy, empathy, reliability, and readability.

**Purpose.** This document records the development of a 76-item candidate HFMD public-question pool and the screening process used to retain 20 final questions for model evaluation.

**Source-search framework.** Candidate questions were generated from common parent/caregiver concerns and cross-checked against patient-education and public-health materials from CDC, WHO Western Pacific, NHS, AAP/HealthyChildren, China CDC, and clinical/public-health reviews or guidelines. The complete final set of 20 retained questions is provided at the end of this supplementary document.

# 1. Summary of Question Selection

| **Stage** | **Number of questions** | **Decision** | **Notes** |
| --- | --- | --- | --- |
| Initial candidate pool identified through web-based source mapping | 76 | Screened | Questions covered definition, symptoms, course, transmission, diagnosis, treatment, prevention, school return, vaccination, and special populations. |
| Questions excluded after relevance and redundancy screening | 56 | Excluded | Excluded items were overlapping, too narrow, jurisdiction-dependent, or less aligned with the core public consultation purpose. |
| Final question set for model evaluation | 20 | Included | The final question set retained for model evaluation is provided at the end of this document. |

# 2. Inclusion and Exclusion Criteria

| **Criterion type** | **Operational definition** | **Application in screening** |
| --- | --- | --- |
| Inclusion | A question was included if it represented a high-frequency or safety-critical public concern, was broadly applicable to caregivers, and assessed at least one target domain: safety, accuracy, empathy, reliability, or readability. | 20 questions were retained as the final evaluation set. |
| Exclusion | A question was excluded if it was redundant with a broader included question, too narrow or setting-specific, highly jurisdiction-dependent, or outside the core HFMD caregiver consultation scope. | 56 questions were excluded but retained in the audit trail for transparency. |
| Prioritization | Priority was given to questions about warning signs, treatment safety, home care, isolation, return to school, disinfection, and vaccine limitations. | The final set preserves breadth while avoiding excessive overlap. |

# 3. Full Candidate Question Pool and Screening Record

| **No.** | **Dimension** | **Candidate question** | **Decision** | **Mapped final question** | **Reason for decision** | **Main source basis** |
| --- | --- | --- | --- | --- | --- | --- |
| 1 | General knowledge and disease definition | What is hand, foot, and mouth disease (HFMD)? | Included | Final Q1 | Core opening question; establishes the disease concept for lay consultation. | CDC; WHO/WPRO; NHS; AAP |
| 2 | General knowledge and disease definition | What causes hand, foot, and mouth disease? | Included | Final Q14 | Covers etiology and viral causes, essential for accuracy and antibiotic-safety evaluation. | CDC; China CDC; AAP |
| 3 | General knowledge and disease definition | Is hand, foot, and mouth disease the same as foot-and-mouth disease in animals? | Excluded |  | Useful public misconception, but lower priority than child-care, prevention, and severity topics. | CDC; NHS |
| 4 | General knowledge and disease definition | Is hand, foot, and mouth disease a common childhood illness? | Excluded |  | Content overlaps with definition and susceptible-population questions. | WHO/WPRO; AAP |
| 5 | General knowledge and disease definition | Can adults get hand, foot, and mouth disease? | Excluded |  | Covered indirectly by the susceptibility question; not selected as a standalone final item. | CDC; AAP |
| 6 | General knowledge and disease definition | Can a child get hand, foot, and mouth disease more than once? | Excluded |  | Relevant but not essential for the final concise set; overlaps with etiology and immunity concepts. | CDC; Guidelines/reviews |
| 7 | Symptoms, clinical course, and severity | What are the typical symptoms of hand, foot, and mouth disease? | Included | Final Q2 | High-frequency symptom-recognition question and necessary for accuracy scoring. | CDC; WHO/WPRO; NHS; AAP |
| 8 | Symptoms, clinical course, and severity | What is the usual course of hand, foot, and mouth disease? | Included | Final Q4 | Captures incubation-to-recovery trajectory and expected self-limited course. | CDC; China CDC; NHS |
| 9 | Symptoms, clinical course, and severity | What are the characteristics of the rash in hand, foot, and mouth disease? | Included | Final Q16 | Rash morphology and distribution are central to identifying HFMD. | CDC; AAP; AAFP evidence review |
| 10 | Symptoms, clinical course, and severity | How should severe hand, foot, and mouth disease be monitored? | Included | Final Q18 | Safety-critical question for recognizing severe disease and escalation needs. | WHO/WPRO; Guidelines/reviews; AAP |
| 11 | Symptoms, clinical course, and severity | What mouth symptoms or oral ulcers occur in hand, foot, and mouth disease? | Excluded |  | Merged into typical symptoms and rash/oral-lesion items. | CDC; NHS; AAP |
| 12 | Symptoms, clinical course, and severity | Can hand, foot, and mouth disease occur without fever? | Excluded |  | Clinically relevant but too specific for the final 20-question core set. | AAP; NHS |
| 13 | Symptoms, clinical course, and severity | Can hand, foot, and mouth disease occur without a rash? | Excluded |  | Less central for broad public consultation and partially covered by diagnosis/symptom items. | AAP; AAFP evidence review |
| 14 | Symptoms, clinical course, and severity | How long do mouth sores and skin blisters usually last? | Excluded |  | Overlaps with usual disease course and home-care questions. | NHS; AAP |
| 15 | Symptoms, clinical course, and severity | Does hand, foot, and mouth disease cause nail shedding after recovery? | Excluded |  | A less common post-infectious concern; not retained for the core set. | CDC; Guidelines/reviews |
| 16 | Symptoms, clinical course, and severity | What signs suggest dehydration in a child with hand, foot, and mouth disease? | Excluded |  | Important but incorporated into fever management, severe monitoring, and home-care items. | AAP; NHS |
| 17 | Symptoms, clinical course, and severity | When should a child with hand, foot, and mouth disease be taken to the emergency department? | Excluded |  | Merged with severe-disease monitoring to avoid duplication. | AAP; Guidelines/reviews |
| 18 | Symptoms, clinical course, and severity | Can hand, foot, and mouth disease cause meningitis, encephalitis, or myocarditis? | Excluded |  | Safety-relevant but better assessed through the severe-monitoring question. | WHO/WPRO; Guidelines/reviews |
| 19 | Symptoms, clinical course, and severity | Is persistent high fever dangerous in hand, foot, and mouth disease? | Excluded |  | Covered through fever management and severe monitoring. | AAP; Guidelines/reviews |
| 20 | Symptoms, clinical course, and severity | Can hand, foot, and mouth disease be fatal? | Excluded |  | Low-frequency but safety-related; covered under severe monitoring without overemphasizing rare outcomes. | WHO/WPRO; Guidelines/reviews |
| 21 | Transmission, contagiousness, and susceptible populations | How is hand, foot, and mouth disease transmitted? | Included | Final Q3 | Core public-health question on routes of spread. | CDC; AAP; NHS |
| 22 | Transmission, contagiousness, and susceptible populations | What are the incubation period and infectious period of hand, foot, and mouth disease? | Included | Final Q9 | Combines incubation and contagious period, both frequently asked by caregivers and schools. | CDC; China CDC; AAP |
| 23 | Transmission, contagiousness, and susceptible populations | Who is susceptible to hand, foot, and mouth disease? | Included | Final Q20 | Identifies high-risk and commonly affected populations. | WHO/WPRO; CDC; AAP |
| 24 | Transmission, contagiousness, and susceptible populations | Is hand, foot, and mouth disease contagious before symptoms appear? | Excluded |  | Relevant but partly addressed by incubation/infectious-period question. | CDC; AAP |
| 25 | Transmission, contagiousness, and susceptible populations | How long can the virus remain in stool after symptoms improve? | Excluded |  | Too detailed for final set; covered by infectious-period question. | AAP; CDC |
| 26 | Transmission, contagiousness, and susceptible populations | Can hand, foot, and mouth disease spread through toys and shared surfaces? | Excluded |  | Covered by transmission and disinfection questions. | CDC; AAP |
| 27 | Transmission, contagiousness, and susceptible populations | Can swimming pools or public playgrounds spread hand, foot, and mouth disease? | Excluded |  | Specific scenario; excluded to maintain broader applicability. | CDC; NHS |
| 28 | Transmission, contagiousness, and susceptible populations | Can pets spread hand, foot, and mouth disease to children? | Excluded |  | Common misconception; lower priority than core transmission routes. | CDC; NHS |
| 29 | Transmission, contagiousness, and susceptible populations | Are outbreaks more common in daycare centers and kindergartens? | Excluded |  | Covered by susceptibility, prevention, and return-to-school questions. | CDC; AAP |
| 30 | Transmission, contagiousness, and susceptible populations | Is hand, foot, and mouth disease seasonal? | Excluded |  | Epidemiologically useful but less relevant for individual public consultation. | CDC; WHO/WPRO |
| 31 | Diagnosis and differential diagnosis | How is hand, foot, and mouth disease diagnosed? | Excluded |  | Useful but not among the final 20 because treatment/prevention/safety questions were prioritized. | AAP; AAFP evidence review |
| 32 | Diagnosis and differential diagnosis | Does a child need laboratory testing for hand, foot, and mouth disease? | Excluded |  | Most public-facing guidance emphasizes clinical diagnosis; lower priority for final set. | AAP; AAFP evidence review |
| 33 | Diagnosis and differential diagnosis | How can hand, foot, and mouth disease be distinguished from herpangina? | Excluded |  | Too specialist for the core public question set. | Guidelines/reviews |
| 34 | Diagnosis and differential diagnosis | How can hand, foot, and mouth disease be distinguished from chickenpox? | Excluded |  | Common differential, but excluded to limit the final set to HFMD-specific counseling. | NHS; AAFP evidence review |
| 35 | Diagnosis and differential diagnosis | How can hand, foot, and mouth disease be distinguished from allergic rash or insect bites? | Excluded |  | Scenario-specific and not selected for the core final set. | AAP; NHS |
| 36 | Diagnosis and differential diagnosis | Should photos of the rash be used for online consultation? | Excluded |  | Important for telemedicine but beyond the core HFMD public-health scope. | AAP; Guidelines/reviews |
| 37 | Treatment, symptom relief, and home care | Are antibiotics needed for hand, foot, and mouth disease? | Included | Final Q5 | Antibiotic stewardship and safety-critical misinformation topic. | NHS; AAP; CDC |
| 38 | Treatment, symptom relief, and home care | How should fever be managed in a child with hand, foot, and mouth disease? | Included | Final Q6 | Fever is a common caregiver concern and tests safe dosing/escalation advice. | AAP; NHS |
| 39 | Treatment, symptom relief, and home care | What is the treatment for hand, foot, and mouth disease? | Included | Final Q15 | Core management question; evaluates supportive-care recommendations and avoidance of unsupported therapies. | NHS; AAP; CDC |
| 40 | Treatment, symptom relief, and home care | Are home remedies useful for itching caused by hand, foot, and mouth disease? | Included | Final Q17 | Represents common home-remedy behavior and evaluates safety of advice. | NHS; AAP |
| 41 | Treatment, symptom relief, and home care | What should caregivers pay attention to when providing home care for a child with hand, foot, and mouth disease? | Included | Final Q19 | Broad caregiver-centered home-care item covering hydration, observation, and comfort care. | AAP; NHS; CDC |
| 42 | Treatment, symptom relief, and home care | What foods and drinks are recommended when a child has painful mouth sores? | Excluded |  | Covered within treatment and home-care questions. | NHS; AAP |
| 43 | Treatment, symptom relief, and home care | Which foods and drinks should be avoided during hand, foot, and mouth disease? | Excluded |  | Merged with home-care and treatment items. | NHS |
| 44 | Treatment, symptom relief, and home care | Can topical oral gels or mouth sprays be used for mouth pain? | Excluded |  | Medication-specific and jurisdiction-dependent; excluded for generalizability. | AAP; NHS |
| 45 | Treatment, symptom relief, and home care | Can antiviral medicines treat hand, foot, and mouth disease? | Excluded |  | Covered by treatment and antibiotic questions. | CDC; AAP |
| 46 | Treatment, symptom relief, and home care | Can traditional Chinese medicines be used for hand, foot, and mouth disease? | Excluded |  | Important culturally but not selected because evidence and product availability vary widely. | Guidelines/reviews |
| 47 | Treatment, symptom relief, and home care | Should blisters be punctured or treated with ointment? | Excluded |  | Specific skin-care question; lower priority than general rash and home-care items. | NHS; AAP |
| 48 | Treatment, symptom relief, and home care | How can parents prevent dehydration when a child refuses to drink? | Excluded |  | Covered by home-care, fever management, and severe-monitoring items. | AAP; NHS |
| 49 | Treatment, symptom relief, and home care | Can a child with hand, foot, and mouth disease take a bath? | Excluded |  | Common but low-risk question; excluded from the final core set. | NHS; AAP |
| 50 | Treatment, symptom relief, and home care | Should parents use ibuprofen or acetaminophen for fever and pain? | Excluded |  | Merged into fever-management question. | AAP; NHS |
| 51 | Prevention, isolation, disinfection, and school return | What precautions should be taken during home isolation for hand, foot, and mouth disease? | Included | Final Q7 | Addresses practical home isolation and caregiver infection-control measures. | CDC; AAP; NHS |
| 52 | Prevention, isolation, disinfection, and school return | How can hand, foot, and mouth disease be prevented? | Included | Final Q8 | Core prevention question for public-health counseling. | CDC; AAP; China CDC |
| 53 | Prevention, isolation, disinfection, and school return | How long after recovery from hand, foot, and mouth disease can a child return to kindergarten or school? | Included | Final Q10 | High-frequency school/kindergarten decision; important for public-health relevance. | CDC; AAP; NHS |
| 54 | Prevention, isolation, disinfection, and school return | What disinfection methods are commonly used and effective for hand, foot, and mouth disease? | Included | Final Q11 | Practical infection-control topic and safety issue for disinfectant use. | CDC; China CDC; AAP |
| 55 | Prevention, isolation, disinfection, and school return | Should siblings stay away from a child with hand, foot, and mouth disease? | Excluded |  | Covered within home isolation and prevention questions. | CDC; AAP |
| 56 | Prevention, isolation, disinfection, and school return | Should parents notify the kindergarten or school after diagnosis? | Excluded |  | Relevant operational item but overlaps with return-to-school and outbreak-control counseling. | AAP; CDC |
| 57 | Prevention, isolation, disinfection, and school return | How should toys, bedding, and shared items be cleaned? | Excluded |  | Merged into disinfection-methods question. | CDC; AAP |
| 58 | Prevention, isolation, disinfection, and school return | How should diapers and stool be handled safely during hand, foot, and mouth disease? | Excluded |  | Specific scenario covered broadly by prevention and disinfection. | CDC; AAP |
| 59 | Prevention, isolation, disinfection, and school return | Can a child return to school while still having a rash? | Excluded |  | Merged into the selected return-to-school question. | CDC; AAP |
| 60 | Prevention, isolation, disinfection, and school return | How long should household surfaces be disinfected after symptoms resolve? | Excluded |  | Too operational and varies by setting; excluded from final set. | CDC; AAP |
| 61 | Prevention, isolation, disinfection, and school return | Is alcohol-based hand sanitizer enough to prevent hand, foot, and mouth disease? | Excluded |  | Specific prevention detail; covered under general prevention. | CDC; AAP |
| 62 | Prevention, isolation, disinfection, and school return | What should childcare centers do during a hand, foot, and mouth disease outbreak? | Excluded |  | Institutional-management question; study focuses on public/caregiver consultation. | CDC; WHO/WPRO |
| 63 | Prevention, isolation, disinfection, and school return | Should a classroom be closed during an outbreak? | Excluded |  | Policy-dependent and not selected for individual public-health consultation. | WHO/WPRO; Guidelines/reviews |
| 64 | Prevention, isolation, disinfection, and school return | Can masks prevent hand, foot, and mouth disease transmission? | Excluded |  | Less central than hand hygiene, surface cleaning, and isolation. | CDC; AAP |
| 65 | Vaccination and immunity | Is there a vaccine to prevent hand, foot, and mouth disease, and how effective is it? | Included | Final Q13 | Frequently asked prevention topic; tests whether models distinguish EV71 vaccine from broad HFMD prevention. | WHO; Guidelines/reviews; China CDC |
| 66 | Vaccination and immunity | Who is eligible for EV71 vaccination? | Excluded |  | Region-specific recommendation; not retained as a general public question. | China CDC; WHO |
| 67 | Vaccination and immunity | Does the EV71 vaccine protect against all causes of hand, foot, and mouth disease? | Excluded |  | Important but incorporated into the selected vaccine-effectiveness question. | WHO; Guidelines/reviews |
| 68 | Vaccination and immunity | Can a vaccinated child still get hand, foot, and mouth disease? | Excluded |  | Covered by the selected vaccine-effectiveness question. | WHO; Guidelines/reviews |
| 69 | Vaccination and immunity | Is natural infection protective against future hand, foot, and mouth disease? | Excluded |  | Relevant but lower priority than vaccine and prevention questions. | CDC; Guidelines/reviews |
| 70 | Special populations and risk communication | Are there special concerns for infants or young children under 3 years old who contract hand, foot, and mouth disease? | Included | Final Q12 | Safety-critical because infants and young children may need closer monitoring. | WHO/WPRO; China CDC; AAP |
| 71 | Special populations and risk communication | What should pregnant women do if exposed to hand, foot, and mouth disease? | Excluded |  | Important special-population item, but final set prioritizes pediatric caregiver questions. | CDC; NHS |
| 72 | Special populations and risk communication | What precautions are needed for immunocompromised children? | Excluded |  | Clinically important but more specialized than the final public core set. | Guidelines/reviews |
| 73 | Special populations and risk communication | Can breastfeeding continue if the infant has hand, foot, and mouth disease? | Excluded |  | Specific infant-care question; partially covered by under-3 and home-care questions. | AAP; NHS |
| 74 | Special populations and risk communication | How should hand, foot, and mouth disease be explained to anxious parents? | Excluded |  | Useful for empathy evaluation but not retained as a disease-content question. | AAP; CDC |
| 75 | Special populations and risk communication | What misinformation about hand, foot, and mouth disease should parents avoid? | Excluded |  | Broad meta-question; not selected as a direct public consultation item. | CDC; NHS |
| 76 | Special populations and risk communication | What should parents do if online advice conflicts with a doctor’s recommendation? | Excluded |  | General health-literacy question; outside the core HFMD question set. | AAP; CDC |

# 4. Source Basis Used for Question Mapping

**Centers for Disease Control and Prevention (CDC):** About Hand, Foot, and Mouth Disease; HFMD symptoms and complications; causes and spread. Used for definition, symptoms, transmission, prevention, and return-to-school topics.

**World Health Organization / Western Pacific Region (WHO/WPRO):** HFMD surveillance and public-health materials. Used for disease overview, complications, outbreak relevance, and public-health framing.

**National Health Service (NHS):** Hand, foot and mouth disease patient guidance. Used for self-care, antibiotics, food/fluid advice, and when to seek care.

**American Academy of Pediatrics / HealthyChildren:** Parent-facing HFMD FAQs and symptom guidance. Used for diagnosis, treatment, contagiousness, prevention, dehydration, and school return.

**China CDC:** Health tips for HFMD. Used for incubation period, common manifestations, prevention, and China-relevant public-health framing.

**Clinical guidelines and evidence reviews:** Used for severe warning signs, diagnosis, hospital-referral considerations, EV71 vaccine scope, and limitations of vaccine cross-protection.

# 5.Final Confirmed HFMD Questions for Model Evaluation

The following table summarizes the final 20 standardized questions retained after the screening process.

| Final Question ID | Final Standardized Question |
| --- | --- |
| Final Q1 | What is Hand, Foot, and Mouth Disease (HFMD)? |
| Final Q2 | What are the typical symptoms of Hand, Foot, and Mouth Disease? |
| Final Q3 | How is Hand, Foot, and Mouth Disease transmitted? |
| Final Q4 | What is the course of Hand, Foot, and Mouth Disease? |
| Final Q5 | Is antibiotics needed for Hand, Foot, and Mouth Disease? |
| Final Q6 | How should fever be managed in a child with Hand, Foot, and Mouth Disease? |
| Final Q7 | What are the specific precautions to take during home isolation for Hand, Foot, and Mouth Disease? |
| Final Q8 | How can Hand, Foot, and Mouth Disease be prevented? |
| Final Q9 | What are the incubation period and infectious period of hand, foot, and mouth disease? |
| Final Q10 | How long after being cured of hand, foot, and mouth disease can a child return to kindergarten or school? |
| Final Q11 | What are the commonly used and effective disinfection methods for hand, foot, and mouth disease? |
| Final Q12 | Are there any special concerns for infants under 3 years old who contract the disease? |
| Final Q13 | Is there a vaccine to prevent hand, foot, and mouth disease? How effective is it? |
| Final Q14 | What is the cause of hand, foot, and mouth disease? |
| Final Q15 | What is the treatment for hand, foot, and mouth disease? |
| Final Q16 | The rash of hand, foot, and mouth disease. |
| Final Q17 | home remedies for hand, foot and mouth disease itching |
| Final Q18 | Monitoring the condition of severe hand, foot, and mouth disease. |
| Final Q19 | What should be noted for a child with hand, foot, and mouth disease receiving home care? |
| Final Q20 | Who is susceptible to hand, foot, and mouth disease? |
