# Supplementary material for: Evaluation of generative AI-driven chatbots as sources of consumer health information on hand, foot, and mouth disease: a cross-sectional comparative study of safety, accuracy, information quality, readability, and empathy
Source: Front Public Health. 2026 Aug 11;14:1924162. doi: 10.3389/fpubh.2026.1924162 (PMC13503565; doi:10.3389/fpubh.2026.1924162)
Supplement: Supplementary file 3 [file Supplementary_file_3.docx]

# Mapping Table Between HFMD Public Consultation Questions and Guideline-Based Reference Content

This document presents a detailed mapping framework linking standardized public consultation questions related to Hand, Foot, and Mouth Disease (HFMD) with evidence-based guideline content, systematic reviews, meta-analyses, clinical reviews, and expert recommendations. The purpose of this mapping process was to establish a transparent and reproducible reference framework for evaluating large language model responses in terms of accuracy, safety, reliability, readability, and empathy.

## Methodological Framework

- Candidate knowledge sources were identified through PubMed searches focusing on HFMD-related guidelines, systematic reviews, meta-analyses, and high-impact clinical reviews.
- Reference sources were prioritized based on recency, methodological rigor, public health relevance, and accessibility.
- Each question was mapped to one or more evidence domains, including etiology, epidemiology, symptom recognition, transmission, home care, supportive treatment, severe disease warning signs, vaccination, prevention, and public health management.
- The mapping framework was designed to facilitate the subsequent development of gold-standard reference answers for large language model evaluation.

## Detailed Question-to-Guideline Mapping Table

| Question ID | Standardized Question | Core Guideline / Knowledge Domain | Primary Supporting References |
| --- | --- | --- | --- |
| 1 | What is Hand, Foot, and Mouth Disease (HFMD)? | Definition, viral etiology, common pathogens, pediatric epidemiology | StatPearls; Narrative Review 2022; Current Status 2023 |
| 2 | What are the typical symptoms of HFMD? | Fever, oral ulcers, vesicular rash, buttocks involvement | Rapid Evidence Review; Narrative Review 2022 |
| 3 | How is HFMD transmitted? | Fecal-oral, respiratory droplet, direct contact transmission | StatPearls; Rapid Evidence Review |
| 4 | What is the course of HFMD? | Incubation, progression, spontaneous recovery timeline | Narrative Review 2022; Changing Epidemiology 2024 |
| 5 | Are antibiotics needed for HFMD? | Supportive treatment and avoidance of inappropriate antibiotics | Rapid Evidence Review; StatPearls |
| 6 | How should fever be managed in a child with HFMD? | Antipyretics, hydration, supportive care | Managing HFMD in Children 2024 |
| 7 | What precautions should be taken during home isolation? | Isolation, hygiene, surface disinfection | Rapid Evidence Review; Managing HFMD in Children |
| 8 | How can HFMD be prevented? | Handwashing, hygiene, vaccination, outbreak prevention | Rapid Evidence Review; EV71 Vaccine Review 2025 |
| 9 | What are the incubation and infectious periods of HFMD? | Typical incubation period and viral shedding | StatPearls; Changing Epidemiology 2024 |
| 10 | When can a child return to kindergarten or school after HFMD? | Return-to-school criteria and infectivity | Expert consensus; Public health guidance |
| 11 | What are effective disinfection methods for HFMD? | Environmental cleaning and fomites | Rapid Evidence Review |
| 12 | Are there special concerns for infants under 3 years old? | High-risk age groups and severe disease risk | Risk Factors Meta-analysis 2020 |
| 13 | Is there a vaccine for HFMD? How effective is it? | EV-A71 vaccine efficacy and protection | EV71 Vaccine Systematic Review 2025 |
| 14 | What causes HFMD? | Enterovirus etiology and molecular epidemiology | Current Status 2023 |
| 15 | What is the treatment for HFMD? | Supportive management and severe disease treatment | Narrative Review 2022; StatPearls |
| 16 | What does the HFMD rash look like? | Maculopapular/vesicular rash characteristics | Rapid Evidence Review |
| 17 | How can itching from HFMD be relieved at home? | Symptomatic relief and skin care | Managing HFMD in Children |
| 18 | How can severe HFMD be monitored? | Neurological and cardiopulmonary warning signs | Severe Outcomes Meta-analysis 2018 |
| 19 | What should be noted during home care for HFMD? | Hydration, feeding, fever monitoring, warning signs | Managing HFMD in Children |
| 20 | Who is susceptible to HFMD? | Children under 5 years, daycare outbreaks | StatPearls; Current Status 2023 |

**Key References Supporting the Evidence-Mapping Framework**

1.Guerra AM, Orille E, Waseem M. Hand, Foot, and Mouth Disease. In: StatPearls [Internet]. Treasure Island (FL): StatPearls Publishing; 2023. Available from: https://www.ncbi.nlm.nih.gov/books/NBK431082/

2.Leung AKC, Lam JM, Barankin B, Leong KF, Hon KL. Hand, Foot, and Mouth Disease: A Narrative Review. Recent Adv Inflamm Allergy Drug Discov. 2022;16(2):77-95. doi:10.2174/1570180820666221024095837

3.Saguil A, Kane SF, Lauters R, Mercado MG. Hand-Foot-and-Mouth Disease: Rapid Evidence Review. Am Fam Physician. 2019;100(7):408-414.

4.Zhu P, Ji W, Li D, et al. Current status of hand-foot-and-mouth disease. J Biomed Sci. 2023;30(1):15. doi:10.1186/s12929-023-00908-4

5.Faridi MMA, Kapoor S, Snigdha, Bhatnagar S. Managing hand-foot-mouth disease in children: More of counseling, less of medicines. J Family Med Prim Care. 2024;13:4090-4094. doi:10.4103/jfmpc.jfmpc_1444_23

6.Jones E, Pillay TD, Liu F, et al. Outcomes following severe hand foot and mouth disease: A systematic review and meta-analysis. Eur J Paediatr Neurol. 2018;22(5):763-773. doi:10.1016/j.ejpn.2018.04.007

7.Ni XF, Li X, Xu C, et al. Risk factors for death from hand-foot-mouth disease: A meta-analysis. Epidemiol Infect. 2020;148:e44. doi:10.1017/S0950268819002279

8.Yi Z, Pei S, Suo W, et al. Epidemiological characteristics, routine laboratory diagnosis, clinical signs and risk factors for hand-foot-and-mouth disease: A systematic review and meta-analysis. PLoS One. 2022;17(4):e0267716. doi:10.1371/journal.pone.0267716

9.Yan X, Liu Y, Chen F, et al. The efficacy and effectiveness of enterovirus A71 vaccines against hand, foot, and mouth disease: A systematic review and meta-analysis. PLoS One. 2025;20(5):e0323782. doi:10.1371/journal.pone.0323782

10.Kalam N, Balasubramaniam V. Changing Epidemiology of Hand, Foot, and Mouth Disease Causative Agents and Contributing Factors. Am J Trop Med Hyg. 2024;111(4):740-755. doi:10.4269/ajtmh.23-0852

11.Huang H, Deng L, Jia L, Zhu R. A clinical scoring system for pediatric hand-foot-mouth disease. BMC Infect Dis. 2021;21:722. doi:10.1186/s12879-021-06424-w

## Scientific Rationale for the Mapping Process

The mapping process was developed to ensure that each public consultation question corresponded to at least one evidence-based clinical or public health domain supported by authoritative literature. Questions involving fever management, antibiotics, home isolation, return-to-school criteria, severe neurological warning signs, and vaccine effectiveness were considered high-priority safety domains due to their potential impact on delayed medical care, misinformation, or inappropriate treatment behaviors.

Questions related to transmission, incubation period, prevention strategies, and environmental disinfection were mapped primarily to public health and infection control literature. Questions concerning severe disease monitoring were linked to systematic reviews and meta-analyses describing risk factors for neurological complications, pulmonary edema, and mortality.

This structured evidence-mapping framework improves methodological transparency and supports reproducibility in large language model evaluation studies focused on HFMD-related public health consultation.
