# Supplementary material for: Evaluation of generative AI-driven chatbots as sources of consumer health information on hand, foot, and mouth disease: a cross-sectional comparative study of safety, accuracy, information quality, readability, and empathy
Source: Front Public Health. 2026 Aug 11;14:1924162. doi: 10.3389/fpubh.2026.1924162 (PMC13503565; doi:10.3389/fpubh.2026.1924162)
Supplement: Supplementary file 4 [file Supplementary_file_4.docx]

# HFMD LLM Question-and-Answer Records (Clean Version)

This document preserves the original question-and-answer records from different large language models without modifying the content. Questions, answers, and model names are retained.

## **ChatGPT**

CHATGPT

**What is Hand, Foot, and Mouth Disease (HFMD)？**

**Answer：**

Hand, Foot, and Mouth Disease (HFMD) is a common viral infection that mainly affects infants and young children, though anyone can get it.

Here’s a simple explanation:

• Cause:

It’s usually caused by viruses from the enterovirus group, most commonly coxsackievirus A16or enterovirus 71.

• Main Features:

HFMD causes fever, mouth sores, and a rash on the hands, feet, and sometimes the buttocks or legs.

**• How It Spreads:**

The virus spreads easily through saliva, nasal mucus, fluid from blisters, or stool of an infected person.

• Duration:

Symptoms usually appear 3–6 days after exposure and go away in about 7–10 days.

• Seriousness:

HFMD is usually mild and recovers on its own, but good hygiene is important to prevent spreading it to others.

Would you like me to include a brief summary for students or parents (e.g., in one short paragraph)?

**2.What are the typical symptoms of Hand, Foot, and Mouth Disease?**

**Answer：:**

The typical symptoms of Hand, Foot, and Mouth Disease (HFMD) include:

1. Fever – usually the first sign of infection.

2. Sore throat – making it uncomfortable to eat or drink.

3. Painful mouth sores – small red spots that may blister and turn into ulcers, often found on the:

• Tongue

• Gums

• Inside of the cheeks

Skin rash – flat or raised red spots, sometimes with blisters, appearing on:

• Palms of the hands

• Soles of the feet

• Occasionally on the buttocks, knees, or elbows

Loss of appetite – because of mouth pain.

6. General tiredness or irritability – especially in young children.

These symptoms usually appear 3–6 days after infection and last about 7–10 days.

Would you like me to make a short table summarizing early vs. later symptoms of HFMD?

**3.How is Hand, Foot, and Mouth Disease transmitted?**

**Answer：**

Hand, Foot, and Mouth Disease (HFMD) spreads very easily from person to person, especially among young children.

Here’s how it’s transmitted:

1. Through respiratory droplets

• When an infected person coughs or sneezes, tiny droplets containing the virus can spread to others.

2. By contact with bodily fluids

• The virus is present in saliva, nasal mucus, fluid from blisters, and stool.

• Touching these fluids can spread the infection.

3. By touching contaminated objects or surfaces

• Toys, doorknobs, or tables that have the virus on them can pass it on when someone touches them and then touches their mouth, nose, or eyes.

4. Through close personal contact

• Hugging, kissing, or sharing cups, utensils, or towels with an infected person can spread the virus.

HFMD is most contagious during the first week of illness, but the virus can remain in stool for several weeks, meaning it can still spread even after symptoms disappear.

Would you like me to include a short diagram or list showing how HFMD spreads in a classroom or home setting?

**4.What is the course of Hand, Foot, and Mouth Disease?**

**Answer：**

The course of Hand, Foot, and Mouth Disease (HFMD) usually follows a clear and mild pattern that lasts about 7–10 days.

Here’s how it typically progresses:

1. Incubation period (3–6 days)

• This is the time between catching the virus and showing symptoms.

• The person feels fine during this stage but can already spread the virus.

2. Early symptoms (Day 1–2)

• Fever

• Sore throat

• Loss of appetite

• General tiredness or irritability

3. Appearance of mouth sores (Day 2–3)

• Small red spots appear inside the mouth (tongue, gums, inner cheeks).

• They may blister and become painful ulcers, making eating or drinking uncomfortable.

4. Rash develops (Day 3–5)

• A rash of red spots or small blisters appears on the palms, soles, and sometimes buttocksor legs.

• The rash may or may not itch.

5. Recovery (Day 7–10)

• Fever goes away.

• Mouth sores and rash heal without scarring.

• The person feels normal again.

Note: The virus can still be present in the stool for weeks after recovery, so good hygiene remains important even after symptoms disappear.

Would you like me to make a timeline chart showing each stage of the disease visually?

**5.Is antibiotics needed for Hand, Foot, and Mouth Disease?**

**Answer：**

No, antibiotics are not needed for Hand, Foot, and Mouth Disease (HFMD).

Here’s why:

• HFMD is caused by viruses — usually coxsackievirus A16 or enterovirus 71.

• Antibiotics only work against bacteria, not viruses.

• Because HFMD is a viral infection, antibiotics won’t speed recovery or prevent spread.

Treatment focuses on:

• Relieving fever and pain (with acetaminophen or ibuprofen)

• Drinking plenty of fluids to prevent dehydration

• Getting rest

**However, see a doctor if:**

• The fever is high or lasts more than a few days

• The child isn’t drinking enough fluids

• Blisters appear infected (red, swollen, or filled with pus) — in that case, a doctor might check for secondary bacterial infection, which could require antibiotics.

Would you like me to explain why overusing antibiotics can be harmful (for a classroom or health education note)?

**6.How should fever be managed in a child with Hand, Foot, and Mouth Disease?**

**Answer：**

To manage fever in a child with Hand, Foot, and Mouth Disease (HFMD) safely and effectively, follow these steps:

1. Monitor the fever

• Check the child’s temperature regularly.

• A mild fever (below 38.5°C / 101.3°F) is common and helps the body fight the infection.

2. Use fever medicine if needed

• Give acetaminophen (Tylenol) or ibuprofen (Motrin, Advil) to reduce fever and relieve discomfort.

• Follow the correct dose for the child’s age and weight.

• Do not give aspirin — it can cause Reye’s syndrome, a serious condition in children.

3. Keep the child hydrated

• Offer plenty of fluids like water, milk, or cold drinks.

• If mouth sores make drinking painful, try ice pops, smoothies, or cool soups.

• Watch for signs of dehydration (dry lips, fewer wet diapers, tiredness).

4. Encourage rest

• Let the child rest at home and avoid daycare or school until the fever is gone and the child feels better.

5. When to see a doctor

• Fever lasts more than 3 days, or is above 39°C / 102°F.

• The child is very irritable, drowsy, or vomiting.

• Shows signs of dehydration or difficulty swallowing.

Would you like me to make a parent-friendly fever care checklist you can print or share (for home or classroom use)?

**7.What are the specific precautions to take during home isolation for Hand, Foot, and Mouth Disease?**

**Answer：**

During home isolation for Hand, Foot, and Mouth Disease (HFMD), the goal is to prevent spreading the virus to other family members while keeping the sick child comfortable.

Here are the specific precautions to follow:

1. Keep the sick child at home

• Keep the child away from school, daycare, or playgrounds until:

• Fever is gone, and

• Blisters have dried or healed.

• HFMD is most contagious in the first week, but the virus can stay in stool for weeks — so hygiene is essential even after recovery.

2. Practice strict hand hygiene

• Wash hands often with soap and water for at least 20 seconds, especially:

• After changing diapers or helping the child use the toilet.

• After wiping the nose, touching saliva, or cleaning blisters.

• Before eating or preparing food.

• Adults and siblings should also follow these handwashing rules.

3. Clean and disinfect surfaces daily

• Disinfect toys, doorknobs, tabletops, and bathroom surfaces with a bleach solution or household disinfectant.

• Wash bedding, towels, and clothes in hot water and dry them thoroughly.

4. Avoid close contact

• Do not share cups, utensils, toothbrushes, towels, or bedding.

• Avoid kissing, hugging, or cuddling until the child is fully recovered.

5. Manage symptoms and comfort

• Keep the child hydrated and comfortable.

• Offer soft, cool foods to ease mouth pain.

• Give fever medicine if needed (acetaminophen or ibuprofen).

6. Handle diapers and waste safely

• Wear disposable gloves if possible when changing diapers.

• Seal used diapers in a plastic bag before throwing them away.

• Wash hands thoroughly afterward.

7. Watch for warning signs

Call a doctor if the child has:

• High or persistent fever

• Signs of dehydration (dry mouth, few wet diapers, lethargy)

• Blisters that look infected (red, swollen, pus-filled)

Would you like me to make a simple “Home Isolation Checklist” (one-page printable guide for parents or caregivers)?

**8.How can Hand, Foot, and Mouth Disease be prevented?**

**Answer：**

You can prevent Hand, Foot, and Mouth Disease (HFMD) mainly through good hygiene and avoiding close contact with infected individuals.

Here are the key prevention steps:

1. Wash hands frequently

• Wash hands with soap and water for at least 20 seconds, especially:

• After using the toilet or changing diapers

• After coughing, sneezing, or wiping a child’s nose

• Before eating or preparing food

• Teach children to wash their hands properly too.

2. Avoid close contact

• Do not share utensils, cups, towels, or toothbrushes.

• Avoid hugging, kissing, or close physical contact with someone who has HFMD.

3. Disinfect surfaces and objects

• Clean toys, doorknobs, tables, and other frequently touched surfaces every day with a disinfectant.

• Wash bedding and clothes in hot water if contaminated with saliva or stool.

4. Cover coughs and sneezes

• Use a tissue or elbow to cover the mouth and nose when coughing or sneezing.

• Dispose of tissues properly and wash hands right after.

5. Keep sick children at home

• Keep them away from school or daycare until:

• Fever is gone, and

• Blisters are healed or crusted.

6. Practice safe diaper handling

• Change diapers in a designated area.

• Dispose of them in sealed bags and wash hands afterward.

7. No vaccine (yet)

• There is no specific vaccine for HFMD (except in some countries for EV71-related HFMD).

• Hygiene remains the best protection.

Would you like me to summarize these steps in a colorful poster or checklist for school or home display?

**9. What are the incubation period and infectious period of hand, foot, and mouth disease?**

**Answer:**

The incubation period and infectious period of Hand, Foot, and Mouth Disease (HFMD) are important to know for controlling its spread.

Here’s a clear breakdown

1. Incubation period

• The incubation period is the time between catching the virus and showing symptoms.

• For HFMD, it’s usually 3–6 days.

• During this time, the person may not show symptoms but can already spread the virus to others.

2. Infectious period

• HFMD is most contagious during the first week of illness — when fever, mouth sores, and rash appear.

**• However, the virus can remain in the body for weeks, especially in stool, so:**

• A person may continue to spread the virus for days or even weeks after recovery.

• This is why good hygiene (especially handwashing after using the toilet or changing diapers) is still important even after symptoms go away.

Summary:

Would you like me to make a timeline diagram showing how the virus spreads from exposure to recovery?

**10. How long after being cured of hand, foot, and mouth disease can a child return to kindergarten or school?**

**Answer:**

A child can usually return to kindergarten or school once the following conditions are met:

Fever has completely resolved — The child should be fever-free for at least 24 hours without medication.

Blisters and mouth ulcers have healed or dried up — Open blisters can still spread the virus through contact.

The child feels well enough to participate in normal activities — No excessive fatigue, irritability, or poor appetite.

Typical timeline:

Most children recover in 7 to 10 days.

The virus may still be shed in stool for several weeks, but the risk of transmission is much lower once visible symptoms (fever, rash, mouth sores) are gone and good hygiene is maintained.

Practical recommendation:

In most cases, a child can safely return to school or daycare about 1 week after symptom onset, once they are symptom-free and cleared by a doctor or school policy.

Would you like me to summarize the return-to-school guidelines used in your country’s public health system (e.g., CDC for the U.S., or your local Ministry of Health)?

**11. What are the commonly used and effective disinfection methods for hand, foot, and mouth disease?**

**Answer:**

Effective disinfection is crucial in preventing the spread of Hand, Foot, and Mouth Disease (HFMD), as the viruses (mainly Coxsackievirus A16 and Enterovirus 71) can survive for days on surfaces and in bodily fluids.

Here’s a detailed breakdown of commonly used and effective disinfection methods:

1. Hand Hygiene

Wash hands frequently with soap and running water for at least 20 seconds, especially:

After diaper changes or toilet use

After cleaning nasal or mouth secretions

Before eating or preparing food

Alcohol-based hand sanitizers (≥60% alcohol) can be used if soap and water are unavailable — but washing is more effective if hands are visibly soiled.

2. Surface Disinfection

HFMD viruses can remain infectious on surfaces such as toys, doorknobs, tables, and toilet areas.

Effective disinfectants:

Chlorine-containing disinfectants (bleach-based)

Household bleach (e.g., 5% sodium hypochlorite) can be diluted:

1 part bleach to 99 parts water (≈500 ppm) for routine cleaning.

1 part bleach to 49 parts water (≈1,000 ppm) for surfaces contaminated by body fluids (saliva, stool, nasal secretions).

Apply disinfectant for at least 10 minutes, then rinse with clean water and dry.

3. Toys, Utensils, and Bedding

Wash toys, pacifiers, and utensils with soap and water, then disinfect using a mild bleach solution or by boiling (if heat-resistant).

Clothing, towels, and bedding contaminated with secretions or stool should be:

Washed separately in hot water with detergent.

Sun-dried or heat-dried thoroughly.

4. Toilet and Bathroom Cleaning

Clean toilets, potty chairs, sinks, and bathroom floors daily using a 1:49 bleach solution (≈1,000 ppm).

Always wear gloves when cleaning and wash hands immediately afterward.

5. Ventilation and Air Hygiene

Keep rooms well-ventilated to reduce viral concentration in the air.

Avoid air-conditioned environments with poor airflow during outbreaks.

Important Precautions

Always prepare bleach solutions fresh daily (they lose potency after 24 hours).

Never mix bleach with ammonia or acidic cleaners (can release toxic gas).

Store disinfectants safely, out of children’s reach.

Would you like me to give you a simple daily disinfection checklist (for home or childcare centers) to prevent HFMD spread?

**12. Are there any special concerns for infants under 3 years old who contract the disease?**

**Answer:**

Yes — infants and toddlers under 3 years old require special attention when they contract Hand, Foot, and Mouth Disease (HFMD), because they are more vulnerable to dehydration and, in rare cases, severe complications.

Here’s what caregivers should pay close attention to

1. Higher Risk of Dehydration

Painful mouth ulcers can make infants refuse milk, food, or water.

Monitor for signs of dehydration:

Dry lips or tongue

Fewer wet diapers (less than 3–4 in 24 hours)

Drowsiness, irritability

Sunken eyes or fontanelle (soft spot on the head)

**What to do:**

Offer small, frequent sips of water, milk, or diluted fluids.

Choose soft, cool foods (yogurt, porridge, mashed fruits).

Avoid acidic or spicy foods that irritate the mouth.

2. Fever Management

Infants may have a higher, more fluctuating fever.

Use paracetamol (acetaminophen) or ibuprofen (if age ≥6 months) for fever or discomfort — follow dosing instructions carefully.

Sponge with lukewarm water if fever is high, and ensure adequate hydration.

3. Watch for Warning Signs of Complications

Though rare, enterovirus 71 (EV71) infection can lead to serious complications such as meningitis, encephalitis, or myocarditis.

Seek medical attention immediately if you notice:

Persistent high fever (>39 °C) not responding to medication

Lethargy, irritability, or drowsiness

Jerky movements, trembling, limb weakness, or unsteady walking

Rapid breathing or a fast heart rate

Vomiting or refusal to feed continuously

4. Preventing Spread

Infants shed virus in saliva, stool, and nasal secretions for weeks.

Keep the child home during the contagious period (usually until blisters have dried).

Disinfect toys, bottles, and pacifiers daily.

Wash caregivers’ hands after every diaper change and before feeding.

5. Follow-up and Monitoring

Most infants recover in 7–10 days with good care at home.

Follow up with a pediatrician if symptoms persist beyond 10 days, or if new rashes/ulcers appear.

Would you like me to make a care plan checklist for parents of infants under 3 with HFMD (feeding, fever care, disinfection, and warning signs all in one page)? It can be printed or saved for reference.

**13.Is there a vaccine to prevent hand, foot, and mouth disease? How effective is it?**

**Answer:**

Excellent question — here’s a clear and complete answer:

1. Existence of HFMD Vaccines

Yes, there is a vaccine, but it is currently specific to one major virus type — Enterovirus 71 (EV71) — which can cause severe cases of hand, foot, and mouth disease (HFMD).

The EV71 vaccine is available in several Asian countries, including China, Taiwan, and Vietnam.

As of now, there is no globally approved HFMD vaccine (e.g., not yet available in the U.S. or most Western countries).

2. Why Only for EV71

HFMD can be caused by several viruses:

Coxsackievirus A16 (common, usually mild)

Enterovirus 71 (EV71) (can cause severe complications)

Coxsackievirus A6, A10, and others

The current vaccine targets EV71 only, because it’s the type most associated with neurological and severe complications (such as meningitis or encephalitis).

3. Vaccine Effectiveness

Clinical studies (mainly from China) show:

90–97% effectiveness in preventing EV71-related HFMD

80–90% reduction in risk of severe complications

No protection against HFMD caused by other viruses (like Coxsackie A16 or A6)

So, while the vaccine does not prevent all HFMD, it significantly reduces the risk of severe illness in young children.

4. Who Should Get It (Where Available)

Recommended for children aged 6 months to 5 years, especially under 3 years.

Usually given as two doses, one month apart.

Not part of the routine immunization schedule in many countries yet, but may be advised in regions with frequent EV71 outbreaks.

5. Current Situation

In the U.S. and most other countries, there’s no licensed HFMD vaccine yet.

Research is ongoing for multivalent vaccines that target multiple viruses (EV71, CA16, CA6) to provide broader protection.

Would you like me to show a comparison table of HFMD vaccines currently approved or in development worldwide (including manufacturers and clinical trial results)?

**14.What is the cause of hand, foot, and mouth disease?**

**Answer:**

Hand, Foot, and Mouth Disease (HFMD) is caused by a group of viruses belonging to the Enterovirus genus of the Picornaviridae family. These viruses commonly infect young children and are spread easily through contact with bodily fluids.

Here’s a breakdown

These viruses are not related to foot-and-mouth disease in animals (which affects livestock) — they are completely different viruses.

2. Nature of the Virus

Enteroviruses are small, non-enveloped RNA viruses.

They can survive well in the environment, especially in stool and contaminated surfaces.

Because they are non-enveloped, they are resistant to alcohol and many disinfectants — hence chlorine-based disinfectants are preferred.

**3. How Infection Happens**

Transmission occurs mainly through:

Fecal-oral route: contact with stool (e.g., diaper changes).

Respiratory droplets: coughing, sneezing.

Contact transmission: touching contaminated surfaces, toys, or hands.

Direct contact: with saliva, nasal secretions, or fluid from blisters.

After entering the body, the virus multiplies in the throat and intestines, then spreads through the bloodstream to the skin and mucous membranes — producing the typical mouth ulcers and rashes on hands, feet, and buttocks.

4. Incubation Period

Usually 3 to 7 days from exposure to first symptoms.

Would you like me to include a simple diagram showing how HFMD spreads and affects the body (for teaching or parent education purposes)?

**15. What is the treatment for hand, foot, and mouth disease?**

**Answer:**

Hand, Foot, and Mouth Disease (HFMD) is a viral infection, so there is no specific antiviral treatment.

Management focuses on relieving symptoms, preventing dehydration, and monitoring for complications.

Here’s a comprehensive overview

1. No Specific Cure

HFMD is self-limiting, meaning it usually goes away on its own in 7–10 days.

Antibiotics are not effective, since the cause is viral (not bacterial).

3. Preventing Dehydration

Dehydration is the most common complication in children due to painful mouth sores.

Give plenty of cool fluids.

Oral rehydration solutions (ORS) can help if intake is low.

Watch for warning signs: dry mouth, fewer wet diapers, drowsiness, sunken eyes.

4. When to Seek Medical Attention

Go to the doctor immediately if the child shows:

Persistent high fever (>39 °C) not responding to medicine

Vomiting or refusal to eat/drink

Drowsiness, irritability, confusion, or unusual behavior

Jerky movements, limb weakness, or unsteady walking

Rapid heartbeat, shortness of breath, or bluish lips

These could indicate complications (rare but serious), especially from Enterovirus 71 (EV71).

5. Home Care Tips

Keep the child at home until fever is gone and blisters are dry.

Disinfect toys, tables, and toilets daily (use bleach solution).

Ensure good ventilation and hand hygiene.

Let the child rest adequately.

Would you like me to create a parent-friendly care guide (1-page summary) with feeding advice, warning signs, and daily care steps for HFMD recovery?

16. The rash of hand, foot, and mouth disease.

**Answer:**

Here’s a clear explanation of the rash in Hand, Foot, and Mouth Disease (HFMD) — its appearance, location, and evolution

1. Typical Appearance

The rash usually begins as small red spots or macules, which then turn into:

Vesicles (small fluid-filled blisters), or

Pustules in some cases.

The blisters are generally:

2–5 mm in diameter

Have a grayish or whitish center

Surrounded by a reddish halo

They do not usually itch, but may be painful (especially on the hands and feet).

Blisters do not typically form pus unless there’s a bacterial infection.

After a few days, they dry up without scarring.

2. Common Locations

The rash has a distinctive distribution, which gives the disease its name:

3. Progression Over Time

4. Atypical Rash (Coxsackievirus A6 or A10)

May cause widespread rash beyond the hands and feet (on arms, legs, face, trunk).

Blisters may be larger, more numerous, or even peel as they heal.

Some children may experience temporary nail shedding (onychomadesis) a few weeks later — this looks alarming but is harmless and nails regrow normally.

**5. What Not to Do**

Do not pop the blisters, as this can lead to bacterial infection.

Avoid scratching, even if mildly itchy.

Keep skin clean and dry — use mild soap and water.

Would you like me to include pictures or illustrations showing the typical and atypical rashes of HFMD (for easier recognition and parent education)?

home remedies for hand, foot and mouth disease itching

**Answer：**

That’s a very practical question — many children with Hand, Foot, and Mouth Disease (HFMD) feel itchy or uncomfortable as their rash heals, even though HFMD blisters usually aren’t very itchy at first.

Here are safe and effective home remedies to relieve itching and soothe the skin 👇

1. Keep Skin Cool and Clean

Cool compress:

Apply a clean, cool, damp cloth to itchy areas for 5–10 minutes several times a day.

This helps reduce inflammation and itching naturally.

Cool baths:

Give a lukewarm or cool bath (not hot!) to soothe the skin.

You can add:

Colloidal oatmeal (1–2 tablespoons in bathwater)

Baking soda (1 tablespoon per gallon of water)

Pat dry gently afterward (do not rub).

2. Gentle Skin Care

Use mild, fragrance-free soap for bathing.

Avoid bubble baths or scented body washes.

Apply a thin layer of fragrance-free moisturizer (e.g., plain aloe vera gel or baby lotion) after bathing to prevent dryness and reduce irritation.

Keep nails trimmed short to prevent scratching and secondary infection.

3. Comfortable Clothing

Dress your child in loose, breathable cotton clothes to reduce sweating and friction.

Keep the environment cool and well-ventilated, as heat can worsen itching.

4. Over-the-Counter Relief (if needed)

If the itching is significant, you may use:

Oral antihistamines such as cetirizine or diphenhydramine (for older children) — but consult a pediatrician for proper dosing.

Calamine lotion or zinc oxide cream can also be gently applied to non-broken skin to calm itching.

Avoid topical steroid creams (like hydrocortisone) unless specifically prescribed by a doctor.

5. Avoid These

Don’t use menthol, camphor, or alcohol-based lotions, as they can irritate sensitive HFMD blisters.

Don’t pop or scratch blisters, which can lead to bacterial infection and scarring.

6. Duration

Itching typically appears as blisters begin to heal (around day 5–7).

It usually subsides completely within a few days.

Would you like me to provide a simple “HFMD skin care and comfort routine” checklist (morning, bath time, bedtime) for parents to follow at home?

18.Monitoring the condition of severe hand, foot, and mouth disease.

**Answer：**

That’s an excellent and important question — because most cases of Hand, Foot, and Mouth Disease (HFMD) are mild, but a small number (especially caused by Enterovirus 71, or EV71) can become severe or life-threatening if not recognized early.

Here’s how to monitor and manage the condition closely 👇

1. Why Monitoring Is Important

Severe HFMD can affect the nervous system, heart, or lungs — leading to complications such as:

Encephalitis (brain inflammation)

Meningitis

Myocarditis or heart failure

Pulmonary edema (fluid in lungs)

Early detection saves lives — because these complications develop rapidly, often within 2–5 days after fever onset.

2. Warning Signs of Severe HFMD

Caregivers should observe the child carefully, especially during the first 3–5 days of illness.

Seek immediate medical care if any of these appear:

Neurological (brain or nervous system) signs

Drowsiness, lethargy, or difficulty waking up

Persistent irritability or inconsolable crying

Tremors, jerky movements, twitching

Unsteady walking or weakness in limbs

Sudden startle response or continuous limb shaking

Severe headache, vomiting, or neck stiffness

Cardiac (heart) signs

Fast heartbeat even when resting

Pale or bluish lips

Cold, sweaty skin

Poor peripheral circulation (cold hands and feet)

Respiratory (lung) signs

Rapid breathing or shortness of breath

Grunting sounds while breathing

Repeated vomiting or inability to keep fluids down

Systemic warning signs

High fever >39°C that persists or spikes again after initial improvement

Poor appetite, refusal to drink, or signs of dehydration (dry lips, fewer wet diapers)

Sudden worsening after seeming improvement

3. Daily Monitoring Checklist for Parents

You can track the following every 4–6 hours:

4. When to Go to the Hospital Immediately

Emergency care is needed if any of the following occur:

Persistent high fever unresponsive to medication

Vomiting with signs of dehydration

Drowsiness, confusion, convulsions

Rapid breathing or blue lips

Cold, clammy extremities

Continuous crying or inability to sleep due to pain

At the hospital, doctors may monitor:

Heart rate, blood pressure, and oxygen levels

Neurological signs

Blood and CSF (spinal fluid) tests, if needed

5. After Recovery

Some children, especially after EV71 infection, may need follow-up to check for lingering neurological or cardiac effects.

Encourage rest, hydration, and balanced nutrition during recovery.

Summary

Would you like me to make a “Severe HFMD Monitoring Chart” (a printable table for parents or nurses to fill in daily temperature, symptoms, and alert signs)? It’s often used in hospitals and home care to detect deterioration early.

**19.What should be noted for a child with hand, foot, and mouth disease receiving home care?**

**Answer:**

That’s a very practical and important question — because most children with Hand, Foot, and Mouth Disease (HFMD) can recover safely at home with good care and hygiene.

Here’s a comprehensive list of what caregivers should note and do during home care 👇

1. Rest and Isolation

Keep the child at home until:

Fever has gone for at least 24 hours without medication.

Blisters have dried and crusted over.

Avoid daycare, school, or crowded places to prevent spreading the virus.

HFMD is most contagious in the first week, but the virus may still be shed in stool for up to 4–6 weeks — so maintain hygiene even after recovery.

2. Ensure Adequate Hydration

Mouth ulcers can make swallowing painful — dehydration is a key risk.

Offer frequent sips of:

Water

Milk

Diluted fruit juice

Oral rehydration solution (ORS), if appetite is poor

Cool, soft foods are best (porridge, yogurt, mashed fruit).

Avoid acidic or spicy foods that sting mouth ulcers (e.g., citrus, tomato, salty soups).

3. Manage Fever and Pain

Use acetaminophen (paracetamol) or ibuprofen as prescribed to ease fever and mouth pain.

Monitor temperature regularly (every 4–6 hours).

Sponge gently with lukewarm water if fever is high — no cold or alcohol sponging.

4. Maintain Good Hygiene

Wash hands with soap and running water:

After diaper changes or toilet use

Before meals or food preparation

After touching blisters, saliva, or nasal mucus

Use separate towels, utensils, and cups for the sick child.

Wash toys, bottles, and pacifiers daily.

Clean frequently touched surfaces with bleach solution (1:49 dilution ≈ 1,000 ppm chlorine).

Wash soiled clothes and bedding separately with detergent and dry in the sun.

5. Comfort and Skin Care

Dress the child in loose, breathable cotton clothing.

Keep skin clean and dry; avoid scratching blisters.

You can apply calamine lotion or zinc oxide cream to relieve mild itching.

Do not pop blisters — this increases infection risk.

6. Observe for Warning Signs (Seek Medical Help if Any)

Call or visit a doctor immediately if your child has:

Persistent high fever (>39°C) for more than 2 days

Poor feeding or refusal to drink

Drowsiness, irritability, or unusual sleepiness

Tremors, limb weakness, or unsteady walking

Fast breathing, blue lips, or cold hands and feet

Vomiting or continuous crying

These could be early signs of severe HFMD (e.g., caused by EV71).

7. After Recovery

Nails may peel or fall off (especially after Coxsackie A6 infection) — this is temporary and harmless.

Maintain good hygiene for several more weeks.

Encourage rest and healthy eating to rebuild strength.

Summary Table

Would you like me to make a printable “HFMD Home Care Guide for Parents” — a one-page sheet with feeding tips, fever care, disinfection steps, and warning signs? It’s very useful for home caregivers and daycare centers.

20.Who is susceptible to hand, foot, and mouth disease?

**Answer:**

Excellent question — understanding who is most susceptible to Hand, Foot, and Mouth Disease (HFMD) helps with both prevention and early detection.

Here’s a clear explanation

1. Main Susceptible Group: Young Children

Infants and children under 5 years old are most susceptible.

Their immune systems are still developing, and many have no prior immunity to the viruses that cause HFMD.

Outbreaks often occur in nurseries, daycare centers, and kindergartens, where close contact and shared toys make transmission easy.

Especially high risk:

Children 6 months to 3 years old

Those who attend crowded childcare settings

Children who put toys or fingers in their mouths frequently

2. Older Children and Adults

Older children and adults can also become infected, but usually:

They have milder or no symptoms (because of partial immunity from previous exposure).

**However, they can still spread the virus to others — especially to infants or uninfected peers.**

3. Immunity and Reinfection

After infection, the body develops immunity to that specific virus type (e.g., Coxsackie A16 or EV71).

**However, since multiple viruses can cause HFMD, a child can:**

Recover from one infection (e.g., Coxsackie A16),

Then get infected again later by another (e.g., EV71 or A6).

Therefore, reinfection is possible, though symptoms are often milder the second time.

Other Susceptible Groups

5. Seasonal and Environmental Factors

HFMD occurs year-round, but peaks in warm, humid months — typically:

Spring to autumn in temperate climates

Year-round in tropical regions

Outbreaks are common during summer months and in places with poor ventilation or hygiene.

Would you like me to follow up with a chart showing how HFMD spreads among children and families (including the main routes of transmission and prevention checkpoints)? It’s great for educational or daycare use.

## **Copilot**

**What is Hand, Foot, and Mouth Disease (HFMD)？**

**Answer：**

Hand, Foot, and Mouth Disease (HFMD) is a highly contagious viral illness, most common in children under 5, that causes fever, painful mouth sores, and a rash on the hands and feet. It is usually mild, resolving within 7–10 days, but spreads quickly in schools and childcare settings.

**•What HFMD Is？**

Cause: HFMD is caused by enteroviruses, most often coxsackievirus A16 and enterovirus 71 (EV71).

Who Gets It: Mainly children under 5 years old, though older children and adults can also be infected.

Contagiousness: Spreads easily through close contact, respiratory droplets, saliva, fluid from blisters, and contaminated surfaces.

• Symptoms

Fever and sore throat

Painful mouth sores (small blisters inside the mouth)

Skin rash with red spots or blisters, typically on hands, feet, and sometimes buttocks

Loss of appetite and irritability in young children

Symptoms usually last 7–10 days and are mild.

•Prevention

Frequent handwashing with soap and water

Disinfecting surfaces and toys regularly

Avoiding close contact (hugging, sharing utensils) with infected individuals

Keeping sick children home from school or daycare until fully recovered

•Complications (Rare)

Dehydration due to painful mouth sores making swallowing difficult

In rare cases, neurological complications (especially with EV71 infections), such as viral meningitis or encephalitis

• Hong Kong Context

HFMD is seasonal in Hong Kong, with peaks often in spring and summer.

The Centre for Health Protection (CHP) monitors outbreaks and advises parents to keep children home if symptoms appear.

• Bottom line: HFMD is common, contagious, and usually mild. Good hygiene and keeping sick children at home are the best ways to prevent spread.

Would you like me to also explain how HFMD differs from chickenpox and measles, since parents often confuse these illnesses due to their rashes?

**2.What are the typical symptoms of Hand, Foot, and Mouth Disease?**

**Answer：:**

Typical symptoms of Hand, Foot, and Mouth Disease (HFMD) include fever, painful mouth sores, and a rash on the hands, feet, and sometimes buttocks or groin. The illness is usually mild, lasting 7–10 days, but can cause discomfort and irritability in young children.

•Common Symptoms of HFMD

Fever and flu-like signs:

Fever (often the first symptom)

Sore throat

Feeling unwell or tired

Reduced appetite or refusal to eat/drink

Painful mouth sores:

Begin as small red spots, usually on the tongue, gums, or inside of cheeks

Develop into blisters that can be painful, making swallowing difficult

Skin rash:

Red spots or blisters on palms of hands, soles of feet, and sometimes buttocks or groin

Rash may look pink, red, or darker than surrounding skin depending on skin tone

Blisters can be greyish or lighter than surrounding skin and may be painful

Other signs in children:

Irritability

Drooling (due to mouth pain)

Eating or drinking less, which can lead to dehydration

• Timeline of Symptoms

Day 1–3: Fever, sore throat, loss of appetite

Day 3–5: Mouth sores appear, followed by rash on hands/feet

Day 7–10: Symptoms usually resolve without complications

• When to Seek Medical Help

If your child has persistent high fever, is unable to drink fluids, shows signs of dehydration (dry mouth, fewer wet diapers), or if symptoms worsen.

Rarely, HFMD caused by enterovirus 71 (EV71) can lead to serious complications like viral meningitis or encephalitis.

• Bottom line: HFMD typically presents with fever, mouth sores, and a rash on hands and feet. It is usually mild, but parents should watch for dehydration and consult a doctor if symptoms are severe or prolonged.

**3.How is Hand, Foot, and Mouth Disease transmitted?**

Answer：Hand, Foot, and Mouth Disease (HFMD) spreads very easily through close contact, contaminated surfaces, and bodily fluids such as saliva, nasal secretions, and blister fluid. Children in schools and daycare centers are especially vulnerable because the virus can linger on hands, toys, and shared items.

**How HFMD Is Transmitted**

Person-to-person contact

Direct contact with an infected person’s nose or throat secretions (saliva, mucus, nasal discharge).

Contact with fluid from blisters on the skin.

Exposure to stool of infected individuals (common in diaper-changing settings).

Respiratory droplets

Coughing and sneezing release virus particles into the air, which can infect others nearby.

Contaminated surfaces and objects

Toys, doorknobs, tables, and other frequently touched surfaces can harbor the virus.

The virus can survive for hours to days on surfaces, making regular cleaning essential.

Community settings

Outbreaks often occur in schools, nurseries, and playgrounds where children share close spaces.

In Hong Kong, seasonal peaks are common in spring and summer, with the Centre for Health Protection monitoring clusters.

Infectious Period

HFMD is most contagious during the first week of illness, but the virus can remain in stool for several weeks after symptoms disappear.

This means children may still spread the virus even after they look well.

🛡️ Prevention Tips

Frequent handwashing with soap and water, especially after diaper changes or toilet use.

Disinfecting toys and surfaces daily in childcare settings.

Avoiding close contact (hugging, kissing, sharing utensils) with infected individuals.

Keeping sick children home until fever and blisters resolve.

Bottom line: HFMD spreads through saliva, nasal secretions, stool, blister fluid, and contaminated surfaces. Good hygiene and keeping sick children out of group settings are the most effective ways to stop transmission.

Would you like me to also outline how long children should stay home from school or daycare after HFMD, based on Hong Kong health guidelines?

**4.What is the course of Hand, Foot, and Mouth Disease?**

**Answer：**

The course of Hand, Foot, and Mouth Disease (HFMD) usually lasts about 7–10 days, beginning with mild flu-like symptoms and progressing to mouth sores and skin rash before resolving on its own.

Typical Course of HFMD

Incubation period (3–6 days)

After exposure, the virus takes several days before symptoms appear.

Children may seem healthy but can already be contagious.

Early stage (Days 1–2)

Fever, sore throat, and reduced appetite.

General feeling of being unwell.

Acute stage (Days 3–5)

Painful mouth sores develop (tongue, gums, inside cheeks).

Rash appears on hands, feet, and sometimes buttocks or groin.

Irritability and drooling are common in young children due to mouth pain.

Recovery stage (Days 7–10)

Fever subsides, sores and rash begin to heal.

Most children recover fully without complications.

⚠️ Possible Complications (Rare)

Dehydration: Painful mouth sores may make swallowing difficult.

Neurological issues (especially with EV71 infections): Viral meningitis or encephalitis, though uncommon.

Key Points

HFMD is self-limiting—it resolves without specific treatment.

Children are most contagious in the first week, but the virus can remain in stool for weeks after recovery.

Good hygiene practices help prevent spread during and after illness.

Bottom line: HFMD typically follows a short, predictable course—starting with fever, then mouth sores and rash, and ending in recovery within about 10 days.

Would you like me to create a day-by-day timeline chart of HFMD symptoms, so it’s easier to visualize how the illness progresses?

**5.Is antibiotics needed for Hand, Foot, and Mouth Disease?**

**Answer：**

No, antibiotics are not needed for Hand, Foot, and Mouth Disease (HFMD).

HFMD is caused by viruses (most commonly coxsackievirus A16 and enterovirus 71), and antibiotics only work against bacterial infections. Using antibiotics for HFMD would not help and could even cause unnecessary side effects or antibiotic resistance.

**What Helps Instead**

Rest and fluids: Encourage plenty of water to prevent dehydration, especially if mouth sores make swallowing painful.

Fever and pain relief: Over-the-counter medicines (paracetamol/acetaminophen or ibuprofen) may be used to ease discomfort, but only under guidance from a healthcare professional.

Soft foods: Cold drinks, smoothies, or soft foods can reduce pain when eating.

Good hygiene: Frequent handwashing and disinfecting surfaces to prevent spread.

When to See a Doctor

If your child has persistent high fever, severe mouth pain, or signs of dehydration (dry mouth, fewer wet diapers, lethargy).

If symptoms worsen or do not improve after about a week.

Bottom line: HFMD is a viral illness, so antibiotics are not useful. Care focuses on comfort, hydration, and hygiene until the illness resolves naturally.

Would you like me to also explain the difference between antibiotics and antiviral medicines, since HFMD is viral and sometimes parents wonder if antivirals are an option?

**6.How should fever be managed in a child with Hand, Foot, and Mouth Disease?**

**Answer：**

Fever in a child with Hand, Foot, and Mouth Disease (HFMD) is usually mild and short-lived, but it can make the child uncomfortable. Since HFMD is caused by a virus, the goal is comfort and hydration, not antibiotics.

🌡️ Managing Fever in HFMD

Monitor temperature: Keep track of the child’s fever with a thermometer.

Light clothing & cool environment: Dress the child in breathable clothes and keep the room comfortably cool.

Fluids: Encourage plenty of water, milk, or oral rehydration solutions. Cold drinks or ice pops can soothe mouth pain and help prevent dehydration.

Rest: Ensure the child gets enough sleep and quiet time.

Medications

Paracetamol (acetaminophen) or ibuprofen may be used to reduce fever and relieve discomfort.

Always follow doctor’s instructions or the dosage on the medicine label.

Avoid aspirin in children due to the risk of Reye’s syndrome.

When to Seek Medical Help

Fever persists for more than 3 days.

Child refuses fluids or shows signs of dehydration (dry mouth, fewer wet diapers, lethargy).

Fever is very high (≥ 39°C) or accompanied by unusual symptoms (stiff neck, confusion, difficulty breathing).

Bottom line: Manage HFMD fever with rest, fluids, light clothing, and fever-reducing medicines if needed. Watch closely for dehydration or prolonged fever, and consult a doctor if symptoms worsen.

Would you like me to also share a day-by-day care checklist for parents—covering fever, rash, and mouth sores—so you have a practical guide to follow at home?

**7.What are the specific precautions to take during home isolation for Hand, Foot, and Mouth Disease?**

**Answer：**

During home isolation for Hand, Foot, and Mouth Disease (HFMD), the main goal is to prevent the virus from spreading to other family members and to keep the sick child comfortable. Here are the specific precautions to follow:

Hygiene Precautions

Frequent handwashing: Wash hands with soap and water after diaper changes, toilet use, coughing, sneezing, or touching blisters.

Disinfect surfaces: Clean toys, doorknobs, tables, and shared items daily with household disinfectant.

Laundry care: Wash clothes, bedding, and towels separately in hot water.

Isolation Measures

Stay home: Keep the child out of school, daycare, and playgrounds until fever and blisters resolve.

Limit contact: Avoid hugging, kissing, or sharing utensils with siblings or other family members.

Separate personal items: Assign the child their own cups, plates, and towels.

Comfort & Care

Hydration: Offer plenty of fluids—cold drinks, smoothies, or ice pops can ease mouth pain.

Soft foods: Provide easy-to-swallow foods to reduce discomfort from mouth sores.

Rest: Ensure the child gets enough sleep and quiet time.

Duration of Precautions

Children are most contagious in the first week, but the virus can remain in stool for weeks.

Continue strict hygiene even after symptoms improve to reduce risk of transmission.

Bottom line: During HFMD home isolation, focus on hand hygiene, disinfecting, separating personal items, and keeping the child home until recovery. This protects both the child and the rest of the household.

Would you like me to also share a practical checklist for parents (daily cleaning, symptom monitoring, and return-to-school guidance) so you have a step-by-step routine to follow at home?

**8.How can Hand, Foot, and Mouth Disease be prevented?**

**Answer：**

Preventing Hand, Foot, and Mouth Disease (HFMD) relies on good hygiene and limiting exposure, since the illness spreads easily among children. Here are the key precautions:

Hygiene Practices

Frequent handwashing with soap and water, especially after diaper changes, toilet use, coughing, or sneezing.

Teach children proper hand hygiene—singing a short song while washing can help them wash long enough.

Cover mouth and nose when coughing or sneezing, then wash hands immediately.

Environmental Cleaning

Disinfect toys, doorknobs, and surfaces daily with household disinfectant.

Wash bedding, towels, and clothes in hot water to kill lingering virus particles.

Avoid sharing utensils, cups, or towels among family members.

Isolation Measures

Keep sick children home from school or daycare until fever and blisters resolve.

Limit close contact (hugging, kissing, sharing food) with siblings or other children.

Assign personal items (cups, toothbrushes, towels) to the sick child.

Community Awareness

HFMD outbreaks are common in nurseries, schools, and playgrounds, especially in spring and summer in Hong Kong.

Parents should monitor for symptoms and act quickly to isolate children if signs appear.

Bottom line: HFMD prevention centers on hand hygiene, disinfecting, avoiding shared items, and keeping sick children home. These steps protect both the child and the wider community.

Would you like me to also create a parent-friendly checklist (daily cleaning, symptom monitoring, and return-to-school guidance) so you have a practical routine to follow at home?

**9. What are the incubation period and infectious period of hand, foot, and mouth disease?**

**Answer:**

Hand, Foot, and Mouth Disease (HFMD) has a short incubation period and a longer infectious period, which explains why it spreads so easily among children.

Incubation Period

3–6 days after exposure to the virus.

During this time, the child may look healthy but the virus is multiplying inside the body.

Symptoms (fever, sore throat, mouth sores, rash) typically appear after this period.

Infectious Period

Most contagious in the first week of illness (when fever, mouth sores, and rash are present).

The virus can still be shed in stool for several weeks after recovery, meaning children may spread HFMD even after they look well.

Virus can also linger in respiratory secretions for 1–2 weeks.

Practical Implications

Children should stay home from school or daycare until fever and blisters resolve.

Parents should continue strict handwashing and surface cleaning for several weeks after recovery to reduce risk of spread.

Since siblings often share toys and utensils, separating personal items is important during and after illness.

Bottom line: HFMD incubates for about 3–6 days, is most contagious in the first week, but can still spread for weeks afterward through stool and secretions. Good hygiene practices remain essential even after symptoms improve.

Would you like me to create a timeline chart showing incubation, symptom onset, peak contagiousness, and recovery, so you can visualize the full course of HFMD more clearly?

**10. How long after being cured of hand, foot, and mouth disease can a child return to kindergarten or school?**

**Answer:**

A child recovering from Hand, Foot, and Mouth Disease (HFMD) can usually return to kindergarten or school after the fever has subsided and the blisters have dried or healed.

General Guidance

Fever-free: The child should be free of fever for at least 24 hours without medication.

Healing blisters: Mouth sores and skin blisters should be healing or dried, as fresh blisters can still spread the virus.

Energy level: The child should be well enough to participate in normal school activities.

Infectious Considerations

HFMD is most contagious in the first week, but the virus can remain in stool for weeks after recovery.

Because of this, schools and health authorities focus on visible symptoms (fever, blisters) rather than complete elimination of the virus.

Good hygiene (handwashing, cleaning toys, avoiding shared utensils) remains important even after the child returns.

📍 Hong Kong Health Advice

The Centre for Health Protection (CHP) advises that children with HFMD should stay home until fever and blisters resolve.

Parents should continue strict hygiene practices at home and teach children proper handwashing before returning to group settings.

Bottom line: A child can return to kindergarten or school once they are fever-free, blisters have healed, and they feel well enough to join activities. Parents should still maintain good hygiene, since the virus can linger in stool for weeks.

Would you like me to create a parent checklist for “return-to-school readiness” (covering fever, blisters, energy level, and hygiene habits) so you have a clear step-by-step guide?

**11. What are the commonly used and effective disinfection methods for hand, foot, and mouth disease?**

**Answer:**

The most effective disinfection methods for Hand, Foot, and Mouth Disease (HFMD) involve thorough cleaning with chlorine-based solutions (like diluted bleach), alcohol-based disinfectants, and regular washing of fabrics in hot water. UV-C light can also inactivate the virus on surfaces. These steps are critical because HFMD-causing enteroviruses can survive for days on toys, tables, and other shared items.

Commonly Used Disinfection Methods

1. Chemical Disinfectants

Chlorine bleach (sodium hypochlorite):

Widely recommended for HFMD.

A solution of 1 part household bleach to 99 parts water (about 500 ppm) is effective for general surface cleaning.

Stronger solutions (1:49 dilution) may be used for heavily contaminated areas like toilets.

Alcohol-based disinfectants (≥70% ethanol or isopropyl alcohol):

Effective for small surfaces (e.g., toys, doorknobs, phones).

Quick-drying and convenient for frequent use.

Hydrogen peroxide-based cleaners:

Useful for broader surface disinfection, especially in schools and daycare centers.

2. Non-Chemical Methods

Hot water laundering:

Wash clothes, bedding, and towels at ≥60°C to kill viruses.

UV-C light technology:

Proven to inactivate enteroviruses on hard surfaces.

Increasingly used in healthcare and childcare facilities.

Practical Home Measures

Daily cleaning of toys, tables, and doorknobs with diluted bleach or alcohol wipes.

Separate laundry for the sick child, washed in hot water.

Disinfect toilets and diaper-changing areas more frequently.

Avoid sharing utensils, cups, and towels among family members.

⚠️ Key Risks & Considerations

Bleach safety: Always dilute properly and keep out of reach of children.

Surface compatibility: Alcohol and bleach may damage certain materials (e.g., wood, fabric). Test before use.

Persistence of virus: HFMD viruses can remain in stool for weeks, so continue disinfection even after the child appears recovered.

Bottom line: Use diluted bleach for general surfaces, alcohol-based disinfectants for small items, and hot water laundering for fabrics. Combine these with strict hygiene practices to effectively reduce HFMD transmission at home or in schools.

Would you like me to create a step-by-step daily disinfection checklist for parents (covering toys, laundry, toilets, and shared items) so it’s easier to follow at home during an HFMD outbreak?

**12. Are there any special concerns for infants under 3 years old who contract the disease?**

**Answer:**

Yes — infants under 3 years old with Hand, Foot, and Mouth Disease (HFMD) require special attention because they are more vulnerable to complications and have difficulty communicating their discomfort.

Special Concerns for Infants <3 Years

Dehydration risk: Painful mouth sores can make swallowing difficult. Infants may refuse milk or fluids, leading to dehydration quickly.

Feeding challenges: They may drool excessively, cry during feeding, or stop eating altogether. Parents need to watch closely for reduced intake.

Higher irritability: Infants cannot explain pain, so they may be unusually fussy, clingy, or have trouble sleeping.

Complications: Though rare, infants are more susceptible to severe forms of HFMD (especially with EV71 virus), which can cause neurological issues like viral meningitis or encephalitis.

Rapid spread: Infants often share toys and close contact in daycare settings, making outbreaks harder to control.

Extra Precautions for Infants

Hydration monitoring: Check for wet diapers (at least every 6–8 hours). Fewer wet diapers may signal dehydration.

Offer soft, cool foods/drinks: Cold milk, yogurt, or pureed foods can be easier to swallow.

Pain relief: Fever-reducing medicines (paracetamol/acetaminophen) may be used under pediatric guidance.

Strict hygiene: Wash hands after diaper changes, disinfect toys, and avoid sharing utensils.

Medical attention: Seek care if the infant has persistent high fever, refuses all fluids, shows lethargy, or has unusual symptoms (stiff neck, seizures, breathing difficulty).

Bottom line: Infants under 3 with HFMD need closer monitoring for hydration, feeding, and unusual symptoms. While most recover fully, they are at higher risk of complications, so parents should be vigilant and consult a doctor promptly if concerns arise.

Would you like me to create a parent-friendly “red flag checklist” for infants with HFMD — signs that mean you should go to the doctor immediately?

**13.Is there a vaccine to prevent hand, foot, and mouth disease? How effective is it?**

**Answer:**

Yes, there is a vaccine for Hand, Foot, and Mouth Disease (HFMD), but it only targets the enterovirus 71 (EV71) strain, which is responsible for the most severe cases. Studies in China and other parts of Asia show the EV71 vaccine is highly effective—reducing severe HFMD and related hospitalizations by over 90%. However, it does not protect against other common HFMD viruses like coxsackievirus A16.

HFMD Vaccine Overview

Type: Inactivated EV71 vaccine (several versions licensed in China since 2016).

Target: EV71 virus, a major cause of severe HFMD and neurological complications.

Availability: Widely used in mainland China; not yet part of routine immunization in Hong Kong or many other countries.

Effectiveness

Clinical trials & meta-analyses show:

>90% efficacy against severe EV71-related HFMD.

Significant reduction in hospitalization and mortality rates.

Real-world studies confirm a sharp decline in EV71 outbreaks after vaccine introduction.

Limitations

Strain-specific: Protects only against EV71, not against other HFMD-causing viruses (e.g., coxsackievirus A16, A6).

Not universal: No global HFMD vaccine yet; EV71 vaccines are mainly available in China and some Asia-Pacific regions.

Timing: Best given to children under 5, who are most at risk of severe HFMD.

Practical Implications for Parents

In Hong Kong, no routine HFMD vaccine program exists yet. Prevention still relies on hand hygiene, disinfection, and isolation.

Families traveling to mainland China may encounter EV71 vaccination as an option for young children.

Even vaccinated children can still catch HFMD from non-EV71 strains, but the risk of severe complications is greatly reduced.

Bottom line: The EV71 vaccine is highly effective against severe HFMD caused by EV71, but it does not cover all HFMD viruses. In Hong Kong, prevention still depends on hygiene and outbreak control, while vaccination is an option mainly in mainland China.

Would you like me to summarize the current vaccination recommendations for children in Hong Kong versus mainland China, so you know whether it’s something you can access locally?

**14.What is the cause of hand, foot, and mouth disease?**

**Answer:**

Hand, Foot, and Mouth Disease (HFMD) is caused by viruses belonging to the enterovirus group, most commonly coxsackievirus A16 and enterovirus 71 (EV71).

Main Causes

Coxsackievirus A16

The most frequent cause of HFMD worldwide.

Usually leads to mild illness with fever, mouth sores, and rash.

Enterovirus 71 (EV71)

Less common but more serious.

Associated with severe complications such as viral meningitis, encephalitis, or even paralysis in rare cases.

Other enteroviruses

Coxsackievirus A6 and A10 can also cause HFMD.

These strains may produce more widespread rashes or atypical symptoms.

**How the Virus Spreads**

Through saliva, nasal secretions, stool, and blister fluid.

Via contaminated surfaces and toys.

Most contagious in the first week of illness, but virus can remain in stool for weeks after recovery.

Bottom Line

HFMD is caused by enteroviruses, especially coxsackievirus A16 and EV71. While most cases are mild, EV71 infections can be more severe, which is why hygiene and outbreak control are so important in childcare settings.

Would you like me to also break down the differences in symptoms between Coxsackievirus A16 and EV71 infections, since parents often worry about how to tell a mild case from a potentially severe one?

**15. What is the treatment for hand, foot, and mouth disease?**

**Answer:**

Treatment for Hand, Foot, and Mouth Disease (HFMD) is supportive only, since it is caused by viruses and there is no specific antiviral cure. The focus is on relieving symptoms, preventing dehydration, and maintaining good hygiene until the illness resolves naturally.

Supportive Care

Fever and pain relief

Paracetamol (acetaminophen) or ibuprofen may be used to reduce fever and ease discomfort.

Avoid aspirin in children due to risk of Reye’s syndrome.

Hydration

Encourage plenty of fluids to prevent dehydration.

Cold drinks, smoothies, or ice pops can soothe mouth pain.

For infants, monitor wet diapers closely.

Diet adjustments

Soft, bland foods (yogurt, porridge, mashed fruit) are easier to swallow.

Avoid spicy, salty, or acidic foods that irritate mouth sores.

Rest

Ensure the child gets adequate sleep and quiet time to recover.

Preventing Spread During Recovery

Keep the child home from school or daycare until fever and blisters resolve.

Wash hands frequently, especially after diaper changes or toilet use.

Disinfect toys, surfaces, and shared items daily.

When to Seek Medical Help

Persistent high fever (>3 days).

Signs of dehydration (dry mouth, fewer wet diapers, lethargy).

Severe headache, stiff neck, confusion, or difficulty breathing (possible complications with EV71 infections).

Bottom line: HFMD treatment is symptom management and supportive care—fluids, rest, pain relief, and hygiene. Most children recover fully within 7–10 days without complications.

Would you like me to create a day-by-day home care checklist for parents (covering fever, rash, mouth sores, and hygiene) so you have a practical routine to follow during your child’s recovery?

16. The rash of hand, foot, and mouth disease.

**Answer:**

The rash of Hand, Foot, and Mouth Disease (HFMD) is one of its hallmark features, appearing alongside fever and mouth sores.

Characteristics of the HFMD Rash

Location:

Commonly on the palms of the hands and soles of the feet.

May also appear on the buttocks, legs, arms, or genital area.

Appearance:

Starts as small red spots that may turn into blisters.

Blisters are often oval or elongated, with a grayish center and red edges.

They are usually not itchy, but can be painful, especially on the feet.

Progression:

Rash typically appears 1–2 days after fever and mouth sores.

Blisters dry up within 7–10 days without scarring.

**How It Differs from Other Childhood Rashes**

Chickenpox: Rash spreads all over the body, is very itchy, and blisters appear in waves.

Measles: Rash starts on the face and spreads downward, accompanied by cough, runny nose, and red eyes.

HFMD: Rash is localized mainly to hands, feet, and sometimes buttocks, with mouth sores as a key distinguishing feature.

When to Seek Medical Advice

If the rash spreads widely, looks infected (pus, swelling), or is accompanied by persistent high fever.

If the child refuses fluids or shows signs of dehydration.

Bottom line: The HFMD rash consists of red spots and blisters on the hands, feet, and sometimes buttocks, appearing a few days after fever. It heals on its own within about a week.

Would you like me to create a visual comparison chart of HFMD rash vs. chickenpox vs. measles, so it’s easier to distinguish them at a glance?

home remedies for hand, foot and mouth disease itching

**Answer：**

For itching caused by the rash in Hand, Foot, and Mouth Disease (HFMD), there’s no specific cure, but several home remedies can help ease discomfort and keep the child more comfortable:

Home Remedies for HFMD Rash Itching

Cool compresses: Apply a clean, cool damp cloth to itchy areas for a few minutes to soothe irritation.

Oatmeal baths: Add colloidal oatmeal or finely ground oats to lukewarm bathwater. This can calm itching and soften the skin.

Baking soda baths: A small amount of baking soda in bathwater may help relieve itchiness.

Loose, breathable clothing: Cotton clothes reduce friction and allow the skin to breathe.

Keep nails short: Trim the child’s fingernails to prevent scratching and secondary infection.

Moisturizers: Apply gentle, fragrance-free moisturizers to keep skin hydrated and reduce irritation.

Important Notes

Avoid scratching blisters, as this can lead to infection.

Do not use steroid creams or medicated ointments unless prescribed by a doctor.

Keep baths lukewarm, not hot, since hot water can worsen itching.

When to Seek Medical Help

If blisters look infected (red, swollen, pus-filled).

If itching is severe and disrupts sleep or feeding.

If the child develops a high fever or unusual symptoms.

Bottom line: HFMD itching can be eased with cool compresses, oatmeal or baking soda baths, moisturizers, and loose clothing. The rash heals on its own in about a week, but watch for signs of infection.

Would you like me to put together a step-by-step “comfort care routine” for children with HFMD—covering fever, mouth sores, and rash—so you have a practical daily guide at home?

18.Monitoring the condition of severe hand, foot, and mouth disease.

**Answer：**

For severe Hand, Foot, and Mouth Disease (HFMD), careful monitoring is essential because complications—though rare—can progress quickly, especially in young children. Here’s what parents and caregivers should watch for:

Key Areas to Monitor

Fever

Track temperature regularly.

Persistent high fever (>39°C) or fever lasting more than 3 days needs medical review.

Hydration status

Check for wet diapers (at least every 6–8 hours).

Watch for dry mouth, sunken eyes, or refusal to drink—all signs of dehydration.

Neurological symptoms (linked to EV71 infections)

Severe headache, stiff neck, sensitivity to light.

Confusion, irritability, drowsiness, or seizures.

Difficulty walking or unusual limb weakness.

Respiratory and circulatory signs

Rapid breathing, shortness of breath.

Cold hands/feet, poor circulation, or bluish lips.

Skin and rash

Monitor blisters for signs of infection (redness, swelling, pus).

Widespread rash beyond typical hands, feet, and mouth may need evaluation.

Red Flags Requiring Immediate Medical Attention

Persistent vomiting or refusal to eat/drink.

Lethargy or inability to wake the child easily.

Seizures or sudden jerking movements.

Breathing difficulties or bluish skin.

Signs of meningitis (stiff neck, severe headache).

Monitoring Routine at Home

Daily checks: Temperature, fluid intake, urine output.

Symptom diary: Note changes in rash, behavior, or feeding.

Hygiene vigilance: Continue disinfecting surfaces and washing hands to prevent spread.

Bottom line: Severe HFMD requires close observation of fever, hydration, neurological signs, and breathing. Any red flag symptoms should prompt immediate medical care, as complications can escalate quickly.

Would you like me to create a parent-friendly monitoring checklist (with daily tick boxes for fever, fluids, rash, and behavior) so you can track your child’s condition more systematically at home?

**19.What should be noted for a child with hand, foot, and mouth disease receiving home care?**

**Answer:**

When a child with Hand, Foot, and Mouth Disease (HFMD) is cared for at home, parents should focus on comfort, hydration, hygiene, and monitoring for warning signs. Here are the key points to note:

Symptom Care

Fever & pain relief: Use paracetamol (acetaminophen) or ibuprofen if needed, following medical guidance.

Mouth sores: Offer soft, cool foods and drinks (yogurt, smoothies, porridge) to reduce pain when eating.

Itching/rash: Keep skin clean, use cool compresses or oatmeal baths, and dress the child in loose cotton clothing.

Hydration & Nutrition

Encourage frequent fluids to prevent dehydration.

Monitor wet diapers (at least every 6–8 hours for infants).

Avoid acidic, spicy, or salty foods that irritate mouth sores.

Hygiene & Prevention

Wash hands thoroughly after diaper changes, toilet use, or contact with saliva/blisters.

Disinfect toys, doorknobs, and surfaces daily.

Do not share utensils, cups, towels, or toothbrushes.

Keep the child home from school or daycare until fever and blisters resolve.

Monitoring & Red Flags

Seek medical attention if you notice:

Persistent high fever (>39°C) or fever lasting more than 3 days.

Refusal to drink fluids or signs of dehydration (dry mouth, lethargy, fewer wet diapers).

Neurological symptoms: severe headache, stiff neck, confusion, seizures.

Breathing difficulties or bluish lips.

Blisters that look infected (red, swollen, pus-filled).

Bottom line: Home care for HFMD means comfort, hydration, hygiene, and close monitoring. Most children recover in 7–10 days, but parents should be alert for red flag symptoms that require medical attention.

Would you like me to create a daily home care checklist (covering fever, fluids, rash, hygiene, and red flags) so you can track your child’s recovery step by step?

20.Who is susceptible to hand, foot, and mouth disease?

**Answer:**

Hand, Foot, and Mouth Disease (HFMD) can affect anyone, but certain groups are especially susceptible.

Most Susceptible Groups

Children under 5 years old

The highest risk group, especially toddlers and preschoolers.

Their immune systems are still developing, and they often have close contact in daycare or kindergarten.

Infants under 3 years old

More vulnerable to complications like dehydration or neurological issues (especially with EV71 infections).

Feeding difficulties due to painful mouth sores make them particularly at risk.

Other Susceptible Groups

Older children and teenagers

Less common, but outbreaks can occur in schools.

Adults

Usually mild if infected, but parents and caregivers can catch HFMD from children.

Adults may experience flu-like symptoms and rash, though less severe.

Risk Factors

Group settings: Daycare, kindergartens, playgrounds, and schools where children share toys and have close contact.

Poor hygiene: Infrequent handwashing or sharing utensils increases transmission.

Seasonal outbreaks: More common in warm, humid months (spring and summer in Hong Kong).

Bottom line: HFMD mainly affects children under 5, especially those in daycare or kindergarten, but older children and adults can also get it. Good hygiene and limiting exposure in group settings are key to prevention.

Would you like me to also outline why adults sometimes get more severe symptoms than expected, even though HFMD is usually thought of as a childhood illness?

## **Doubao**

**What is Hand, Foot, and Mouth Disease (HFMD)？**

**Answer：**

Hand, Foot, and Mouth Disease (HFMD) is a common viral infection that mostly affects infants and children under 5 years old, but can also occur in older children and adults.

Simple Explanation

Caused mainly by Coxsackievirus A16 and Enterovirus 71 (EV71)

Spread through close contact, saliva, blister fluid, feces, or contaminated surfaces

Usually mild and self-healing within 7–10 days

Typical Symptoms

Small, painful sores in the mouth

Rash or blisters on hands, feet, and sometimes buttocks

Mild fever, sore throat, loss of appetite, tiredness

Severe Cases (rare)

In a small number of cases (especially with EV71), it may lead to complications like meningitis, encephalitis, or myocarditis, which require immediate medical care.

Would you like a simpler Chinese version or more prevention tips?

**2.What are the typical symptoms of Hand, Foot, and Mouth Disease?**

**Answer：:**

Typical symptoms of HFMD usually appear 3–7 days after infection and last about 7–10 days:

Fever

Mild to high fever, often one of the first signs.

Mouth sores

Painful small red spots or blisters inside the cheeks, tongue, gums, and throat, which can make eating and drinking painful.

Skin rash & blisters

Flat or raised red spots, often with small blisters:

on the palms of hands

on the soles of feet

sometimes on buttocks, knees, or elbows

Other common symptoms

Sore throat

Loss of appetite

Tiredness, irritability (especially in young children)

Drooling (due to mouth pain)

Severe symptoms (rare but dangerous) include persistent high fever, convulsions, severe headache, stiff neck, vomiting, rapid breathing, or lethargy — these need immediate medical help.

**3.How is Hand, Foot, and Mouth Disease transmitted?**

**Answer：：**

Hand, Foot, and Mouth Disease (HFMD) is highly contagious and spreads mainly through close contact with an infected person. The primary transmission routes are:

1. Person‑to‑person contact (most common)

Direct contact with nasal discharge, saliva, blister fluid, or feces of an infected person.

Close personal contact: hugging, kissing, sharing utensils/cups, or touching contaminated hands.

2. Respiratory droplets

Coughing or sneezing releases droplets that can be inhaled or land on surfaces.

3. Fecal‑oral route

The virus can remain in feces for several weeks after symptoms improve.

Infection often occurs via contaminated hands touching the mouth.

4. Contaminated objects and surfaces

Touching doorknobs, toys, tables, or other items with virus on them, then touching eyes, nose, or mouth.

Common viruses involved

Most cases are caused by Coxsackievirus A16 and Enterovirus 71 (EV‑A71).

In short: HFMD spreads easily through touch, saliva, blisters, stool, and contaminated surfaces.

Would you like me to also explain the typical symptoms and incubation period in simple English?

**4.What is the course of Hand, Foot, and Mouth Disease?**

**Answer：**

Here’s a clear explanation of the typical course (progression) of Hand, Foot, and Mouth Disease (HFMD):

Course of Hand, Foot, and Mouth Disease

Incubation period

Usually 3 to 7 days after exposure to the virus.

No symptoms yet, but the person is already contagious.

Initial phase (onset)

Fever often appears first (mild to moderate).

Sore throat, loss of appetite, tiredness, and fussiness in young children.

Oral symptoms

Painful red spots and blisters develop inside the mouth, on the tongue, gums, and cheeks.

These can make eating, drinking, and swallowing uncomfortable.

Skin rash phase

One to two days after fever starts, a rash appears:

Small red spots or blisters on palms of hands

On soles of feet

Sometimes on buttocks, knees, or elbows

The rash is usually not itchy.

Recovery phase

Fever usually resolves in 2–3 days.

Mouth sores and skin blisters gradually dry up and heal within 7–10 days without leaving scars.

Most children recover completely with no complications.

Severe cases (rare)

A small number of children (especially those infected with EV71) may develop severe symptoms such as persistent high fever, convulsions, vomiting, lethargy, rapid heartbeat, or difficulty breathing. These require emergency medical treatment immediately.

**5.Is antibiotics needed for Hand, Foot, and Mouth Disease?**

**Answer：**

No, antibiotics are NOT needed or effective for Hand, Foot, and Mouth Disease (HFMD).

Simple reason:

HFMD is caused by a virus (enterovirus, coxsackievirus).

Antibiotics only kill bacteria — they do not work against viruses.

When might antibiotics be used?

Only if a secondary bacterial infection develops, such as:

Bacterial skin infection from scratching blisters

Bacterial throat infection or pneumonia

In most uncomplicated cases, no antibiotics are required. Treatment focuses on:

Relieving fever and pain (paracetamol / acetaminophen, ibuprofen)

Keeping the child hydrated

Rest

**6.How should fever be managed in a child with Hand, Foot, and Mouth Disease?**

**Answer：**

Here’s a clear, practical guide to managing fever in a child with HFMD:

Safe fever management

Use fever-reducing medicine if needed

Paracetamol (acetaminophen) or ibuprofen can be given to lower fever and relieve pain.

Follow the dosage based on your child’s age and weight.

Do not give aspirin — it can cause serious side effects.

Keep the child comfortable

Dress in light, loose clothing.

Keep the room at a comfortable temperature.

Encourage rest.

Prevent dehydration

Offer plenty of fluids: water, oral rehydration solution, milk, or cool soup.

Mouth sores may hurt, so give small amounts often.

Sponge bathing (only if very uncomfortable)

Use lukewarm water (not cold, not alcohol).

Do not use cold water or ice — this can cause shivering and raise body temperature.

When to see a doctor immediately

Fever higher than 39°C (102.2°F) that does not come down with medicine.

Fever lasting more than 3 days.

Child is very sleepy, difficult to wake, or very fussy.

Signs of severe dehydration: no tears, dry mouth, little or no urine for 6–8 hours.

Convulsions (seizures), stiff neck, rapid breathing, or vomiting repeatedly.

**7.What are the specific precautions to take during home isolation for Hand, Foot, and Mouth Disease?**

**Answer：**

Here are clear, practical precautions for home isolation with Hand, Foot, and Mouth Disease (HFMD):

1. Isolation time

Keep the child home from kindergarten/school for at least 7–10 days from the start of symptoms.

Isolation can end when fever is gone, blisters have crusted, and no new rash appears.

2. Personal hygiene

Wash hands thoroughly with soap and water, especially:

before eating / preparing food

after using the toilet, changing diapers

after touching blisters or mouth sores

Avoid kissing, sharing food, drinks, or utensils with the sick child.

3. Disinfection of items

Disinfect frequently touched surfaces: table, doorknob, toy, phone, etc.

Use chlorine-containing disinfectant or boiling water.

Boil or soak tableware, towels, clothes in hot water.

Toys should be cleaned and disinfected daily.

4. Clothing and bedding

Change clothes and bed sheets frequently.

Wash separately from other family members’ laundry.

Dry in sunlight if possible.

5. Waste disposal

Seal used tissues, diapers, waste carefully in plastic bags.

Throw away immediately to avoid spreading virus.

6. Avoid contact with others

Do not receive visitors during isolation.

Keep the sick child away from:

other children

pregnant women

elderly people

anyone with weak immunity

7. Caregiver protection

Caregivers should also wash hands often and avoid touching their own mouth, nose, eyes.

If possible, have only one fixed person take care of the sick child.

8. Environmental ventilation

Open windows for ventilation 2–3 times a day, about 30 minutes each time.

Keep the room clean and dry.

**8.How can Hand, Foot, and Mouth Disease be prevented?**

**Answer：**

Here are the key ways to prevent Hand, Foot, and Mouth Disease (HFMD):

1. Wash hands frequently

Wash hands with soap and running water, especially:

before eating and preparing food

after using the toilet

after changing diapers

after touching public objects or toys

Hand sanitizer can be used when soap is not available.

2. Avoid close contact with sick people

Do not kiss, hug, or share eating utensils, cups, towels with children who have HFMD.

Keep sick children home from kindergarten or school until fully recovered.

3. Clean and disinfect regularly

Disinfect frequently touched surfaces: tables, doorknobs, toys, handrails.

Boil tableware, bottles, and toys or use disinfectant.

Keep the living environment clean and well‑ventilated.

4. Maintain good personal and living habits

Do not put hands or dirty objects into the mouth.

Cover the mouth and nose when coughing or sneezing.

Avoid crowded, poorly ventilated public places during outbreaks.

5. Strengthen immunity

Ensure adequate rest, balanced nutrition, and regular outdoor activities.

Keep the child’s immune system strong.

6. Get vaccinated (for high‑risk types)

The EV71 vaccine can effectively prevent severe HFMD caused by Enterovirus 71.

It is recommended for children aged 6 months to 5 years.

**9. What are the incubation period and infectious period of hand, foot, and mouth disease?**

**Answer:**

Incubation Period

Generally: 2–10 days

Average: 3–5 days

Infectious Period

Peak contagiousness:

First week after symptom onset (highest viral load in throat, saliva, blister fluid).

Active shedding (clinically contagious):

From a few days before symptoms until ~1 week after illness onset.

Prolonged viral shedding (lower risk):

Throat/nasopharynx: virus can be shed for 3–4 weeks

Feces: virus can persist for 6–12 weeks (infectious potential remains but much lower)

In short:

Incubation: 2–10 days (avg 3–5 days)

Highly infectious: ~1 week around symptom onset

Shedding can continue: weeks to months in stool

**10. How long after being cured of hand, foot, and mouth disease can a child return to kindergarten or school?**

**Answer:**

According to general clinical and kindergarten/school health guidelines (consistent with CDC and Chinese pediatric recommendations):

When can a child go back to kindergarten/school?

A child can usually return when all of the following are met:

All fever has been gone for at least 24 hours (without fever-reducing medicine).

All mouth sores, blisters, and rashes have crusted over or completely healed (no new blisters, open lesions, or oozing).

The child feels well enough to participate in normal activities.

Typical time frame

Usually 6–8 days after symptoms start

Most children are able to return to kindergarten/school after about 1 week from onset

Important note

Even after recovery, the virus can still be shed in stool for several weeks, but the child is no longer considered contagious to others once symptoms are fully resolved. Schools and kindergartens only restrict attendance during the symptomatic and highly infectious period.

**11. What are the commonly used and effective disinfection methods for hand, foot, and mouth disease?**

**Answer:**

Hand, foot, and mouth disease is mainly caused by enteroviruses (such as Coxsackie A16 and EV71), which are sensitive to heat, ultraviolet light, oxidizing disinfectants, etc., but not sensitive to alcohol-based hand sanitizer alone.

Here are the commonly used and truly effective disinfection methods:

1. Hand disinfection (most important)

Wash hands with soap + running water for ≥20 seconds (after touching children, changing diapers, before meals, after using toilet)

Alcohol sanitizer alone is not reliable for enteroviruses.

If soap is unavailable: use chlorine-containing hand sanitizer / disinfectant wipes instead.

2. Environmental & object disinfection

Chlorine-containing disinfectants (most effective)

Dilute 84 disinfectant / household bleach to

1:100 ~ 1:200 (500–1000 mg/L available chlorine)

Wipe or soak: toys, tabletops, doorknobs, floors, bed rails

Contact time: ≥30 minutes, then rinse with clean water

Heat disinfection

Boil items for ≥15 minutes

Steam or high-temperature disinfection cabinet

Suitable for: tableware, toys, baby bottles, small cloth items

Sun & UV

Direct sunlight for ≥4 hours

UV lamp irradiation (for rooms/toys)

3. Clothing, bedding, towels

Wash with hot water + detergent

Dry in the sun

Do not mix with healthy family members’ clothes

4. Fecal & vomit contamination

Use 1000–2000 mg/L chlorine disinfectant

Cover first → disinfect → clean

Avoid splashing; wear gloves

5. Air

Natural ventilation 2–3 times a day, 30+ minutes each

Air disinfectants are generally not necessary

Summary of effective vs. useless

✅ Effective

Soap & water

Chlorine disinfectants (84, bleach)

Boiling / high temperature

Sunlight / UV

❌ Not effective against HFMD virus

Alcohol alone

Iodine

Lysol-type phenolic disinfectants

Simple water rinse

If you need, I can give you a simple daily disinfection schedule for kindergartens / homes.

**12. Are there any special concerns for infants under 3 years old who contract the disease?**

**Answer:**

Yes, infants under 3 years old are the high-risk group for severe hand, foot, and mouth disease (HFMD), and they need extra close observation — much more so than older children.

Key Special Concerns

Higher risk of severe/critical cases

Severe HFMD often involves the nervous system: encephalitis, meningitis, neurogenic pulmonary edema, circulatory failure.

The main culprit is usually EV71 infection, which is far more dangerous in toddlers.

Difficulty eating and drinking → dehydration risk

Severe mouth ulcers make them refuse milk/food.

Watch for:

Little or no urine for 6+ hours

Dry lips, sunken eyes, lethargy

Dehydration can worsen condition rapidly.

Early warning signs of severe illness (must seek emergency help immediately)

If any appear within 1–4 days after fever onset, go to hospital right away:

Persistent high fever (>39°C) that doesn’t respond to medicine

Lethargy, excessive sleepiness, or extreme irritability

Repeated vomiting

Shaking, twitching, limb weakness, unsteady walking

Rapid or labored breathing, cold sweats, pale or bluish skin

Rapid heartbeat, cold extremities

More likely to have complications

Myocarditis

Pulmonary edema / hemorrhage

Seizures

Sequelae in rare severe cases

Practical Care Advice

Do not force-feed; give small amounts of cool, mild food/liquid frequently.

Avoid aspirin (risk of Reye’s syndrome).

Isolate strictly — no contact with other babies.

If the child is under 1 year old, even mild symptoms should be evaluated by a doctor promptly.

Summary

Children under 3 are the key warning group.

Mild HFMD can be managed at home, but you must watch closely for severe signs — any warning symptom means emergency medical care immediately.

**13.Is there a vaccine to prevent hand, foot, and mouth disease? How effective is it?**

**Answer:**

There is a vaccine for hand, foot, and mouth disease (HFMD), and it is highly effective, especially against severe cases. Here is a clear overview:

**What the vaccine protects against**

The vaccine targets Enterovirus 71 (EV71), the main cause of severe HFMD and most deaths.

It does not protect against other HFMD-causing viruses (e.g., Coxsackie A16).

**How effective it is**

High protection against EV71-related HFMD:

Clinical trials show 90%–97.9% efficacy in the first year.

Real-world studies confirm ~90% effectiveness.

Protection remains strong for at least 2 years.

Critical protection against severe illness:

Reduces severe cases by ~43%.

Provides 100% protection against severe EV71 cases in infants (6–11 months).

Key details

Approved in China since 2015 (global first).

Two doses, 1 month apart, for children ≥6 months old.

Classified as a "Category II vaccine" (voluntary).

Common side effects: mild fever, redness/swelling at the injection site (usually resolves within 3 days).

Bottom line

The EV71 vaccine is safe and effective. It is the best way to prevent severe HFMD, especially for infants under 3 years old.

**14.What is the cause of hand, foot, and mouth disease?**

**Answer:**

Hand, foot, and mouth disease (HFMD) is a viral infectious disease caused by several types of enteroviruses.

Main pathogens

Enterovirus 71 (EV71)

The most dangerous one

Mainly causes severe cases, including encephalitis, pulmonary edema, and even life‑threatening conditions.

Coxsackievirus A16 (CV‑A16)

The most common cause of mild HFMD

Usually causes only mild fever, rash, and mouth ulcers.

Other common types

Coxsackievirus A6 (CV‑A6)

Coxsackievirus A10 (CV‑A10)

Other enterovirus serotypes

**How it spreads**

Direct contact: touching blister fluid, saliva, feces of an infected person

Respiratory droplets: coughing, sneezing, talking

Indirect contact: touching contaminated toys, tableware, doorknobs, towels, etc.

Who is most susceptible

Children under 5 years old, especially infants under 3

Easily outbreaks in kindergartens and nurseries

In short:

HFMD is caused by enteroviruses, with EV71 being the most harmful and Coxsackie A16 the most common.

**15. What is the treatment for hand, foot, and mouth disease?**

**Answer:**

Most hand, foot, and mouth disease (HFMD) is mild and self-healing, meaning it gets better on its own. There is no specific antiviral drug to kill the virus quickly — treatment focuses on relieving symptoms and preventing complications.

1. General treatment (for mild cases, done at home)

Rest and isolate the child to avoid spreading the virus.

Drink plenty of fluids (cool water, porridge water, electrolyte solution) to prevent dehydration.

Eat light, cool, soft, non-irritating food (avoid spicy, hot, hard, sour food) to reduce mouth pain.

Keep skin clean; do not break blisters (to avoid infection).

2. Symptomatic relief

Fever: Use paracetamol (acetaminophen) or ibuprofen if high fever or discomfort.

Mouth pain: Some hospitals use topical pain-relief or mucosal repair gels for ulcers.

Skin rash: Generally no special ointment needed; keep dry and clean.

3. Medications to avoid

No antibiotics — HFMD is viral, antibiotics do not work and should not be used.

Do not use aspirin (risk of Reye’s syndrome in children).

4. Treatment for severe cases (hospital only)

If the child develops dangerous signs (continuous high fever, convulsions, vomiting, poor spirit, rapid breathing, etc.), they must be hospitalized immediately.

Treatment includes:

Antiedema therapy for the brain

Cardiopulmonary support

Monitoring and control of vital signs

Intravenous fluids and supportive care

Simple summary

Mild HFMD: No special cure — rest, fluids, fever control, mouth care.

Severe HFMD: Emergency hospital treatment to protect the heart, brain, and lungs.

16. The rash of hand, foot, and mouth disease.

**Answer:**

Hand, foot, and mouth disease rash has very typical features — it’s easy to recognize once you know the pattern.

Typical appearance of HFMD rash

Location: Mainly on the hands, feet, mouth, and buttocks (sometimes knees, elbows).

Shape: Small red papules or herpetic vesicles (tiny blisters).

Feeling: Usually not itchy or only mildly itchy; blisters generally do not break easily or form pus.

Mouth rash: Painful small ulcers or blisters inside the cheeks, tongue, gums, and throat.

Course of the rash

Appears 1–2 days after fever

Lasts about 3–5 days

Then gradually dries up, scabs, and falls off

Usually leaves no scars or pigmentation after healing

Special types

Some strains (like Coxsackie A6) can cause a wider rash on the arms, legs, and face, and may desquamate (skin peeling) or even nail shedding later.

Blisters on the buttocks are very common in young children.

Key reminders

Do not puncture blisters — can cause secondary bacterial infection.

No special ointment is needed; just keep clean and dry.

If rash becomes red, swollen, pus-filled, or spreads widely with high fever, seek medical attention.

17.home remedies for hand, foot and mouth disease itching

**Answer：**

Here are safe, gentle home remedies to relieve itching from hand, foot, and mouth disease — all suitable for young children, with no harsh ingredients:

1. Cool compress (safest & most effective)

Use a clean, soft cloth soaked in cool (not ice-cold) water

Wring it out and apply to itchy rash/blisters for 5–10 minutes

Repeat 3–5 times a day

Calms itching quickly and reduces redness.

2. Calamine lotion

Apply a thin layer to itchy skin (avoid broken blisters & mouth)

Dries blisters slightly and relieves itching

Safe for toddlers when used externally only.

3. Aloe vera gel

Pure, colorless aloe vera gel (no added alcohol/fragrance)

Soothes itching and helps repair skin

Good for mild, irritated rash.

4. Oatmeal bath

Grind plain oatmeal into fine powder

Mix with warm (not hot) bath water

Let child soak for 10–15 minutes

Great for widespread itching on body, hands, feet.

5. Keep skin cool & loose

Dress in lightweight, cotton clothes

Avoid sweating (heat makes itching worse)

Do not scratch — trim and file nails short to prevent breaking blisters.

**What to AVOID**

Do NOT use alcohol, iodine, or strong disinfectants on skin

Do NOT use adult anti-itch creams containing steroids unless prescribed by a doctor

Do NOT pop blisters (risk of infection and scarring)

If itching is severe, rash becomes infected (pus, swelling), or the child is very upset, consult a doctor for safe topical medicine.

Monitoring the condition of severe hand, foot, and mouth disease.

**Answer：**

Monitoring for severe hand, foot, and mouth disease is critical, especially in children under 3. Below is a clear, practical monitoring guide for early warning signs and what to check at home.

Core monitoring period

First 1–4 days after fever onset

This is when severe cases usually develop.

1. Vital signs to monitor closely

Temperature

Watch for fever over 39°C that lasts >24 hours

Fever that does not come down with fever reducers

Pulse & breathing

Rapid, heavy breathing, gasping, or chest indrawing

Fast, weak pulse

2. Nervous system warning signs (most important)

If any appear, go to hospital immediately:

Lethargy, extreme sleepiness, or cannot be fully woken up

Abnormal irritability, continuous crying, cannot be comforted

Repeated vomiting (not just vomiting once after eating)

Trembling, shaking, limb twitching, or startle-like jerks

Limp limbs, unable to stand or hold head up

Stiff neck

3. Circulation warning signs (dangerous stage)

Cold, clammy hands and feet even when warm

Pale, gray, or bluish lips/skin

Dizziness, listlessness, near-fainting

4. Other red flags

Refusing to drink or eat for more than 8 hours

No urine output for 6+ hours (sign of severe dehydration)

Rapidly worsening rash with swelling or pus

**How to monitor at home**

Check mental state every 1–2 hours during the day

Measure temperature every 4 hours

Record fluid intake and urine output

Keep the child calm and avoid overexcitement

When to go to the emergency room right away

Any one of the following:

Persistent high fever >39°C

Repeated vomiting

Lethargy or extreme irritability

Twitching, shaking, or weakness

Breathing problems

Cold extremities + pale skin

Summary

Mild HFMD can be cared for at home, but any change in mental state or repeated vomiting means the condition may be turning severe — do not wait, seek emergency medical help immediately.

**19.What should be noted for a child with hand, foot, and mouth disease receiving home care?**

**Answer:**

Here’s a clear, practical list of key home care notes for a child with hand, foot, and mouth disease — easy to follow and covers all critical points:

1. Isolation & infection control

Isolate the child at home; do not go to kindergarten, playground, or visit others until fully recovered.

Avoid close contact with other children or pregnant women.

Adults should wash hands immediately after caring for the child.

Separate tableware, towels, toys, and bedding; disinfect daily.

2. Fever management

Monitor temperature every 4–6 hours.

Use paracetamol or ibuprofen for high fever or discomfort.

Do not use aspirin.

Keep the room cool and avoid heavy wrapping.

3. Mouth care (very important)

Mouth ulcers cause pain; give cool, mild, liquid or soft food (porridge, milk, yogurt).

Avoid spicy, hot, sour, hard, and salty foods.

Encourage frequent small sips of water to keep the mouth clean.

Do not force eating; focus on preventing dehydration.

4. Skin rash care

Trim nails short to prevent scratching and breaking blisters.

Wear loose, soft cotton clothes.

Bathe with warm water; do not scrub the rash.

Do not pop blisters or apply alcohol/iodine.

Mild itching can be relieved with cool compresses or calamine lotion.

5. Hydration monitoring

Ensure the child drinks enough.

Watch for dehydration:

No urine for more than 6 hours

Dry lips, sunken eyes

Lethargy

If these appear, seek medical help immediately.

6. Strict observation for severe signs

During the first 3–5 days of illness, check frequently for:

Persistent high fever >39°C that doesn’t respond to medicine

Lethargy, extreme irritability, or convulsions

Repeated vomiting

Cold, clammy hands and feet

Rapid or difficult breathing

Any of these = go to hospital right away.

7. Diet & rest

Light, digestible diet.

Ensure adequate rest; avoid strenuous activity.

Do not take antibiotics — they are useless for viral HFMD.

8. Returning to kindergarten

Only go back when:

No fever for at least 24 hours

All blisters have crusted and healed

No new rash appears

In short:

Home care focuses on fever control, preventing dehydration, protecting the skin, and watching for severe symptoms — most mild cases recover safely within 7–10 days.

20.Who is susceptible to hand, foot, and mouth disease?

**Answer:**

Hand, foot, and mouth disease mainly infects young children, and the susceptibility is very clear:

1. Most susceptible group

Children under 5 years old are the main susceptible population.

Especially children under 3 years old have the highest risk of severe illness.

Infants 6 months to 2 years old are most frequently infected.

2. Why are young children more susceptible?

Immune system is not yet fully developed.

No previous immunity to related enteroviruses.

Poor hand hygiene habits, often putting hands or toys in the mouth.

3. Can older children and adults get infected?

Yes, but much less often.

Older children and adults may also be infected, but most have mild or no obvious symptoms.

Even without symptoms, they can still carry and spread the virus.

4. Other susceptible situations

Children in kindergartens, nurseries, and daycare centers are prone to cluster outbreaks.

Those who have close contact with a confirmed case without proper protection.

Simple summary

High-risk: Kids under 5, especially under 3

Low-risk: Older children and adults

Key point: Adults can be silent carriers and infect babies unknowingly.

## **DeepSeek**

**What is Hand, Foot, and Mouth Disease (HFMD)？**

**Answer：**

**1.What is Hand, Foot, and Mouth Disease (HFMD)？**

Hand, Foot, and Mouth Disease (HFMD) is a common contagious illness primarily caused by enteroviruses, most commonly Coxsackievirus A16 and Enterovirus 71 (EV71). It most frequently affects infants and children under 5 years old and can easily spread in group settings like preschools and daycare centers.

As the name suggests, it is characterized by sores or rashes on the hands, feet, and inside the mouth.

**2. What are the main symptoms?**

Symptoms of HFMD typically appear in stages:

First Stage (Prodromal Phase): 3-7 days after infection

• Flu-like symptoms: Fever, reduced appetite, sore throat, and a general feeling of being unwell.

Second Stage (Active Phase): 1-2 days after the fever starts

• Painful sores or ulcers inside the mouth, on the tongue, gums, and inside of the cheeks. This often causes children to refuse food, water, and drool more than usual.

• A skin rash or fluid-filled blisters on the palms of the hands, soles of the feet, and between the fingers and toes. The rash can also appear on the buttocks, knees, or elbows.

• Rash Characteristics: The blisters are typically small with a red base and are not itchy. They usually heal without scabbing or leaving scars.

Important Note: Some children, especially adults, may have no fever and only show the rash. However, a small number of cases (particularly in children under 3) can develop rapidly and lead to serious complications.

**3. How does it spread?**

HFMD is highly contagious and spreads through:

• Fecal-oral route: Contact with contaminated food, water, or objects.

• Respiratory droplets: Inhaling droplets from an infected person's coughs or sneezes.

• Direct contact: Contact with an infected person's saliva, mucus, fluid from blisters, or feces (e.g., on unwashed hands or contaminated surfaces like toys).

Patients are most contagious during the first week of illness, but the virus can remain in the stool for several weeks.

**4. How is it treated?**

There is no specific antiviral drug to cure HFMD. Treatment focuses on supportive care to relieve symptoms:

• Reduce Fever: Use fever reducers like acetaminophen or ibuprofen (as directed by a doctor).

• Manage Mouth Pain: Use topical oral anesthetic gels or sprays to numb the pain and ensure the child can drink and eat.

• Hydration: This is crucial. Encourage drinking cool fluids to prevent dehydration caused by painful swallowing.

• Rest: Ensure plenty of rest.

• Skin Care: Keep the skin clean and dry. Avoid breaking the blisters to prevent secondary bacterial infections.

Note: Antibiotics are ineffective against viruses and should not be used.

**5. How can it be prevented?**

Prevention is key. Main measures include:

• Frequent Handwashing: Wash hands thoroughly with soap and running water, especially after using the toilet, before eating, and after touching public surfaces.

• Practice Good Hygiene: Avoid close contact like kissing, hugging, or sharing utensils with infected individuals.

• Disinfect Surfaces: Clean and disinfect frequently touched surfaces and toys.

• Avoid Touching Your Face: Avoid touching your eyes, nose, and mouth with unwashed hands.

• Stay Home if Sick: Keep infected children home from school or daycare to prevent spreading the virus.

**2.What are the typical symptoms of Hand, Foot, and Mouth Disease?**

**Answer：:**

The typical symptoms of Hand, Foot, and Mouth Disease (HFMD) follow a specific pattern and primarily affect the mouth and skin. Here is a breakdown:

1. Fever:

• This is often one of the first signs. A mild to moderate fever is common, usually lasting 1-2 days.

2. Sore Throat and Malaise:

• Children may feel generally unwell, tired, and have a sore throat, which can lead to reduced appetite.

3. Mouth Sores:

• A day or two after the fever starts, painful red spots or blisters appear inside the mouth. These typically develop on the tongue, gums, and inside of the cheeks.

• They quickly turn into small ulcers with a red base. This condition is often called herpangina.

• These mouth sores are the primary cause of pain when swallowing, leading to decreased fluid intake and risk of dehydration.

4. Skin Rash:

• Around the same time as the mouth sores, a non-itchy skin rash appears.

• It is characterized by flat red spots or small blisters.

• The rash has a distinctive distribution:

• On the palms of the hands

• On the soles of the feet

• Sometimes on the buttocks, knees, or elbows.

Key Characteristics of the Rash:

• The blisters are often described as having a grayish center and are surrounded by a red outline.

• Unlike chickenpox, the HFMD rash usually does not itch and does not scar as it heals.

In summary, the classic triad of symptoms that gives the disease its name is: sores in the mouth, and a rash on the hands and feet, preceded by a fever.

**3.How is Hand, Foot, and Mouth Disease transmitted?**

**Answer：：**

Hand, Foot, and Mouth Disease (HFMD) is highly contagious and spreads easily from person to person through several main routes. The viruses that cause HFMD (such as Coxsackievirus) are found in an infected person's bodily fluids.

The primary routes of transmission are:

1. Direct Contact with Infectious Persons:

• This is the most common way the virus spreads.

• Contact with Blister Fluid: The fluid from the skin blisters can contain the virus.

• Contact with Respiratory Secretions: This includes contact with saliva, drool, or mucus from the nose and throat.

2. Respiratory Route:

• The virus can be spread through the air when an infected person coughs or sneezes, releasing tiny droplets containing the virus. Others can inhale these droplets.

3. Fecal-Oral Route:

• The virus is present in the stool of an infected person and can survive there for several weeks after symptoms have gone away.

• Infection can occur if a person touches surfaces contaminated with infected stool (e.g., when changing diapers) and then touches their own mouth, nose, or eyes. This is a key reason why the disease spreads rapidly among young children who are not yet fully toilet-trained.

4. Contact with Contaminated Surfaces and Objects:

• The virus can live on surfaces and objects for days.

• Common sources of infection include touching toys, doorknobs, tables, or utensils that have been contaminated by an infected person, and then touching the face.

Key Points to Remember:

• Individuals are most contagious during the first week of illness.

**• However, the virus can continue to be shed in respiratory secretions for days after symptoms resolve and in stool for several weeks, making good hygiene crucial to prevent ongoing spread.**

**4.What is the course of Hand, Foot, and Mouth Disease?**

**Answer：**

The course of Hand, Foot, and Mouth Disease (HFMD) typically follows a predictable timeline from infection to recovery. Here is a stage-by-stage breakdown of the disease's progression:

Stage 1: Incubation Period (Exposure to First Symptoms)

• Duration: 3 to 7 days.

**• What happens: After a person is exposed to the virus, it multiplies in the body. During this stage, there are no signs or symptoms.**

Stage 2: Onset of Illness (Prodromal Phase)

• Duration: 1 to 2 days.

**• What happens: The initial symptoms begin to appear. These are often non-specific and include:**

◦ Fever (often the first sign)

◦ Sore throat

◦ Reduced appetite

◦ General feeling of being unwell (malaise)

Stage 3: Active Disease Phase

• Duration: Begins a day or two after the fever starts; the full phase typically lasts 3 to 7 days.

**• What happens: The characteristic symptoms develop:**

◦ Painful mouth sores appear, which can develop into ulcers.

◦ The skin rash on the hands, feet, and sometimes other body parts becomes visible.

◦ This is when the disease is most recognizable and often the most uncomfortable period, especially due to painful mouth sores that can make drinking difficult.

Stage 4: Recovery Phase

• Duration: Most people recover fully within 7 to 10 days from the onset of symptoms.

**• What happens: The fever has usually subsided. The mouth sores and skin rash begin to heal. The skin rash and blisters dry up and fade without scarring. The person starts to feel better and regains their appetite.**

Important Note on Contagiousness:

A person is most contagious during the first week of illness. However, the virus can continue to be shed in respiratory secretions for a few weeks and, more notably, in the stool for several weeks or even months after recovery. This is why maintaining good hygiene long after symptoms are gone is crucial to prevent spreading the virus.

**5.Is antibiotics needed for Hand, Foot, and Mouth Disease?**

**Answer：**

No, antibiotics are not effective against Hand, Foot, and Mouth Disease (HFMD) and should not be used to treat it.

Here’s a detailed explanation:

1. Cause of the Disease:

• HFMD is caused by a virus (specifically, enteroviruses).

• Antibiotics are designed to kill bacteria, not viruses. Using them for a viral infection is ineffective and will not help the patient recover faster or reduce symptoms.

2. Standard Treatment:

• Since there is no specific cure for the virus itself, treatment is supportive and focuses on relieving symptoms. This includes:

◦ Using fever reducers and pain relievers (e.g., acetaminophen or ibuprofen) to manage fever and pain from mouth sores.

◦ Ensuring adequate fluid intake to prevent dehydration.

◦ Providing soft, bland foods that are easier to eat with a sore throat.

3. When Might Antibiotics Be Used?

• In rare cases, a doctor might prescribe antibiotics if a secondary bacterial infection develops as a complication of HFMD.

• Examples of such complications include:

◦ Bacterial skin infection from scratching the blisters.

◦ Ear infection.

◦ Pneumonia.

Conclusion:

Do not use antibiotics to treat Hand, Foot, and Mouth Disease. It is crucial to follow a doctor's advice, which will focus on supportive care to help the body fight off the virus naturally. The misuse of antibiotics contributes to the serious global problem of antibiotic resistance.

**6.How should fever be managed in a child with Hand, Foot, and Mouth Disease?**

**Answer：**

Managing fever in a child with Hand, Foot, and Mouth Disease (HFMD) is important for their comfort and to prevent complications like dehydration. Here is a step-by-step guide on how to handle it:

1. Assess the Fever:

• Use a reliable thermometer to check the child's temperature.

• A fever is generally defined as a temperature of 100.4°F (38°C) or higher.

2. Use Fever-Reducing Medication (if needed):

• Purpose: Medication is primarily used to make the child more comfortable, especially if they are irritable, in pain, or refusing fluids. It is not always necessary to treat the fever itself if the child is otherwise comfortable.

• Recommended Medications:

• Acetaminophen (e.g., Tylenol, Panadol, Calpol)

• Ibuprofen (e.g., Advil, Motrin). Note: Ibuprofen should not be used for children under 6 months without doctor's advice.

• Crucial Instructions:

• Always consult a doctor or pharmacist for the correct dosage based on your child's weight and age.

• Never give aspirin to a child with a viral illness, as it is associated with a rare but serious condition called Reye's syndrome.

• Do not use both acetaminophen and ibuprofen at the same time unless specifically instructed by a doctor.

3. Focus on Hydration (This is Critical):

• Fever increases the risk of dehydration, which is further compounded by painful mouth sores that make drinking difficult.

• Offer plenty of cool fluids frequently in small amounts. Cool liquids can also soothe a sore throat.

• Good options include: water, diluted juice, oral rehydration solutions (ORS), ice pops, or gelatin.

• Monitor for signs of dehydration: decreased urination (fewer wet diapers), no tears when crying, dry mouth, sunken eyes, or unusual lethargy. If you see these signs, contact a doctor immediately.

4. Use Comfort Measures:

• Dress the child in lightweight, loose clothing.

• Keep the room temperature cool and comfortable.

• Do not bundle the child up with heavy blankets, as this can trap body heat.

5. Monitor the Child's Overall Condition:

• Watch for any warning signs that require immediate medical attention, even if the fever itself is not very high. These include:

• Severe headache, stiff neck, or sensitivity to light.

• Persistent vomiting.

• Difficulty breathing.

• Lethargy, confusion, or irritability that worsens.

• Signs of dehydration (as mentioned above).

• If the fever persists for more than 2-3 days.

Summary: The key to managing fever in HFMD is to use medication for comfort, prioritize fluid intake above all else, and closely monitor for any signs of serious complications. Always seek professional medical advice for a proper diagnosis and treatment plan.

**7.What are the specific precautions to take during home isolation for Hand, Foot, and Mouth Disease?**

**Answer：**

During home isolation for Hand, Foot, and Mouth Disease (HFMD), the primary goals are to prevent the virus from spreading to others and to help the infected child recover comfortably. Here are the specific precautions to take:

1. Strict Isolation:

• Stay Home: Keep the child home from school, daycare, or any public places.

• Avoid Contact: Avoid playdates, visits to other homes, and public gatherings like parties, shopping malls, or playgrounds.

• Limit Contact within Households: As much as possible, limit the child's close contact with other children and family members (e.g., avoid hugging, kissing). Do not share items.

2. Meticulous Hygiene to Prevent Spread:

• Frequent Handwashing: Everyone in the household should wash their hands often with soap and water, especially after contact with the sick child, changing diapers, or touching any of their belongings.

• Separate Items: Use separate utensils, cups, towels, bedding, and toiletries for the sick child. Wash these items thoroughly after use.

• Avoid Sharing Food: Do not share food or drinks.

3. Thorough Environmental Disinfection:

• Clean and Disinfect Surfaces: Regularly clean and disinfect high-touch surfaces that the child may have contacted. Key areas include:

• Toys

• Doorknobs and light switches

• Tabletops and countertops

• Faucets

• Remote controls and electronic devices

• Use Appropriate Cleaners: Use an EPA-registered disinfectant or a freshly prepared bleach solution. Ensure the disinfectant remains on the surface for the recommended contact time to kill the virus.

4. Symptom Management and Care:

• Comfort Care: Continue managing fever and pain with medications as advised by a doctor.

• Hydration: Closely monitor fluid intake. Offer cool, soft, and bland foods that are easy to swallow (e.g., yogurt, applesauce, smoothies). This is crucial to prevent dehydration.

• Monitor for Complications: Watch for any warning signs that require immediate medical attention, such as:

• Signs of dehydration (e.g., no urine for 4-6 hours, no tears, dry mouth).

• High fever that doesn't respond to medication.

• Lethargy, confusion, or irritability.

• Difficulty breathing.

• Severe headache or stiff neck.

5. Knowing When to End Isolation:

• The child can typically return to school or group settings after all three of the following criteria are met:

1. The fever has been gone for at least 24 hours without using fever-reducing medicine.

2. The mouth sores have healed.

3. The blisters have dried and crusted over.

• Important Note: The virus can still be shed in stool for several weeks, so maintaining strict hygiene, especially handwashing after using the toilet or changing diapers, remains essential even after the child returns to normal activities.

Summary: Successful home isolation relies on a combination of strict separation, rigorous hygiene practices, thorough cleaning, attentive home care, and knowing when it is safe for the child to return to the community.

**8.How can Hand, Foot, and Mouth Disease be prevented?**

**Answer：**

There is no vaccine for Hand, Foot, and Mouth Disease (HFMD) in most countries. Therefore, prevention relies primarily on practicing good hygiene and taking precautions to avoid infection. The key strategies are:

1. Practice Meticulous Hand Hygiene (Most Important Step):

• Wash hands frequently with soap and water for at least 20 seconds, especially:

◦ After using the toilet or changing diapers.

◦ Before eating and preparing food.

◦ After blowing your nose, coughing, or sneezing.

◦ After contact with someone who is sick or their environment.

• If soap and water are not available, use an alcohol-based hand sanitizer. However, soap and water are preferred.

2. Disinfect Surfaces and Objects:

• Regularly clean and disinfect frequently touched surfaces and objects, such as toys, doorknobs, tables, and countertops. The viruses that cause HFMD can live on surfaces for days.

• Use a diluted bleach solution or an EPA-registered disinfectant effective against viruses.

2. Disinfect Surfaces and Objects:

• Regularly clean and disinfect frequently touched surfaces and objects, such as toys, doorknobs, tables, and countertops. The viruses that cause HFMD can live on surfaces for days.

• Use a diluted bleach solution or an EPA-registered disinfectant effective against viruses.

3. Avoid Close Contact:

• Avoid close contact like kissing, hugging, or sharing eating utensils or cups with people who have HFMD.

• Do not share personal items such as towels, washcloths, or toothbrushes.

4. Practice Respiratory Etiquette:

• Cover your mouth and nose with a tissue or your upper sleeve (not your hands) when coughing or sneezing. Throw the tissue in the trash immediately.

• Wash your hands immediately after coughing, sneezing, or blowing your nose.

5. Isolate Infected Individuals:

• Keep children with HFMD home from school, daycare, or other group settings until they are no longer contagious.

• They are considered no longer contagious when all three of these conditions are met:

1. Their fever has gone (for at least 24 hours without fever-reducing medicine).

2. Their mouth sores have healed.

3. Their blisters have dried and scabbed over.

6. Special Precaution for Caregivers:

• Be extra careful when handling items soiled by an infected person, especially diapers and tissues. Wash your hands thoroughly immediately afterwards.

• The virus can be shed in stool for several weeks after recovery, so maintaining good hygiene is crucial long after symptoms disappear.

Summary: Prevention of HFMD centers on breaking the chain of transmission through consistent and thorough handwashing, disinfecting contaminated surfaces, and avoiding close contact with infected individuals during their contagious period.

**9. What are the incubation period and infectious period of hand, foot, and mouth disease?**

**Answer:**

Incubation Period: Typically 3 to 7 days. This is the time from initial infection to the onset of symptoms.

Infectious Period: People are most contagious during the first week of illness. However, the virus can remain in the body and be spread for several weeks after symptoms have gone away, especially through the stool.

**10. How long after being cured of hand, foot, and mouth disease can a child return to kindergarten or school?**

**Answer:**

It is generally recommended that a child should stay home and isolate themselves until they have fully recovered. The key criteria for returning are:

1. Fever-free for at least 24 hours without using fever-reducing medicine.

2. Sores have healed, meaning the blisters (vesicles) on the hands and feet have scabbed over or disappeared.

3. Mouth ulcers have healed, allowing the child to eat and drink normally.

Typically, this isolation period is at least one weekfrom the onset of symptoms. However, the virus can still be shed in stools for several weeks after recovery, so maintaining good hygiene is crucial.

Important: Always follow the specific rules and recommendations of your local health authority, the child's school, or kindergarten, as they may have slightly different protocols. A doctor's note may be required for readmission.

**11. What are the commonly used and effective disinfection methods for hand, foot, and mouth disease?**

**Answer:**

The viruses that cause hand, foot, and mouth disease (mainly Coxsackievirus and Enterovirus 71) are resistant to many common disinfectants but can be effectively killed by the following methods. The key is to focus on disinfecting items and surfaces contaminated with respiratory droplets or feces.

1. Recommended First-Choice Disinfectants:

Chlorine-Based Disinfectants: Such as bleach (sodium hypochlorite). They are the most effective.

Dilution Ratio: For general surface disinfection, use a concentration of 500 mg/L. A common method is to mix 1 part of 5% household bleach with 99 parts of water.

For Heavy Contamination: For surfaces soiled with feces or respiratory secretions, use a stronger concentration of 1000 mg/L (1 part of 5% bleach to 49 parts of water).

2. Other Effective Disinfectants and Methods:

Heat: High temperatures can kill the virus. Methods include:

Boiling:Boil items like utensils, bottles, and towels for20 minutes.

Steam Disinfection: Using a steam sterilizer.

Ultraviolet Light: Direct sunlight (UV radiation) can help disinfect surfaces, bedding, and toys. However, this is less reliable than chemical or heat methods.

Drying:The virus survives poorly in dry environments. Ensuring good ventilation and drying surfaces helps.

3. Ineffective Disinfectants to Avoid:

Alcohol-based disinfectants (like hand sanitizers) are generally not effective against the enteroviruses that cause HFMD. Soap and water are superior for hand hygiene.

Key Areas to Disinfect:

Surfaces:Tables, countertops, door handles, toilet seats, and floors.

Toys:Especially toys that children put in their mouths.

Utensils:Cups, bowls, spoons, and baby bottles.

Textiles: Clothing, bed linens, and towels contaminated with secretions. Wash them with hot water.

Hands: Frequent and thorough handwashing with soap and water is the most crucial preventive measure.

Summary: A diluted bleach solution is the most commonly used and effective chemical disinfectant for surfaces. For heat-resistant items, boiling is a simple and reliable method.

**12. Are there any special concerns for infants under 3 years old who contract the disease?**

**Answer:**

Yes, infants and children under 3 years old require special attention when they contract hand, foot, and mouth disease (HFMD) because they are considered a higher-risk group. Here are the key areas of concern:

Higher Risk of Severe Complications:

Young children have less mature immune systems, making them more susceptible to severe forms of the disease.

They are at a higher risk for serious complications such as viral meningitis, encephalitis (brain inflammation), or acute flaccid paralysis (polio-like paralysis).

EV-A71 infection is a common cause of severe HFMD, and it disproportionately affects this age group.

2. Dehydration is a Primary Concern:

Painful mouth ulcers can make it very difficult and uncomfortable for infants to drink fluids.

Closely monitor for signs of dehydration:

Reduced wet diapers or not urinating for 6-8 hours.

Crying with few or no tears.

Dry mouth and lips.

Sunken eyes or a sunken soft spot (fontanelle) on the head.

Unusual lethargy or irritability.

3. Difficulty in Medication and Feeding:

They cannot verbally express the location or severity of their pain.

Parents may need to use syringe feeding, offer cold, soft, and non-acidic foods (like yogurt, pudding, smoothies), and ensure they get enough fluids.

4. Close Monitoring for Neurological Symptoms:

This is critical. Seek immediate medical attention if the child shows any of these warning signs:

• High Fever: Especially if persistent or exceeding 39°C (102.2°F).

• Startled Easily: Myoclonic jerks or twitching, even when asleep.

• Vomiting

。Breathing Difficulties or rapid breathing.

。Unusual Drowsiness, Lethargy, or Confusion.

。Poor Coordination or weakness in limbs.

Summary: For children under 3, parents should be extra vigilant about hydration, closely monitor for neurological warning signs, and not hesitate to seek prompt medical care if the child's condition worsens or any red flags appear.

**13.Is there a vaccine to prevent hand, foot, and mouth disease? How effective is it?**

**Answer:**

The situation regarding vaccines for hand, foot, and mouth disease (HFMD) is specific:

**1. Is there a vaccine?**

• Yes, but it's targeted. There is currently no universal vaccine that protects against all viruses that cause HFMD.

• China has developed and approved vaccines that specifically target Enterovirus 71 (EV71), which is the most common cause of severe HFMD cases and complications (like brain inflammation and paralysis).

**2. How effective is it?**

• Highly effective against EV71: The EV71 vaccine is very effective at preventing HFMD caused by the EV71 virus. Clinical trials have shown it to be over 90% effective in preventing EV71-associated HFMD.

• Does not protect against other viruses: It is crucial to understand that this vaccine does not protect against HFMD caused by other viruses, such as Coxsackievirus A16 (Cox A16) or other enteroviruses, which are also common causes of the disease.

• Primary benefit: Prevents severe illness. The main goal of the EV71 vaccine is to reduce the rate of severe, potentially life-threatening complications and hospitalizations. Even if a vaccinated child gets HFMD from another virus, they are highly protected from the most dangerous form of the disease caused by EV71.

In summary: The available EV71 vaccine is a powerful tool specifically for preventing the most severe forms of HFMD. However, it is not a complete shield against catching HFMD in general, as other viruses can still cause the illness. Parents should consult their doctor to see if the EV71 vaccine is available and recommended for their child.

**14.What is the cause of hand, foot, and mouth disease?**

**Answer:**

Hand, foot, and mouth disease (HFMD) is caused by viruses that belong to the Enterovirus genus (group) of the Picornaviridae family.

The most common viruses responsible for HFMD are:

• Coxsackievirus A16: This is the most frequent cause of HFMD, typically resulting in a mild form of the illness.

• Enterovirus 71 (EV-A71): This type is often associated with more severe cases of HFMD and a higher risk of serious complications (such as neurological issues).

Several other enteroviruses can also cause the disease.

In short: HFMD is a viral infection, primarily spread through direct contact with an infected person's respiratory droplets, fluid from blisters, or feces (the fecal-oral route).

**15. What is the treatment for hand, foot, and mouth disease?**

**Answer:**

There is no specific antiviral drug or cure that targets the viruses causing hand, foot, and mouth disease (HFMD). Treatment is focused on supportive care to relieve symptoms and prevent complications while the body fights off the virus, which usually takes about 7 to 10 days.

Key aspects of treatment include:

1. Managing Fever and Pain:

◦ Use acetaminophen (e.g., Tylenol) or ibuprofen (e.g., Advil, Motrin) to reduce fever and alleviate pain from mouth sores and skin blisters.

◦ Note: Aspirin should not be given to children due to the risk of Reye's syndrome.

2. Ensuring Adequate Hydration (Most Important):

◦ Mouth sores can make drinking painful, leading to dehydration.

◦ Offer plenty of cold, soothing liquids such as water, milk, or ice pops.

◦ Avoid acidic, spicy, or salty drinks and foods (like orange juice or soda) as they can irritate the sores.

3. Relieving Mouth Pain:

◦ For older children, medicated mouth sprays or numbing lozenges (recommended by a doctor) can help.

◦ For infants, offering a liquid diet (like smoothies, yogurt) using a syringe if necessary can be helpful.

4. Diet and Rest:

◦ Provide soft, bland foods that are easy to swallow.

◦ Ensure the child gets plenty of rest to help their immune system recover.

When to Seek Medical Attention:

Consult a doctor immediately if the child (especially if under 3 years old):

• Shows signs of dehydration (e.g., no urine for 6+ hours, dry mouth, no tears).

• Has a high fever that doesn't respond to medication.

• Appears lethargic, disoriented, or irritable.

• Experiences symptoms like a stiff neck, persistent vomiting, or difficulty breathing.

• Shows neurological warning signs like jerking movements or being easily startled.

Important: Antibiotics are not effective because HFMD is a viral infection.

16. The rash of hand, foot, and mouth disease.

**Answer:**

The rash is a characteristic symptom of hand, foot, and mouth disease (HFMD). Here are its key features:

• Appearance:

◦ It often starts as flat, red spots or macules.

◦ These spots typically develop into small, fluid-filled blisters or vesicles. The blisters are often surrounded by a red halo.

◦ The rash is usually not itchy and, crucially, does not scar after healing.

• Distribution (Where it appears):

◦ Hands: Especially on the palms and between the fingers.

◦ Feet: Especially on the soles and between the toes.

◦ Buttocks and Groin: It is also very common for the rash to appear in these areas.

◦ Knees and Elbows: It can sometimes occur on the knees and elbows.

• Other Key Feature:

◦ Sores or ulcers inside the mouth (on the tongue, gums, and inside of the cheeks) usually accompany the skin rash. These mouth sores can be painful and are often the first sign of the illness.

In summary, the classic HFMD rash is a non-itchy blister-like rash that appears predominantly on the palms, soles, and buttocks, and is accompanied by painful mouth ulcers.

home remedies for hand, foot and mouth disease itching

**Answer：**

Effective Home Remedies for HFMD Itching

1. Cool Compresses

**• How: Apply a clean cloth soaked in cool water to the itchy areas for 15-20 minutes at a time.**

• Why: The cold helps numb the nerve endings and reduce inflammation, providing immediate, temporary relief.

2. Oatmeal Baths

**• How: Add a cup of finely ground colloidal oatmeal (like Aveeno) to a tub of lukewarm (not hot) water. Soak for 15-20 minutes.**

• Why: Oatmeal has anti-inflammatory and soothing properties that can calm irritated skin and relieve itching.

3. Baking Soda Paste or Bath

• As a Paste: Mix a small amount of baking soda with a few drops of water to create a paste. Apply it directly to the blisters and let it dry before gently rinsing off.

• As a Bath: Add half a cup to a full cup of baking soda to a lukewarm bath and soak.

• Why: Baking soda can help neutralize skin pH and has a soothing, anti-itch effect.

4. Hydrating Lotions

**• How: Apply a gentle, fragrance-free lotion or cream (like those containing calamine) to the dry, itchy spots. Calamine lotion is particularly effective as it has a cooling effect and relieves itching.**

• Why: Keeps the skin hydrated, which can reduce the urge to scratch. Avoid lotions with alcohol or heavy fragrances, as they can be irritating.

5. Keep Skin Cool and Dry

**• How: Dress the child in loose, soft, 100% cotton clothing. Avoid overdressing and keep the environment cool.**

• Why: Heat and sweat can significantly worsen itching. Cotton is breathable and less irritating than synthetic fabrics.

6. Manage Pain to Reduce Itching Sensation

**• How: As per your doctor's advice, using age-appropriate doses of acetaminophen (Tylenol) or ibuprofen (Advil, Motrin) can help. By reducing the overall pain and discomfort from the sores, the perception of itching can also lessen.**

**What to AVOID:**

• SCRATCHING: This is the most important rule. Scratching can break the blisters, leading to a secondary bacterial infection and potential scarring. Keep your child's fingernails short and clean. Consider having them wear mittens or socks on their hands at night.

• Harsh Soaps and Bubble Baths: These can strip the skin of its natural oils and worsen irritation.

• Hot Water: Hot baths or showers can increase blood flow to the skin and make itching more intense. Always use lukewarm water.

When to See a Doctor for the Rash:

Consult a doctor if:

• The itching is severe and not relieved by home care.

• The blisters show signs of infection (e.g., increased redness, swelling, pus, or warmth).

• The child has a fever along with a worsening rash.

In summary, the goal is to soothe, cool, and protect the skin while preventing scratching. Oatmeal baths, cool compresses, and calamine lotion are the most reliable and safe starting points.

18.Monitoring the condition of severe hand, foot, and mouth disease.

Monitoring for severe hand, foot, and mouth disease (HFMD) is crucial, especially in children under 3 years old, as the illness can progress rapidly to serious complications like brainstem encephalitis, neurogenic pulmonary edema, and acute flaccid paralysis.

The key is to watch for the following critical warning signs that require immediate medical attention:

1. Neurological Symptoms (Most Critical):

• Frequent Myoclonic Jerks or "Startles": Jerky movements of the limbs, especially during sleep. This is a major red flag.

• Irritability and Crying: Child is unusually fussy, irritable, or cries incessantly and cannot be soothed.

• Lethargy and Weakness: Child is unusually drowsy, sleepy, difficult to wake, limp, or weak.

• Headache and Vomiting: Complains of severe headache or has frequent, projectile vomiting.

• Ataxia (Poor Coordination): Staggering when walking, clumsiness, or limb tremors.

• Stiff Neck or Photophobia (sensitivity to light): Signs that may suggest meningitis.

2. Cardiorespiratory Symptoms (Signs of Heart and Lung Involvement):

• Rapid Heart Rate (Tachycardia): Faster heart rate than normal for the child's age and activity level.

• Rapid, Labored, or Irregular Breathing (Dyspnea): Grunting, chest retractions, or bluish discoloration of the lips or skin (cyanosis). This is a medical emergency.

• Cold, Clammy Extremities: Cold hands and feet despite having a fever.

• High Blood Pressure (Hypertension): This can be a sign of autonomic nervous system dysfunction.

3. Systemic Symptoms:

• Persistent High Fever: A fever that is difficult to control with medication, especially if it lasts for more than 48-72 hours.

• Poor Perfusion: Delayed capillary refill time (press on a fingernail; it should return to pink in less than 2 seconds).

• Decreased Urination: A sign of dehydration or poor circulation.

Summary: Parents and caregivers must be vigilant. If a child with HFMD shows any of the above symptoms—particularly frequent startles, lethargy, breathing difficulties, or poor circulation—they must be taken to a hospital immediately. Close monitoring in the first week of illness, especially days 2-5, is essential.

**19.What should be noted for a child with hand, foot, and mouth disease receiving home care?**

**Answer:**

Caring for a child with HFMD at home focuses on relieving symptoms, preventing the spread of the virus, and monitoring for complications. Here are the key points to note:

1. Isolation is Crucial

• Stay Home: Keep the child away from kindergarten, school, and public places to prevent outbreaks.

• Limit Contact: Avoid contact with other children, especially infants and pregnant women, within the same household as much as possible.

• Duration: Isolate until the fever has subsided, the rash has crusted over, and the mouth ulcers have healed (typically at least one week).

2. Symptom Management

• Fever and Pain: Use pediatric formulations of acetaminophen or ibuprofen as directed to manage fever and pain from mouth sores.

• Mouth Care:

◦ Offer cold, soft, bland foods like yogurt, pudding, applesauce, and smoothies. Avoid acidic, spicy, or salty foods.

◦ Encourage frequent sips of cool water. Using a straw can help bypass painful mouth sores.

• Skin Care (Rash):

◦ Keep the skin clean and dry.

◦ To relieve itching, use cool compresses or calamine lotion.

◦ Prevent Scratching: Keep the child's fingernails short and clean to prevent breaking the blisters and causing a secondary bacterial infection.

3. Prevent Dehydration (Top Priority)

• Closely monitor for signs of dehydration: dry mouth, no tears when crying, sunken eyes, and significantly decreased urination (e.g., no wet diaper for 6-8 hours).

• Encourage fluid intake frequently. Oral rehydration solutions are excellent for preventing dehydration.

4. Strict Hygiene to Prevent Spread

• Frequent Handwashing: Everyone in the household should wash hands frequently with soap and water, especially after contact with the child.

• Disinfection: Regularly clean and disinfect surfaces, toys, and commonly touched objects with a diluted bleach solution. The child's utensils, towels, and bedding should be separate and washed thoroughly.

• Waste Disposal: Properly dispose of tissues and diapers contaminated with respiratory secretions or feces.

5. Close Monitoring for Severe Symptoms

Be vigilant and seek immediate medical attention if the child shows any of these red flags:

• Neurological Signs: Frequent startles (myoclonic jerks), vomiting, drowsiness, lethargy, or severe headache.

• Respiratory/Cardiac Signs: Rapid breathing, difficulty breathing, or a fast heart rate.

• Dehydration: Signs as mentioned above.

• High Fever: A persistent high fever that is not controlled by medication.

Summary: The core of home care is providing comfort, ensuring hydration, maintaining strict hygiene to protect others, and watching closely for any warning signs of a severe infection.

20.Who is susceptible to hand, foot, and mouth disease?

**Answer:**

Hand, foot, and mouth disease (HFMD) primarily affects infants and children under 5 years old. This group is the most susceptible.

The main reasons are:

• Lack of Prior Immunity: Young children have not been exposed to the viruses that cause HFMD before and therefore have no existing immunity.

• Hygiene Habits: They are more likely to put their hands and objects in their mouths, facilitating the spread of the virus.

**However, it is possible for older children and adults to get infected, especially if they have a weakened immune system or have never been exposed to the specific virus strain before. Infections in older age groups are often milder or may even show no symptoms.**

In summary, the most susceptible population is young children, particularly those in childcare settings like kindergartens and preschools, where the virus can spread easily.

## **Gemini**

**What is Hand, Foot, and Mouth Disease (HFMD)？**

**ANSWER:**

Of course. Here is a detailed explanation of Hand, Foot, and Mouth Disease (HFMD).

**1. What is Hand, Foot, and Mouth Disease (HFMD)?**

Hand, Foot, and Mouth Disease is a common and highly contagious viral illness that primarily affects infants and young children, typically under the age of 5. However, older children and adults can also contract it.

The disease is characterized by a distinctive rash or blisters on the hands, feet, and in the mouth, which gives it its name. Despite its scary-sounding name, HFMD is usually a mild and self-limiting illness, with most people recovering on their own within 7 to 10 days.

It is important to note that HFMD is not related to foot-and-mouth disease (also known as hoof-and-mouth disease), which is an illness that affects cattle, sheep, and pigs.

**2. What Causes HFMD?**

HFMD is caused by a group of viruses known as enteroviruses. The most common culprits are:

Coxsackievirus A16: This is the most frequent cause and usually results in a mild form of the disease.

Enterovirus 71 (EV71): This strain can also cause HFMD and has a higher risk of leading to serious complications, such as viral meningitis or encephalitis (brain inflammation), although such complications are rare.

Other Coxsackieviruses (like A6): Some other strains can cause more atypical rashes, sometimes more widespread and severe.

**3. What are the Symptoms?**

Symptoms usually appear 3 to 6 days after exposure to the virus (the incubation period).

Initial Symptoms (First 1-2 days):

Fever

Sore throat

Reduced appetite

A general feeling of being unwell (malaise)

Later Symptoms (After the fever begins):

Mouth Sores: Painful red spots that blister and can become ulcers. They typically appear on the tongue, gums, and inside of the cheeks. These sores can make eating, drinking, and swallowing painful.

Skin Rash: A non-itchy rash with flat or raised red spots, some with blisters. This rash is characteristically found on the palms of the hands and the soles of the feet.

Other Areas: The rash can also appear on the buttocks, knees, elbows, and genital area.

Not everyone will experience all of these symptoms. Some people, especially adults, may become infected and show no symptoms at all but can still spread the virus to others.

**4. How Does HFMD Spread?**

HFMD is very contagious. The virus can be found in an infected person's:

Nose and throat secretions (saliva, sputum, or nasal mucus)

Fluid from blisters

Stool (feces)

It is spread through:

Direct contact with an infected person (kissing, hugging, or sharing cups/utensils).

Contact with blister fluid or stool. This is a major route of transmission, especially in toddlers during diaper changes.

Respiratory droplets released into the air when an infected person coughs or sneezes.

Touching contaminated objects and surfaces, then touching your eyes, nose, or mouth.

A person is most contagious during the first week of their illness. The virus can remain in the stool for several weeks after symptoms have resolved, meaning a person can still be contagious even after they feel better.

**5. How is HFMD Treated?**

There is no specific medical treatment or vaccine for HFMD. Since it is a viral infection, antibiotics are not effective.

Treatment focuses on relieving symptoms:

Pain and Fever: Over-the-counter pain relievers like acetaminophen (Tylenol) or ibuprofen (Advil, Motrin) can help reduce fever and ease the pain from mouth sores. Do not give aspirin to children, as it has been linked to a rare but serious condition called Reye's syndrome.

Hydration: It is crucial to drink plenty of fluids to prevent dehydration, as mouth sores can make swallowing painful. Cold liquids, milk-based drinks, ice pops, or sherbet can be soothing. Avoid acidic or spicy foods and drinks (like citrus juice or soda) that can irritate mouth sores.

Mouth Rinses: Using a salt-water rinse (1/2 teaspoon of salt in a glass of warm water) can help soothe mouth sores in older children and adults.

**6. How Can HFMD Be Prevented?**

Good hygiene is the best defense against HFMD.

Wash Hands Frequently: Wash hands thoroughly with soap and water for at least 20 seconds, especially after changing diapers, using the toilet, and before preparing or eating food.

Clean and Disinfect: Regularly clean and disinfect frequently touched surfaces and shared items (toys, doorknobs).

Avoid Close Contact: Avoid kissing, hugging, or sharing eating utensils with people who have HFMD.

Teach Good Hygiene: Encourage children to wash their hands and to cover their mouths and noses when coughing or sneezing.

Isolate the Sick: Keep children with HFMD out of school or daycare until their fever is gone and their sores have healed to prevent spreading the virus.

When to See a Doctor

While HFMD is usually mild, you should contact a healthcare provider if:

The infected person is not drinking enough fluids and is showing signs of dehydration (dry mouth, no tears, fewer wet diapers).

Symptoms are severe or do not improve after 10 days.

The child has a weakened immune system.

The child is younger than 6 months old.

The child develops a high fever, stiff neck, headache, or seems unusually sleepy or confused, as these could be signs of rare but serious complications.

**2.What are the typical symptoms of Hand, Foot, and Mouth Disease?**

**Answer：:**

Of course. The symptoms of Hand, Foot, and Mouth Disease (HFMD) typically appear in stages, usually starting 3 to 6 days after infection. Here is a breakdown of the typical symptoms and their progression.

Initial Stage (Prodromal Symptoms)

The first signs of HFMD are often similar to a common cold or flu and usually last for 1 to 2 days. They include:

Fever: This is often the first symptom to appear. It's usually low-grade but can sometimes be high.

Sore Throat: The throat may be red and painful.

Reduced Appetite: Due to feeling unwell and the developing sore throat.

Malaise: A general feeling of being tired, unwell, or "out of sorts."

Characteristic Stage (The Rash and Sores)

About one or two days after the fever begins, the more distinctive symptoms of HFMD start to develop.

1. Mouth Sores (Herpangina)

This is often the most uncomfortable symptom, especially for young children.

Appearance: They begin as small, bright red spots, which quickly turn into painful blisters and then open ulcers.

Location: They are typically found on the tongue, gums, and inside of the cheeks. They can also appear on the roof of the mouth and in the back of the throat.

Effect: These sores can make it painful to eat, drink, or swallow, leading to irritability and a risk of dehydration.

2. Skin Rash

This is the most visible sign of the disease.

Appearance: The rash consists of flat or raised red spots, and some may develop into blisters (vesicles) with a grayish center. A key feature is that the rash is typically not itchy.

Classic Locations: As the name suggests, the rash is most commonly found on:

The palms of the hands

The soles of the feet

Other Common Locations: The rash can also appear on:

The buttocks (this is very common in infants and toddlers)

Knees and elbows

The genital area

Summary of Typical Symptoms

To put it all together, a typical case of HFMD follows this pattern:

Start: Begins with a fever and a general feeling of being sick (malaise, sore throat).

Followed by: Painful sores inside the mouth.

Then: A non-itchy rash or blisters appear on the hands, feet, and often the buttocks.

It's important to know that not everyone will have all these symptoms. Some children might only have the mouth sores, while others might only have the skin rash. Adults, in particular, may have very mild symptoms or no symptoms at all, but they can still be contagious.

**3.How is Hand, Foot, and Mouth Disease transmitted?**

**Answer：**

Of course. Hand, Foot, and Mouth Disease (HFMD) is highly contagious, which is why it spreads so easily in places like schools and daycare centers. The virus is transmitted from an infected person to others through several common routes.

First, it's helpful to know where the virus is found in an infected person. The virus can be present in their:

Respiratory secretions (saliva, sputum, or nasal mucus)

Fluid from the blisters

Stool (feces)

Based on this, here are the primary ways HFMD is transmitted:

1. Person-to-Person Contact (Direct Contact)

This is the most common way the disease spreads.

Touching an infected person: This includes hugging or shaking hands.

Contact with blister fluid: If you touch the rash or blisters of someone with HFMD, you can get the virus on your hands.

Kissing: Sharing saliva through kissing can easily transmit the virus.

2. The Fecal-Oral Route

This route is especially common among young children who are not yet toilet-trained.

Changing Diapers: An infected person's stool contains the virus. If you change the diaper of an infected child and don't wash your hands thoroughly afterward, you can easily spread the virus to yourself or others by touching your mouth, nose, or eyes.

Contaminated Food or Water: Though less common, if someone who is infected doesn't wash their hands properly after using the toilet and then prepares food, they can contaminate it.

3. Respiratory Droplets (Airborne Transmission)

The virus can become airborne when an infected person talks, coughs, or sneezes.

Inhaling Droplets: People nearby can breathe in these small droplets, leading to infection.

4. Contact with Contaminated Objects and Surfaces (Indirect Contact)

The enteroviruses that cause HFMD can survive on surfaces for several hours.

Touching Contaminated Items: You can get infected if you touch objects and surfaces that have the virus on them (like toys, doorknobs, or tabletops) and then touch your own eyes, nose, or mouth before washing your hands.

Important Points About Transmission

High Contagion Period: People with HFMD are most contagious during the first week of their illness.

Lingering Virus: The virus can remain in the stool for weeks or even months after symptoms have disappeared. This means a person can still be contagious and spread the virus even after they feel completely better.

Asymptomatic Spread: Some people, especially adults, can be infected with the virus and spread it to others without ever showing any symptoms themselves.

Due to these factors, practicing excellent hand hygiene is the single most effective way to prevent the spread of Hand, Foot, and Mouth Disease.

**4.What is the course of Hand, Foot, and Mouth Disease?**

**Answer：**

Of course. The "course" of an illness refers to its typical timeline and progression from infection to recovery. For Hand, Foot, and Mouth Disease (HFMD), the course is generally predictable and mild.

Here is a step-by-step breakdown of the typical course of HFMD:

Stage 1: Incubation Period

Timeline: 3 to 6 days

**What's Happening: This is the period after you are exposed to the virus but before any symptoms appear. The virus is multiplying in your body, but you feel completely normal. You are generally not contagious during this initial phase.**

Stage 2: Prodromal Phase (Initial Symptoms)

Timeline: 1 to 2 days

**What's Happening: The first signs of illness appear. The body begins its immune response.**

Symptoms:

Fever: Often the very first sign. It's usually low-grade but can sometimes be higher.

Sore Throat: Pain and redness in the throat.

Malaise: A general feeling of being unwell, tired, and irritable.

Reduced Appetite: The child may not want to eat or drink as much.

Contagiousness: The person starts to become contagious during this phase as the virus is present in their throat and respiratory secretions.

Stage 3: Acute Phase (Characteristic Rash and Sores)

Timeline: 3 to 7 days

**What's Happening: This is when the classic, tell-tale symptoms of HFMD appear, usually 1 to 2 days after the fever started.**

Symptoms:

Mouth Sores (Herpangina): Painful red spots appear inside the mouth (on the tongue, gums, inner cheeks) and quickly develop into blisters and then ulcers. This is often the most painful part of the illness and can make eating and drinking difficult.

Skin Rash: A non-itchy rash appears on the body. It consists of flat or raised red spots, some of which may turn into fluid-filled blisters. The rash is most common on the palms of the hands, soles of the feet, and often on the buttocks.

Contagiousness: This is the peak period of contagiousness. The virus is present in saliva, fluid from the blisters, and stool.

Stage 4: Recovery Phase

Timeline: Symptoms usually resolve within 7 to 10 days from the start of the illness.

**What's Happening: The body's immune system has successfully fought off the virus.**

Progression:

The fever subsides.

The mouth sores heal.

The blisters on the hands and feet dry up and the rash fades without scarring.

Contagiousness: While the person feels better and is less contagious through saliva and blister fluid, the virus can persist in their stool for several weeks or even months. This means they can still spread the virus, which is why continued good hand hygiene is critical.

Possible Late-Stage Symptom (Less Common)

Timeline: 3 to 6 weeks after the illness has resolved.

**What's Happening: In some cases, particularly with certain viral strains (like Coxsackievirus A6), a person may experience peeling skin on their fingers and toes, or even loss of fingernails or toenails.**

Symptoms:

Skin Peeling: Painless peeling of the skin on the palms and soles where the rash was.

Nail Shedding (Onychomadesis): The nails may develop a ridge and then painlessly fall off. New nails will grow back in their place.

Note: While this can be alarming for parents, it is a temporary and harmless after-effect.

Summary of the Course

Overall, HFMD is a self-limiting illness, meaning it resolves on its own without specific medical treatment in the vast majority of cases.

**5.Is antibiotics needed for Hand, Foot, and Mouth Disease?**

**Answer：**

That is an excellent and very important question.

The short and direct answer is: No, antibiotics are not needed or effective for treating Hand, Foot, and Mouth Disease (HFMD).

Here is a detailed explanation why:

1. HFMD is Caused by a Virus, Not Bacteria

Viruses vs. Bacteria: Hand, Foot, and Mouth Disease is caused by a group of viruses called enteroviruses (most commonly Coxsackievirus). Antibiotics are powerful medicines specifically designed to kill or inhibit the growth of bacteria.

No Effect on Viruses: Antibiotics have absolutely no effect on viruses. Taking them for a viral illness like HFMD, the common cold, or the flu will not cure the infection, speed up recovery, or prevent you from being contagious.

2. Why Taking Unnecessary Antibiotics is Harmful

Using antibiotics when they are not needed is not just ineffective; it can also be harmful.

Risk of Side Effects: Antibiotics can cause side effects like stomach upset, diarrhea, rashes, and allergic reactions.

Promotes Antibiotic Resistance: This is a major public health concern. When you use antibiotics unnecessarily, the bacteria living in your body can be exposed to the drug. The stronger bacteria can survive and multiply, leading to the development of "superbugs" that are resistant to treatment. This makes future bacterial infections much harder to treat for you and for the community.

3. The One Exception: Secondary Bacterial Infection

While HFMD itself does not require antibiotics, there is a rare situation where they might be prescribed.

**What it is: A secondary bacterial infection occurs when bacteria infect the open sores or blisters caused by HFMD. This can happen if a child scratches the blisters with unclean hands, introducing bacteria into the broken skin.**

Signs of a Secondary Infection: A doctor might suspect a secondary bacterial infection if the sores:

Become increasingly red, swollen, and painful.

Start to ooze pus (a thick, yellow or green fluid).

Develop a honey-colored crust.

The child develops a new or returning fever after the initial fever has gone away.

Action: If a doctor diagnoses a secondary bacterial infection, they will then prescribe an antibiotic to treat that specific bacterial infection, not the underlying HFMD virus. This might be a topical antibiotic cream or an oral antibiotic.

Summary

In conclusion, you should never use antibiotics to treat a standard case of Hand, Foot, and Mouth Disease. Treatment should focus on providing comfort and relief from symptoms like fever and pain while the viral illness runs its natural, self-limiting course.

**6.How should fever be managed in a child with Hand, Foot, and Mouth Disease?**

**Answer：**

Of course. Managing the fever is one of the most important parts of caring for a child with Hand, Foot, and Mouth Disease, as it helps keep them comfortable while their body fights the virus.

The primary goal is not to eliminate the fever completely, but to reduce discomfort. Fever is a natural and helpful part of the body's immune response.

Here’s how to safely manage a fever in a child with HFMD:

1. Use Fever-Reducing Medications Correctly

This is the most effective way to lower a fever and relieve pain.

Acetaminophen (Paracetamol):

Brand names include Tylenol®, Panadol®, and others.

This can be given to children of all ages (including infants), but always consult your doctor for infants under 3 months.

Ibuprofen:

Brand names include Advil®, Motrin®, and others.

This should only be given to children who are 6 months of age or older.

Ibuprofen can be particularly helpful because it also reduces inflammation, which may help with the pain from mouth sores.

Crucial Guidelines for Medication:

Dose by Weight, Not Age: Always dose the medication based on your child's current weight. The instructions on the package will provide a weight-based chart. If you are unsure, ask your doctor or pharmacist.

Follow Dosing Intervals: Pay close attention to the timing. Do not give a dose sooner than recommended (e.g., typically every 4-6 hours for acetaminophen, every 6-8 hours for ibuprofen).

Keep a Log: It can be helpful to write down the time and dose of each medication you give to avoid accidental overdosing.

!! CRITICAL SAFETY WARNING: NEVER GIVE ASPIRIN !!

Never give aspirin to a child or teenager who has a viral illness. It is linked to a rare but very serious and potentially fatal condition called Reye's syndrome, which can cause liver and brain damage.

2. Non-Medication Methods for Comfort

These methods can be used alongside medication to help your child feel better.

Encourage Fluids (Crucial for HFMD):

Fever increases fluid loss, and the painful mouth sores from HFMD make children reluctant to drink. This combination creates a high risk for dehydration.

Offer cool liquids, which can also soothe a sore mouth. Good choices include:

Water

Milk

Ice pops or frozen fruit bars

Sherbet or ice cream

Avoid acidic drinks like citrus juice or soda, as they will sting the mouth sores.

Dress Them Lightly:

Dress your child in a single layer of light, breathable clothing (like cotton).

Over-bundling or using heavy blankets can trap heat and make the fever worse.

Give a Lukewarm Bath:

A bath in lukewarm (not cold) water can be soothing and help bring their temperature down as the water evaporates from their skin.

Do not use cold water, ice, or alcohol rubs. Cold water can cause shivering, which actually raises the body's core temperature. Alcohol can be absorbed through the skin and is dangerous.

Ensure Rest:

Encourage quiet activities and plenty of rest. The body needs energy to fight the infection.

3. When to Call a Doctor

While most fevers with HFMD are manageable at home, you should contact your pediatrician immediately if:

Your child is an infant less than 3 months old and has a rectal temperature of 100.4°F (38°C) or higher.

The fever is very high (e.g., above 104°F or 40°C) at any age.

The fever lasts for more than 3 days.

Your child shows signs of dehydration:

Dry mouth or tongue

No tears when crying

Fewer than 4-6 wet diapers in 24 hours (for infants)

Sunken eyes or a sunken soft spot on an infant's head

Your child seems unusually irritable, drowsy, is difficult to wake up, or seems confused.

Your child develops a stiff neck, severe headache, or has trouble breathing.

**7.What are the specific precautions to take during home isolation for Hand, Foot, and Mouth Disease?**

**Answer：**

Of course. Taking specific precautions during home isolation for Hand, Foot, and Mouth Disease (HFMD) is crucial to prevent the virus from spreading to other family members and the community. Since the virus is highly contagious and spreads in multiple ways, a multi-layered approach is most effective.

Here are the specific precautions to take, broken down into key areas:

1. Focus on Excellent Hygiene (The Most Critical Step)

The virus spreads through contact, so breaking that chain is paramount.

Frequent and Thorough Handwashing: This is the single most important precaution.

When: Wash hands immediately after touching the infected person, changing their diaper, touching their rash/blisters, or handling their used tissues. Also, wash before preparing food, before eating, and after using the toilet.

**How: Use soap and warm water and scrub for at least 20 seconds (the time it takes to sing "Happy Birthday" twice). Be sure to clean under the nails and between the fingers.**

Alternative: If soap and water are not available, use an alcohol-based hand sanitizer with at least 60% alcohol.

Avoid Touching Your Face: Make a conscious effort to avoid touching your eyes, nose, and mouth, as this is the primary way the virus enters your body from your hands.

2. Manage the Infected Person and Their Items

The goal is to contain the virus to the infected individual as much as possible.

Isolate the Child: Keep the infected child home from school, daycare, and other public places (like playgrounds or parties) until they are no longer contagious. Your doctor or local public health unit can advise when it's safe to return, but it's typically after the fever is gone and the mouth sores have healed.

Do Not Share Personal Items: The infected person should have their own set of items that are not shared with anyone. This includes:

Towels and washcloths

Eating utensils (forks, spoons), cups, and plates

Toothbrushes

Bedding and pillows

Discourage Close Physical Contact: As difficult as it is with a sick child, try to limit hugging and kissing to reduce the spread through saliva and direct contact.

3. Rigorous Cleaning and Disinfection

The virus can live on surfaces for several hours, making environmental cleaning essential.

Clean First, Then Disinfect: First, clean surfaces with soap and water to remove dirt and germs. Then, apply a disinfectant.

Choose the Right Disinfectant: The enteroviruses that cause HFMD are hardy.

Best: A diluted bleach solution (mix 1 tablespoon of bleach in 4 cups of water). Make a fresh solution daily.

Alternative: EPA-registered disinfecting wipes or sprays that are labeled as effective against viruses like Poliovirus, Coxsackievirus, or Norovirus (check the product label). A standard household disinfectant may be sufficient if it lists virucidal properties.

Focus on High-Touch Surfaces: Regularly disinfect items and surfaces that are touched often, such as:

Doorknobs and light switches

Toys (especially plastic ones that can be wiped down)

Tabletops and countertops

Faucet handles

Remote controls

Potty chairs and toilet seats

Laundry: Wash the infected person's bedding, towels, and clothing in hot water if possible and dry on a high heat setting. Do not "hug" dirty laundry to your chest when carrying it to the machine.

4. Special Precautions for Diapering

The virus is shed in stool for weeks, making diaper changes a high-risk activity.

Create a Designated Changing Area: If possible, change diapers in one specific spot.

Dispose of Diapers Properly: Place soiled diapers in a sealed bag before throwing them in the trash.

Disinfect the Changing Mat: Wipe down the diaper changing surface with a disinfectant after every use.

Wash Hands Immediately: This is critical. Wash your hands thoroughly (and the child's hands, if possible) after every diaper change.

5. When to End Isolation

General Guideline: A child can typically return to school or daycare when:

They have been fever-free for at least 24 hours (without using fever-reducing medication).

Their mouth sores have healed.

The blisters on their hands and feet have started to dry up and scab over.

Check Local Policy: Some schools or public health departments may have slightly different rules, so it's always best to check with them.

Remember Lingering Contagiousness: Even after returning to normal activities, remember that the virus can still be shed in the stool for several weeks. Therefore, strict handwashing, especially after using the toilet or changing diapers, must continue long after the symptoms are gone.

**8.How can Hand, Foot, and Mouth Disease be prevented?**

**Answer：**

Of course. Preventing Hand, Foot, and Mouth Disease (HFMD) is all about breaking the chain of transmission. Since it's a highly contagious virus with no vaccine available, prevention relies entirely on practicing excellent hygiene and taking specific environmental precautions, especially in settings with young children.

Here are the most effective ways to prevent the spread of HFMD:

1. Practice Excellent Hand Hygiene (The #1 Method)

This is the single most important preventive measure. The virus is often spread by hands that have come into contact with infectious material.

Wash Hands Frequently and Thoroughly: Use soap and water and scrub for at least 20 seconds. Teach children to do the same. Key times to wash hands include:

After using the toilet.

After changing a diaper (this is critical, as the virus is in stool for weeks).

After touching blisters or rash.

After coughing, sneezing, or blowing your nose.

Before preparing or eating food.

After returning home from public places, school, or daycare.

Use Hand Sanitizer: If soap and water aren't available, an alcohol-based hand sanitizer (with at least 60% alcohol) is a good alternative, but washing with soap and water is superior for removing the enteroviruses that cause HFMD.

2. Clean and Disinfect High-Touch Surfaces

The viruses that cause HFMD can survive on objects and surfaces for several hours.

Identify High-Touch Areas: Regularly clean and disinfect frequently touched surfaces, especially if someone in the house or community is sick. This includes:

Toys

Doorknobs

Tabletops

Faucet handles

Remote controls

Potty chairs

Use an Effective Disinfectant: Clean surfaces first with soap and water, then use a disinfectant. A diluted solution of chlorine bleach (1 tablespoon of bleach mixed with 4 cups of water) is very effective. You can also use EPA-registered disinfecting wipes or sprays that are effective against viruses like Norovirus or Poliovirus (as these are similar to the viruses that cause HFMD).

3. Avoid Close Contact and Sharing

Reducing direct exposure to an infected person is key to stopping the spread.

Isolate Infected Individuals: Keep children with HFMD home from school, daycare, and other group settings until they are no longer contagious (typically when their fever is gone and blisters have healed).

Avoid Sharing Personal Items: Do not share items that can easily pass saliva or other bodily fluids. This includes:

Cups and water bottles

Eating utensils

Towels

Toothbrushes

Limit Physical Contact: Avoid hugging, kissing, or other close contact with someone who has HFMD.

4. Teach and Reinforce Good Personal Habits

Educating children about hygiene is a proactive way to keep them safe.

Cover Coughs and Sneezes: Teach children to cough or sneeze into a tissue or their elbow, not their hands.

Keep Hands Away from the Face: Encourage children (and adults) to avoid touching their eyes, nose, and mouth, as this is how the virus enters the body.

Don't Put Objects in the Mouth: Teach young children not to put hands, toys, or other objects in their mouths.

Summary of Prevention Strategies:

By consistently applying these hygiene and sanitation practices, you can significantly reduce the risk of contracting and spreading Hand, Foot, and Mouth Disease.

**9. What are the incubation period and infectious period of hand, foot, and mouth disease?**

**Answer:**

Excellent question. Understanding the difference between the incubation period and the infectious period is essential for knowing when someone is at risk of getting sick and when they are capable of spreading the illness.

Here is a breakdown of both for Hand, Foot, and Mouth Disease (HFMD):

1. Incubation Period

The incubation period is the time from when a person is first exposed to the virus until they begin to show the first signs of illness.

Typical Duration: 3 to 6 days.

**What Happens During This Time: After the virus enters the body, it begins to replicate, but the person feels completely normal and has no symptoms.**

Contagiousness: A person is generally not contagious during most of this period. They may start to become contagious in the last day or so of the incubation period, just before symptoms like fever appear.

2. Infectious Period (Period of Contagiousness)

The infectious period is the window of time during which an infected person can pass the virus on to others. For HFMD, this period is quite long and occurs in stages.

When It Begins: A person can start spreading the virus a few days before their symptoms even start.

Peak Contagiousness: The infected person is most contagious during the first week of their illness, when they have active symptoms like fever, mouth sores, and a rash with blisters.

**How the Virus Spreads:**

Through Respiratory Droplets: From saliva, nasal mucus (snot), or coughing and sneezing. This is a primary way it spreads during the early, acute phase.

Through Direct Contact: From the fluid inside the blisters. Touching a blister and then touching your mouth, nose, or eyes can transmit the virus.

Through Stool (Feces): This is a very important and often overlooked route of transmission. The virus can be shed in the stool for several weeks, and sometimes even months, after the symptoms have completely disappeared.

Summary of the Timeline

Here's a simple way to visualize the timeline for a typical case:

In short:

Incubation Period: 3 to 6 days (you feel fine).

Infectious Period: Starts just before symptoms appear, is highest during the first week of illness, and can continue for weeks via stool even after you feel better.

**10. How long after being cured of hand, foot, and mouth disease can a child return to kindergarten or school?**

**Answer:**

That is a very practical and important question that almost every parent of a child with Hand, Foot, and Mouth Disease (HFMD) will ask.

The answer has two parts: the general medical guideline and the specific policy of your child's school or daycare.

The General Medical Guideline

Most doctors and public health organizations agree on a set of criteria to determine when it's safe for a child to return. A child is generally considered ready to go back to kindergarten or school when they meet ALL of the following conditions:

Fever-Free for 24 Hours: The child's fever must be gone for at least 24 hours without the use of any fever-reducing medications (like acetaminophen or ibuprofen). This shows that the body's inflammatory response has subsided.

Mouth Sores Have Healed: The child should be able to eat and drink comfortably. If they are still drooling excessively because of painful mouth sores, they can easily spread the virus through their saliva and are likely not feeling well enough for school.

Blisters Have Dried Up: The blisters on the hands, feet, or other parts of the body should be dried and have started to scab over. While the fluid in the blisters is contagious, dry, crusted-over sores are much less likely to spread the virus. Open, weeping blisters are a sign that the child should remain at home.

In short, the child should be feeling well, have no fever, and have no open or weeping sores.

The Important Caveat: Lingering Contagiousness

It is crucial to understand that even after a child meets these criteria and returns to school, they are not 100% non-contagious.

Shedding in Stool: The viruses that cause HFMD can continue to be shed in the child's stool (feces) for several weeks to months after all symptoms have disappeared.

**What This Means: This is why HFMD can spread so rapidly in daycare and kindergarten settings. A child who appears perfectly healthy can still pass the virus during diaper changes or if they don't wash their hands properly after using the toilet.**

This lingering contagiousness is why it is not practical to keep a child home for weeks on end. Instead, the focus shifts from isolation to rigorous and continued hygiene practices for everyone at the school and at home.

Final Step: Check the School's Specific Policy

This is a critical step. Before sending your child back, you must:

Call the school or daycare: Ask them for their specific policy regarding Hand, Foot, and Mouth Disease. Some may have stricter rules (e.g., requiring all sores to be completely gone, not just scabbed over).

Ask if a doctor's note is required: Many institutions require a note from a doctor clearing the child to return.

Summary Table: When to Return to School

Final Recommendation: Once your doctor confirms the diagnosis, follow their advice for care. When your child meets the criteria of being fever-free with healing sores, contact the school to confirm their return policy. This ensures you are following both medical and institutional guidelines.

**11. What are the commonly used and effective disinfection methods for hand, foot, and mouth disease?**

**Answer:**

Of course. This is a critical question, as the viruses that cause Hand, Foot, and Mouth Disease (specifically Coxsackievirus and Enterovirus 71) are "non-enveloped" viruses, which means they are tougher and more resistant to some common disinfectants than other germs.

Therefore, using the right disinfectant and the right method is essential.

The Critical First Step: Clean Before You Disinfect

You must always clean a surface before you disinfect it.

Cleaning: Uses soap and water to physically remove dirt, germs, and organic matter from a surface.

Disinfecting: Uses a chemical to kill the germs that remain on the surface.

If you apply a disinfectant to a dirty surface, the dirt can protect the virus and make the disinfectant less effective.

Most Effective and Commonly Used Disinfection Methods for HFMD

Here are the methods recommended by public health agencies like the CDC for killing the viruses that cause HFMD.

1. Chlorine Bleach Solution (The Gold Standard)

This is the most effective, reliable, and inexpensive option for disinfecting hard, non-porous surfaces.

**How to Prepare the Solution:**

For general disinfection: Mix 1 tablespoon of household bleach into 4 cups of water.

For a larger batch: Mix ¼ cup of household bleach into 1 gallon (16 cups) of water.

**How to Use:**

After cleaning the surface with soap and water, apply the bleach solution.

Let it sit on the surface for at least 2 minutes (this is the "contact time").

Allow it to air-dry, or wipe it down with a clean, wet cloth if it's a surface that comes into contact with food or a child's mouth (like a high chair tray).

Important Safety Precautions:

Make a fresh bleach solution daily, as it loses its effectiveness over time.

Wear gloves to protect your skin.

Ensure good ventilation (open a window).

NEVER mix bleach with ammonia or any other household cleaner, as this can create toxic gas.

Be aware that bleach can damage or discolor fabrics and some surfaces.

2. EPA-Registered Disinfectants

If you prefer a commercial product, look for disinfecting wipes or sprays that are registered with the Environmental Protection Agency (EPA) and are proven to be effective against similar tough viruses.

**What to Look For on the Label: Since most labels won't specifically list "Hand, Foot, and Mouth Disease," look for products that state they are effective against one of the following non-enveloped viruses:**

Norovirus (most common and a good indicator)

Poliovirus

Enterovirus

**How to Use:**

Read the product label carefully.

Pay close attention to the "contact time" or "dwell time." This is the amount of time the surface must remain visibly wet with the disinfectant to be effective. It can range from 30 seconds to 10 minutes.

If you wipe the surface dry before the contact time is up, you may not kill the virus.

**What to Disinfect**

Focus your efforts on high-touch surfaces and items that may have come into contact with saliva, blister fluid, or feces.

High-Touch Surfaces: Doorknobs, light switches, remote controls, toilet handles, faucet handles, countertops, and tables.

Children's Items:

Toys: Plastic, non-porous toys can be soaked in a bleach solution. For others, wipe them down thoroughly.

High Chairs & Booster Seats: Pay special attention to the tray and straps.

Potty Chairs & Diaper Changing Mats: Disinfect after every single use.

Crib Rails: Wipe them down frequently.

Bedding, Clothing, and Towels:

Wash these items in hot water if possible.

Dry them on a high heat setting in the dryer. Heat is an effective way to kill the virus. Do not "hug" dirty laundry; carry it away from your body to the washing machine.

**Methods That Are NOT Recommended for HFMD Disinfection**

It is just as important to know what not to rely on.

Standard Household Cleaners (without virucidal claims): Many all-purpose sprays are designed for cleaning, not disinfecting, or are only effective against bacteria.

Vinegar, Tea Tree Oil, and other "Natural" Cleaners: While these have some antimicrobial properties, they are not proven to be effective against the resilient enteroviruses that cause HFMD. Do not rely on them to prevent the spread of the illness.

Alcohol-Based Wipes (for surfaces): While alcohol is a disinfectant, it may not be as effective against enteroviruses as bleach or specific EPA-registered products, and it evaporates too quickly to achieve the necessary contact time on a surface. (Alcohol-based hand sanitizers are acceptable for hands when soap and water are unavailable).

**12. Are there any special concerns for infants under 3 years old who contract the disease?**

**Answer:**

Of course. This is a very important question, as Hand, Foot, and Mouth Disease (HFMD) presents several unique challenges and concerns when it affects infants and toddlers under 3 years old. While the disease is usually mild, this age group is more vulnerable for several reasons.

Here are the key special concerns for infants and toddlers with HFMD:

1. High Risk of Dehydration (The Most Common Complication)

This is the primary and most serious concern for this age group.

Why it happens: The characteristic mouth sores (herpangina) can be extremely painful. Infants and toddlers, unable to understand why it hurts to swallow, will often refuse to drink milk, formula, or water.

Why it's dangerous in infants: Infants have smaller body fluid reserves, so they can become dehydrated much more quickly than older children or adults.

Signs of Dehydration to Watch For:

Fewer Wet Diapers: This is a key indicator. Fewer than 4-6 wet diapers in a 24-hour period is a red flag.

Crying with No Tears.

Dry Mouth and Lips.

Sunken Eyes.

A sunken "soft spot" (fontanelle) on an infant's head.

Extreme Fussiness, Lethargy, or Drowsiness: The child may be unusually sleepy and difficult to wake.

Action: If you suspect dehydration, you must contact a doctor immediately. Severe dehydration may require hospitalization for intravenous (IV) fluids.

2. Difficulty with Pain Management and Diagnosis

Inability to Communicate: An infant or toddler cannot tell you, "My throat hurts," or "My mouth has sores." Instead, they will show their pain through their behavior. This can look like:

Constant, inconsolable crying.

Refusal to eat or drink (even favorite foods/drinks).

Excessive drooling because it hurts to swallow saliva.

General irritability and fussiness.

Parental Stress: This can be very distressing for parents who don't know exactly what is wrong or how to soothe their child.

3. Increased Risk of High Fever

High Temperature: While older children also get fevers, fevers in infants can sometimes be higher and more concerning.

Febrile Seizures: A rapid spike in temperature can occasionally trigger a febrile seizure in susceptible children (generally between 6 months and 5 years old). While these seizures are usually harmless, they are very frightening for parents. It's important to manage the fever according to your doctor's instructions.

4. Higher Likelihood of Atypical Rashes

Widespread Rash: Infants may have a more extensive and severe rash than older children. The rash might spread beyond the typical hands, feet, and mouth to the buttocks, legs, arms, and even the torso.

Painful Blisters: The blisters, especially in the diaper area, can be very painful, making diaper changes a difficult and stressful experience for both the child and the caregiver.

5. Increased Risk of Spreading the Virus

Normal Infant Behavior: Infants and toddlers naturally explore the world with their mouths, drool frequently, and require diaper changes. All of these are primary routes of HFMD transmission.

Caregiver Exposure: This makes parents and caregivers extremely susceptible to contracting the virus themselves. Strict and constant handwashing after every diaper change, feeding, and contact with the child's saliva is non-negotiable.

6. Risk of Secondary Infections

Bacterial Infection: If a child scratches the blisters, they can break the skin and introduce bacteria, leading to a secondary skin infection (like impetigo). It's important to keep the child's fingernails trimmed short.

Nail Loss: In some cases, children may experience peeling skin on their fingers and toes or even lose fingernails or toenails a few weeks after the illness has passed. This is painless and temporary; the nails will grow back normally.

When to Call a Doctor Immediately

For an infant or toddler with suspected HFMD, you should contact your pediatrician right away. Seek immediate medical attention if you notice:

Any signs of dehydration.

A fever that doesn't come down with age- and weight-appropriate medication.

A fever in an infant under 3 months old.

The child is unusually lethargic, sleepy, or difficult to wake.

The child seems to be in severe, uncontrollable pain.

The child has a stiff neck, headache, or seems confused (rare signs of more serious complications like meningitis).

**13.Is there a vaccine to prevent hand, foot, and mouth disease? How effective is it?**

**Answer:**

That's a very forward-thinking question. The development of a vaccine for Hand, Foot, and Mouth Disease (HFMD) is a topic of significant global health interest, but the situation is complex.

Here is a detailed breakdown of the answer:

The Short Answer: Yes, but...

Yes, vaccines to prevent Hand, Foot, and Mouth Disease do exist. However, they are not available worldwide and they only protect against one specific virus that causes the disease.

The Detailed Explanation

1. The Vaccine That Exists: The EV-A71 Vaccine

The currently available vaccines are designed to protect against Enterovirus 71 (EV-A71) only.

Why Target EV-A71? While many viruses can cause HFMD, EV-A71 is the one most often associated with severe outbreaks and serious neurological complications, such as brainstem encephalitis, meningitis, and even death, particularly in young children in the Asia-Pacific region. Therefore, developing a vaccine against this specific strain was a major public health priority.

**What it Doesn't Do: This vaccine does not protect against HFMD caused by the other common viruses, most notably Coxsackievirus A16 (which is a very frequent cause of mild HFMD worldwide) and Coxsackievirus A6 (which can cause a more severe, widespread rash).**

This means a child who is vaccinated with the EV-A71 vaccine is protected from severe disease caused by that specific virus, but can still easily get Hand, Foot, and Mouth Disease from any of the other circulating strains.

**2. How Effective Is It?**

The EV-A71 vaccine is considered highly effective at doing what it was designed to do.

Efficacy in Clinical Trials: Multiple clinical trials have shown the vaccines to have an efficacy rate of over 90% in preventing HFMD caused by the EV-A71 virus.

Protection Against Severe Disease: More importantly, the vaccine has been shown to be nearly 100% effective in preventing severe cases, hospitalizations, and deaths associated with EV-A71.

So, while it doesn't prevent all cases of HFMD, it is very successful at preventing the most dangerous form of it.

3. Global Availability: Where Is It Used?

This is the key limitation for much of the world.

Primary Use: The EV-A71 vaccine has been widely used in mainland China since its introduction in 2016. Several different versions are available there.

Other Regions: It has also been approved and is being rolled out in a few other countries in the Asia-Pacific region (e.g., Singapore, Malaysia, Cambodia, Thailand).

Not Available in the U.S. or Europe: The EV-A71 vaccine is not currently licensed or available in the United States, Canada, Europe, or Australia.

The primary reason for this is that severe, large-scale outbreaks of EV-A71 are far less common in these regions. Most HFMD cases in North America and Europe are caused by milder Coxsackievirus strains, so the public health need for a single-strain vaccine has been viewed differently.

The Future: Multivalent Vaccines

The ultimate goal for researchers is to develop a multivalent vaccine. This would be a single shot that provides protection against multiple strains at once—for example, a vaccine that targets both EV-A71 and Coxsackievirus A16. These combination vaccines are currently in various stages of research and development but are not yet available for public use.

Summary Table

**14.What is the cause of hand, foot, and mouth disease?**

**Answer:**

Of course. This is a fundamental question, and understanding the cause is key to understanding how HFMD spreads and how to prevent it.

The cause of Hand, Foot, and Mouth Disease (HFMD) is not a single virus, but a group of related viruses.

The Primary Culprits: Enteroviruses

HFMD is caused by viruses belonging to the Enterovirus genus. The name "entero" means "intestine," which is a clue: these viruses live and replicate in the digestive tract.

The two most common viruses responsible for causing HFMD are:

Coxsackievirus A16:

This is the most common cause of HFMD worldwide, particularly in the United States and Europe.

It typically causes a mild, classic form of the illness with fever, mouth sores, and a rash on the hands and feet.

Complications are very rare.

Enterovirus 71 (EV-A71):

This is the second most common cause but is of greater concern to public health officials.

While it often causes a typical case of HFMD, EV-A71 is more frequently associated with large, severe outbreaks, especially in the Asia-Pacific region.

It has a higher potential to cause serious and sometimes life-threatening neurological complications, such as viral meningitis (inflammation of the lining of the brain), encephalitis (inflammation of the brain itself), and a polio-like paralysis.

Other, Less Common Causes

In recent years, other strains of enteroviruses have also been identified as causing outbreaks of HFMD, sometimes with slightly different symptoms:

Coxsackievirus A6: This strain has become more prominent globally. It is known for causing a more severe and widespread rash that can extend to the arms, legs, buttocks, and even the face. It is also more likely to cause skin peeling and nail loss a few weeks after the illness.

Coxsackievirus A10: This is another strain that can cause HFMD, though it is less common than the others.

Key Takeaways About the Cause

It's a Virus, Not Bacteria: This is why antibiotics are completely ineffective against HFMD. Treatment focuses on managing symptoms, not killing the cause.

Multiple Strains: Because several different viruses can cause HFMD, it is possible for a person (especially a child) to get the disease more than once in their lifetime. An infection with Coxsackievirus A16 provides immunity to that specific virus, but it does not protect you from getting sick if you are later exposed to Enterovirus 71 or Coxsackievirus A6.

Route of Infection: These enteroviruses are highly contagious and spread from person to person through direct contact with an infected person's:

Respiratory droplets from a cough or sneeze.

Saliva or drool.

Fluid from the blisters.

Stool (feces), such as during a diaper change.

**15. What is the treatment for hand, foot, and mouth disease?**

**Answer:**

Excellent question. The approach to treating Hand, Foot, and Mouth Disease (HFMD) is a source of many questions for parents and caregivers because the answer can feel unsatisfying: there is no specific cure or medical treatment for HFMD.

Since HFMD is caused by a virus, antibiotics are completely ineffective. The entire focus of treatment is on supportive care, which means managing the symptoms to keep the child as comfortable as possible while their body's immune system fights off the virus. The illness typically resolves on its own within 7 to 10 days.

Here is a breakdown of the standard treatment approach, organized by symptom.

The Goal: Supportive Care and Symptom Relief

The treatment strategy targets the three main symptoms: pain/fever, mouth sores, and the skin rash.

1. Managing Pain and Fever

Over-the-Counter Pain Relievers:

Acetaminophen (e.g., Tylenol) or Ibuprofen (e.g., Advil, Motrin) can be used to reduce fever and relieve the pain from mouth sores and headaches.

Crucial: Always use the correct dosage based on your child's weight, not their age. Consult your doctor or pharmacist if you are unsure.

Aspirin Warning: NEVER give aspirin to a child or teenager, as it is linked to Reye's syndrome, a rare but very serious illness that can affect the liver and brain.

2. Soothing Painful Mouth Sores (Often the Biggest Challenge)

This is usually the most distressing symptom and the primary cause of poor eating and drinking.

Cold and Soft Foods:

Offer cold items like popsicles, ice cream, sherbet, yogurt, or cold applesauce. The cold numbs the sores and is soothing.

Serve soft foods that don't require much chewing, such as mashed potatoes, smoothies, pudding, or gelatin.

Avoid Irritating Foods:

Stay away from anything salty, spicy, or acidic (like citrus fruits, tomato sauce, or fruit juice), as these will sting the sores and cause significant pain.

Encourage Fluids:

The top priority is preventing dehydration. Offer frequent sips of cold water, milk, or a formula/milk-based oral rehydration solution.

Sucking on a bottle or even a straw can sometimes be painful. If so, try offering fluids from a cup or even with a spoon or syringe.

Topical Anesthetics (Use with caution and doctor's advice):

In some cases, a doctor might recommend a mouth-numbing spray or gel. However, these must be used carefully in young children, as numbing the entire mouth can interfere with the gag reflex and swallowing. Always consult a doctor before using any numbing products in a child's mouth.

3. Caring for the Skin Rash and Blisters

The rash on the hands and feet is not usually itchy for most children, but it can be for some. The blisters should be left alone.

Do Not Puncture the Blisters: The fluid inside can be contagious. Let them dry out and heal on their own. Poking them increases the risk of spreading the virus and causing a secondary bacterial infection.

Keep the Area Clean: Gently wash the skin with lukewarm water and mild soap, and pat it dry.

For Itching (if present): An anti-itch lotion (like calamine lotion) or a cool bath with baking soda can provide some relief.

The Most Important Thing: Preventing Dehydration

Because of the painful mouth sores, children often refuse to drink, making dehydration the most common complication of HFMD.

Monitor for signs of dehydration:

Fewer than 4-6 wet diapers in 24 hours

Crying without tears

Dry mouth and lips

Unusual sleepiness or lack of energy (lethargy)

Sunken eyes or a sunken soft spot (on infants)

If you see any signs of dehydration, contact your doctor immediately. Severe cases may require hospitalization for intravenous (IV) fluids.

Summary Table: HFMD Treatment Plan

16. The rash of hand, foot, and mouth disease.

AOf course. The rash is one of the most distinctive and defining features of Hand, Foot, and Mouth Disease (HFMD). Understanding its appearance, location, and progression is key to identifying the illness.

Here is a detailed guide to the rash of Hand, Foot, and Mouth Disease.

**1. Appearance: What It Looks Like**

The rash is not just a simple red patch; it has specific characteristics and often evolves over a couple of days.

Initial Stage: It typically starts as small, flat, red spots.

Progression to Blisters: Many of these spots quickly develop into small blisters (vesicles) containing a clear or slightly cloudy fluid. These blisters are often oval-shaped rather than perfectly round.

Appearance: They are often described as looking like "drops of water" on a red base.

Healing Stage: The blisters will eventually dry up, crust over, and disappear without scarring.

Unlike chickenpox, the HFMD rash is usually not present all over the body at the same time and tends to be concentrated in specific areas.

2. Location: "Hand, Foot, and Mouth" and Beyond

The name of the disease describes the most common locations for the rash.

Hands: On the palms and the backs of the hands.

Feet: On the soles of the feet and the tops of the feet.

Mouth (Herpangina): These are not technically a rash but are a key part of the illness. They start as small red spots on the tongue, gums, and inside of the cheeks, which then turn into painful sores or ulcers. These mouth sores are often the first symptom and the most uncomfortable part of the illness.

The rash can also commonly appear in other places, especially in infants and toddlers:

Buttocks and Groin: This is a very common location, and the rash can sometimes be mistaken for a severe diaper rash.

Knees and Elbows.

Arms and Legs.

**3. Symptoms: How It Feels**

This is a key differentiator from many other childhood rashes.

Typically Not Itchy: For most children, the rash is not itchy. This helps distinguish it from conditions like eczema or chickenpox, which are usually intensely itchy.

Can Be Painful or Tender: The blisters, especially on the hands and feet, can be tender or painful to the touch. This can make walking uncomfortable for toddlers or cause infants to be fussy when their hands are touched.

4. The Timeline of the Rash

Onset: The rash usually appears 1-2 days after the initial fever and sore throat begin.

Duration: The rash typically lasts for 5 to 7 days.

Healing: The blisters will dry up and fade on their own. It is important not to puncture or "pop" the blisters, as the fluid inside can spread the virus, and breaking the skin increases the risk of a secondary bacterial infection.

5. Atypical Rashes (Important to Know)

In recent years, outbreaks caused by a different strain of the virus (specifically Coxsackievirus A6) have led to a more severe and "atypical" rash.

More Widespread: The rash can cover much more of the body, including the arms, legs, torso, and face (especially around the mouth).

More Severe Blisters: The blisters might be larger (bullae) and more numerous.

More Likely to Be Painful or Itchy.

Later Effects: This strain is more frequently associated with skin peeling on the hands and feet and the loss of fingernails or toenails a few weeks after the illness resolves. While alarming, this process is painless, and the nails grow back normally.

Key Characteristics to Remember:

Appearance: Flat red spots that turn into small, oval-shaped blisters on a red base.

Location: Primarily palms of hands, soles of feet, and painful sores inside the mouth. Also common on the buttocks.

Feeling: Usually not itchy, but can be tender or painful.

Contagious: The fluid in the blisters is contagious, so avoid touching them.

Treatment: No specific treatment is needed for the rash itself. The focus is on keeping the area clean and managing any discomfort. It will resolve on its own.

home remedies for hand, foot and mouth disease itching

**Answer：**

Of course. While the classic rash of Hand, Foot, and Mouth Disease (HFMD) is often described as non-itchy, some children do experience significant itching (pruritus), especially with the more aggressive rash caused by the Coxsackievirus A6 strain.

Managing this itch at home is focused on soothing the skin, reducing inflammation, and preventing scratching, which can lead to secondary infections.

Here are several effective home remedies for HFMD itching.

Important First Step: Always consult with your pediatrician before trying new remedies on a young child to ensure they are safe and appropriate for their age and specific situation.

Top Home Remedies for HFMD Itching

1. Cool Compresses

This is the simplest and often most effective way to provide immediate, temporary relief.

**How to do it: Soak a clean, soft washcloth in cool water, wring it out, and gently apply it to the itchy areas for 10-15 minutes at a time. Do this several times a day as needed.**

Why it works: The coolness helps to numb the nerve endings that signal itchiness and can reduce inflammation.

2. Soothing Baths

A bath can be a great way to calm widespread itching. Avoid hot water, as it can make itching worse.

Oatmeal Bath:

**How to do it: Use a commercial colloidal oatmeal bath product (like Aveeno) or make your own by grinding 1 cup of plain, unflavored oatmeal into a fine powder using a blender. Sprinkle the powder into a tub of lukewarm water and stir. Let your child soak for 15-20 minutes.**

Why it works: Oatmeal contains compounds that have anti-inflammatory and moisturizing properties, which soothe irritated skin.

Baking Soda Bath:

**How to do it: Add about 1/4 to 1/2 cup of baking soda to a tub of lukewarm water and stir to dissolve. Let your child soak for 10-15 minutes.**

Why it works: Baking soda can help to soothe the skin and reduce irritation.

After the bath: Gently pat the skin dry with a soft towel. Rubbing can irritate the skin and blisters further.

3. Over-the-Counter Topical Relief

While not strictly "kitchen" remedies, these are readily available and considered part of standard home care.

Calamine Lotion:

**How to use it: Dab a small amount of calamine lotion onto the blisters or rash.**

Why it works: This classic pink lotion has a cooling, drying effect that provides excellent relief from itching. Be aware that it can be drying, so don't overuse it.

Coconut Oil:

**How to use it: After a bath, once the skin is patted dry, apply a thin layer of virgin coconut oil.**

Why it works: Coconut oil is moisturizing and has natural antimicrobial and anti-inflammatory properties. It helps to keep the skin from becoming overly dry and cracked.

Aloe Vera Gel:

**How to use it: Apply 100% pure aloe vera gel (preferably from the plant or a product without added alcohol or colors) to the rash.**

Why it works: Aloe vera is well-known for its cooling and anti-inflammatory effects, making it great for soothing irritated skin.

4. Lifestyle and Environmental Adjustments

Dress in Loose, Soft Clothing: Tight clothing or rough fabrics (like wool) can rub against the blisters and make itching worse. Opt for loose-fitting cotton pajamas or clothes.

Keep Fingernails Short: Trim your child's fingernails to minimize damage from scratching. Scratching can break the blisters, increasing the risk of a bacterial infection and spreading the virus.

Use Soft Bedding: Ensure bedsheets are soft and clean.

Distraction: For young children, distraction is a powerful tool. A new toy, a favorite movie, or a quiet story can take their mind off the itching.

**What to Avoid**

Scratching: Do everything you can to discourage it.

Popping Blisters: The fluid is contagious, and breaking the skin opens the door for infection.

Hot Water and Harsh Soaps: These strip the skin of its natural oils and can worsen irritation. Use mild, unscented soap and lukewarm water.

Medicated Creams (without doctor's approval): Do not use steroid creams (like hydrocortisone) or antibiotic ointments (like Neosporin) on the rash unless specifically instructed by your doctor.

When to Call the Doctor

While home remedies are great for mild itching, contact your pediatrician if:

The itching is severe and uncontrollable.

The child cannot sleep because of the itching.

The skin appears infected (e.g., oozing pus, honey-colored crusts, increased redness, swelling, or pain).

The rash seems to be getting worse instead of better after several days.

18.Monitoring the condition of severe hand, foot, and mouth disease.

**Answer：**

Excellent and critically important question. While the vast majority of Hand, Foot, and Mouth Disease (HFMD) cases are mild and resolve at home, severe HFMD is a medical emergency that requires vigilant monitoring and immediate professional care.

The key to monitoring is knowing which "red flag" symptoms signal that the virus is no longer just affecting the skin and mouth, but is potentially attacking the central nervous system (brain and spinal cord) or the cardiopulmonary system (heart and lungs). This is most often associated with Enterovirus 71 (EV-A71).

Monitoring can be broken down into two stages: what a caregiver should watch for at home, and how medical professionals monitor the condition in a hospital.

Part 1: Monitoring at Home — When to Seek Immediate Medical Help

If a child has been diagnosed with HFMD, you must be on high alert for any of the following signs. The transition from mild to severe can happen quickly, often between days 2 and 5 of the illness.

Go to the nearest Emergency Room immediately if your child shows any of these signs.

1. Neurological Red Flags (Signs of Brain and Nerve Involvement)

These are the most critical warning signs of complications like meningitis, encephalitis, or acute flaccid paralysis.

Lethargy or Altered Consciousness:

The child is unusually sleepy, difficult to wake up, or seems "out of it."

They are not responding normally to you, are confused, or do not recognize their surroundings or parents.

Myoclonic Jerks (A Key Warning Sign):

Sudden, brief, shock-like jerks of the limbs or body.

This often happens when the child is drowsy or just drifting off to sleep. They may look like they are being startled awake repeatedly.

Severe Headache, Stiff Neck, or Photophobia:

The child complains of a headache that is not relieved by pain medication.

They cry when their chin is moved toward their chest or complain of a stiff/sore neck.

They are abnormally sensitive to light (photophobia).

Instability or Limb Weakness (Ataxia):

The child is suddenly unsteady on their feet, has trouble walking, or stumbles frequently.

They may refuse to walk altogether.

There is weakness or paralysis in an arm or leg.

Inconsolable Crying or Extreme Irritability:

The child is crying constantly and cannot be soothed, which is far beyond the normal fussiness from mouth pain.

Seizures (Convulsions):

Any seizure activity requires immediate emergency care.

2. Cardiopulmonary Red Flags (Signs of Heart and Lung Involvement)

These can signal complications like pulmonary edema (fluid in the lungs) or myocarditis (inflammation of the heart muscle).

Difficulty Breathing or Rapid Breathing (Tachypnea):

Breathing seems labored, faster than normal, or shallow.

You may see the muscles between their ribs or at their neck sucking in with each breath.

They may make grunting sounds while breathing.

Changes in Skin Color:

The skin becomes pale, mottled (patchy and irregular), or has a bluish tint (cyanosis), especially around the lips or on the fingertips.

Rapid or Irregular Heartbeat:

The heart seems to be racing, even when the child does not have a fever.

Cold Sweats or Cold Limbs:

The child's skin feels cool and clammy, particularly their hands and feet, even if they have a fever.

3. Other General Red Flags

Persistent High Fever: A fever of 102°F (39°C) or higher that doesn't come down with medication or lasts more than 3 days.

Severe Dehydration: No urine output for 8-12 hours, crying with no tears, sunken eyes, extreme listlessness. This becomes an emergency when it's severe.

Part 2: Monitoring in a Hospital Setting

If a child is admitted for severe HFMD, they will be monitored intensively by a medical team. This monitoring is designed to detect any deterioration immediately and provide life-saving support.

Continuous Vital Signs Monitoring: The child will be connected to machines that continuously track:

Heart Rate and Rhythm (ECG/EKG): To watch for myocarditis or arrhythmias.

Respiratory Rate: To watch for signs of breathing difficulty.

Oxygen Saturation (Pulse Oximeter): A small clip on the finger or toe that measures the oxygen level in the blood. A drop is a critical sign of lung problems.

Blood Pressure: To monitor for shock (dangerously low blood pressure).

Frequent Neurological Assessments: Nurses and doctors will regularly check the child's neurological status by:

Assessing their level of consciousness (e.g., using the Glasgow Coma Scale).

Checking pupil response to light.

Testing reflexes and muscle strength.

Watching for any new seizures or myoclonic jerks.

Fluid and Electrolyte Management:

An intravenous (IV) line will be placed to provide fluids, correct dehydration, and administer medication.

Blood tests will be done to closely monitor electrolyte levels, which can be disrupted by the illness.

Diagnostic Imaging and Tests:

Chest X-ray: To look for fluid in the lungs (pulmonary edema).

Echocardiogram: An ultrasound of the heart to check its function if myocarditis is suspected.

MRI or CT of the Brain: May be ordered if there are significant neurological symptoms like seizures or paralysis.

Lumbar Puncture (Spinal Tap): May be performed to analyze the cerebrospinal fluid for signs of meningitis or encephalitis.

The key takeaway is to trust your instincts. You know your child best. If they seem sicker than you would expect from a "simple" virus, or if they display any of the neurological or cardiopulmonary red flags, do not wait. Seek emergency medical evaluation immediately.

**19.What should be noted for a child with hand, foot, and mouth disease receiving home care?**

**Answer:**

Of course. This is an essential question for any parent or caregiver managing Hand, Foot, and Mouth Disease (HFMD) at home. The focus of home care is on four main goals: providing comfort, ensuring hydration, preventing the spread of the virus, and knowing when to seek professional medical help.

Here is a comprehensive checklist of what you should note and do when caring for a child with HFMD at home.

1. Prioritize Hydration Above All Else

The painful mouth sores make swallowing difficult, and dehydration is the most common reason children with HFMD need hospital care.

Offer Fluids Constantly: Provide small, frequent sips of cool liquids. Don't wait for them to ask.

**What to Offer:**

Best choices: Water, milk, or an oral rehydration solution (like Pedialyte).

Good alternatives: Milk-based smoothies, very diluted juice.

Avoid: Acidic drinks like orange juice or lemonade, which will sting the sores.

**How to Offer: If sucking on a bottle or straw is painful, try using a cup, a spoon, or even a syringe to gently squirt liquid into the side of their mouth.**

Monitor Output: Keep track of wet diapers or trips to the bathroom. Fewer than 4 wet diapers in 24 hours is a key sign of dehydration.

2. Manage Pain and Fever Effectively

A comfortable child is more likely to rest and drink.

Use Pain Relievers Correctly:

Give acetaminophen (Tylenol) or ibuprofen (Advil, Motrin) to manage fever and pain from the mouth sores.

Crucial: Dose according to your child's weight, not their age. Check the package or ask your pharmacist.

NEVER give aspirin to a child due to the risk of Reye's syndrome.

Time it Right: Consider giving a dose of pain reliever about 30 minutes before a meal to make eating and drinking more tolerable.

3. Soothe Mouth Sores and Choose Foods Wisely

Think Soft and Cold:

Offer popsicles, ice cream, yogurt, cold applesauce, pudding, and smoothies. The cold numbs the pain.

Serve soft, bland foods that don't require chewing, like mashed potatoes, Jell-O, or soup (once cooled).

Avoid Irritating Foods: Stay away from anything salty (chips), spicy (salsa), or acidic (tomatoes, citrus fruits).

4. Care for the Rash, But Don't Fixate on It

Leave Blisters Alone: Do not puncture or squeeze the blisters. The fluid is contagious, and breaking the skin increases the risk of a secondary bacterial infection. Let them dry out on their own.

Keep it Clean: Gently wash the skin with mild soap and lukewarm water, then pat dry.

Control Itching (if present):

Give a cool bath with baking soda or colloidal oatmeal (like Aveeno).

Apply calamine lotion to itchy spots.

Dress your child in loose, soft, cotton clothing.

5. Practice Strict Hygiene to Prevent Spreading

HFMD is extremely contagious. Assume your child is a little virus-spreading machine and take action.

Handwashing is Your Best Weapon: Wash your hands thoroughly with soap and water after every diaper change, after touching blisters, after wiping your child's nose or drool, and before preparing food.

Isolate the Child (as much as possible):

Keep the child home from school, daycare, and public places until they are cleared to return (see below).

Try to limit close contact like hugging and kissing with other family members, especially siblings.

Do Not Share: Do not share cups, utensils, towels, or toothbrushes.

Disinfect Surfaces: The virus can live on surfaces for hours. Regularly clean and disinfect high-touch surfaces like doorknobs, remote controls, faucets, and especially toys. Use a solution of bleach and water (1 tablespoon of bleach per 4 cups of water) or an EPA-registered disinfectant wipe.

6. Monitor for Red Flags (When to Call the Doctor or Go to the ER)

This is the most important monitoring task for a caregiver. While most cases are mild, severe complications can develop quickly.

Call your doctor for advice if:

Your child is refusing to drink and you are worried about dehydration.

Symptoms are not improving after 7-10 days.

The fever is high and persistent.

The child seems very uncomfortable and is not getting relief from home care measures.

Go to the Emergency Room IMMEDIATELY if you see any of these signs of severe disease:

Signs of Dehydration: Crying with no tears, sunken eyes, no wet diapers for 8-12 hours, extreme sleepiness.

Neurological Signs:

Lethargy: Unusually sleepy, difficult to wake up, or confused.

Severe Headache or Stiff Neck.

Myoclonic Jerks: Sudden, repeated, twitch-like movements, especially when tired or drifting off to sleep.

Unsteadiness: Trouble walking, stumbling, or refusing to walk.

Seizures.

Breathing Problems: Rapid breathing, labored breathing, or skin turning pale or blue.

7. Know When to Return to School or Daycare

Policies can vary, but the general guidelines are:

The child must be fever-free for at least 24 hours without the use of fever-reducing medication.

The mouth sores are no longer present or have healed.

The child feels well enough to participate in activities.

Note: The blisters on the hands and feet may still be present or healing, but as long as they are not open and weeping, many schools will allow a return if other criteria are met. Check with your specific school or daycare for their policy.

20.Who is susceptible to hand, foot, and mouth disease?

**Answer:**

Excellent question. The answer is broader than many people think. While Hand, Foot, and Mouth Disease (HFMD) is famously known as a childhood illness, susceptibility isn't strictly limited to kids.

Here is a breakdown of who is most susceptible, starting with the primary group.

1. The Most Susceptible Group: Infants and Young Children

Infants and children under the age of 5 are, by far, the most susceptible to getting and showing symptoms of HFMD.

There are two main reasons for this:

Lack of Immunity: Their immune systems are still developing and have not yet been exposed to the many viruses that cause HFMD (like Coxsackievirus and Enterovirus). They haven't had the chance to build up the necessary antibodies to fight off the infection effectively. Once a person has had HFMD caused by a specific virus strain, they typically develop immunity to that strain, but they can still get HFMD again from a different strain.

Environment and Behavior: The settings where young children spend their time are perfect for spreading the virus.

Close Contact: Children in daycare centers, preschools, and kindergartens are in constant close contact—they play together, touch the same toys, and are not always great at covering coughs or sneezes.

Hygiene Habits: Young children are still learning proper hygiene. They frequently put their hands in their mouths, touch their faces, and are less diligent about handwashing.

Fecal-Oral Route: The virus is shed in feces for several weeks, even after symptoms are gone. Diaper changes are a major route of transmission, making daycare workers and parents of infants/toddlers particularly vulnerable to exposure.

2. Older Children and Teenagers

While less common, older children and teenagers can still get HFMD.

This usually happens if they were not exposed to a particular strain of the virus when they were younger.

The illness in this age group is typically mild, sometimes so mild that it's mistaken for a simple cold or sore throat, or they may have no symptoms at all (asymptomatic).

3. Adults

Yes, adults can absolutely get Hand, Foot, and Mouth Disease. However, it is much less common for two reasons:

Existing Immunity: Most adults were exposed to various common strains of the virus during their own childhood and have protective antibodies.

Asymptomatic Infection: Even if an adult gets infected, their mature immune system often fights it off without them ever showing symptoms. They can, however, still be a carrier and spread the virus to others.

When adults do get symptomatic HFMD, it can be surprisingly unpleasant. Many adults report that the symptoms—especially the painful mouth sores and sore throat—are more severe and miserable than what is typically seen in children.

Adults who are most at risk include:

Parents and Caregivers: Those with young children in the home, especially during an active infection.

Childcare Workers and Teachers: People who work in daycares, preschools, and elementary schools are constantly exposed.

Immunocompromised Individuals: Adults with weakened immune systems due to medical conditions (like HIV/AIDS) or treatments (like chemotherapy) are more susceptible to infection and may experience a more severe illness.

Summary Table of Susceptibility

In short: Everyone is technically susceptible, but your risk of actually getting sick from HFMD decreases significantly with age and previous exposure.
