# Supplementary material for: Evaluation of generative AI-driven chatbots as sources of consumer health information on hand, foot, and mouth disease: a cross-sectional comparative study of safety, accuracy, information quality, readability, and empathy
Source: Front Public Health. 2026 Aug 11;14:1924162. doi: 10.3389/fpubh.2026.1924162 (PMC13503565; doi:10.3389/fpubh.2026.1924162)
Supplement: Supplementary file 5 [file Table_1.docx]

## **Supplementary Table：**

**Supplementary Table S1. Characteristics and deployment context of evaluated AI-driven chatbots.**

| **Chatbot** | **Provider** | **Version/model identifier** | **Access route** | **Access plan** | **Open-source status** | **Query dates** | **Location** |
| --- | --- | --- | --- | --- | --- | --- | --- |
| ChatGPT | OpenAI | ChatGPT-4o | Official web interface | ChatGPT Plus | Proprietary deployed chatbot | April 2, 2026 | Mudanjiang, China |
| Copilot | Microsoft | Version not publicly disclosed | Official web interface | Free/paid plan | Proprietary deployed chatbot | April 3, 2026 | Mudanjiang, China |
| Gemini | Google | Gemini 2.5 Pro | Gemini Advanced | Paid | Proprietary deployed chatbot | April 3, 2026 | Mudanjiang, China |
| Doubao | ByteDance/Doubao AI | Version displayed on platform | Official web interface | Free/paid plan | Proprietary deployed chatbot | April 4, 2026 | Mudanjiang, China |
| DeepSeek | DeepSeek AI | V3.2-Exp | Official web interface | Free/paid plan | Proprietary deployed chatbot | April 5, 2026 | Mudanjiang, China |

**Supplementary Table S2. Inter-rater agreement**

| **Metric** | **Agreement index** | **Estimate** | **95% CI** | ***P* value** | **Complete items** | **No. of raters** | **Note** |
| --- | --- | --- | --- | --- | --- | --- | --- |
| Safety | Fleiss' kappa | 0.878 | 0.789-0.964 | <0.001 | 100 | 5 | Primary agreement index for Safety; 95% CI based on 3000 nonparametric bootstrap resamples. A negative lower CI may occur when unsafe outcomes are extremely sparse because kappa can theoretically be below 0; this does not by itself indicate a coding error. |
| Accuracy | ICC(2,1) | 0.864 | 0.824-0.899 | <0.001 | 100 | 5 | Two-way random-effects, absolute-agreement, single-measure ICC. |
| Empathy | ICC(2,1) | 0.863 | 0.823-0.898 | <0.001 | 100 | 5 | Two-way random-effects, absolute-agreement, single-measure ICC. |
| DISCERN | ICC(2,1) | 0.849 | 0.805-0.887 | <0.001 | 100 | 5 | Two-way random-effects, absolute-agreement, single-measure ICC. |
| EQIP | ICC(2,1) | 0.847 | 0.803-0.886 | <0.001 | 100 | 5 | Two-way random-effects, absolute-agreement, single-measure ICC. |
| JAMA | ICC(2,1) | 0.851 | 0.808-0.889 | <0.001 | 100 | 5 | Two-way random-effects, absolute-agreement, single-measure ICC. |
| GQS | ICC(2,1) | 0.850 | 0.806-0.888 | <0.001 | 100 | 5 | Two-way random-effects, absolute-agreement, single-measure ICC. |

Note: Safety was assessed using Fleiss' kappa. Accuracy, Empathy, DISCERN, EQIP, JAMA and GQS were assessed using ICC(2,1).

## **Supplementary Table S3. Pairwise comparisons of safety**

| **Comparison** | **N pairs** | **Safe rate difference, %** | **Safe rate difference 95% CI** | **Safe to unsafe, n** | **Unsafe to safe, n** | **Matched OR** | **Matched OR 95% CI** | **Adjusted P** | **Note** |
| --- | --- | --- | --- | --- | --- | --- | --- | --- | --- |
| ChatGPT vs Gemini | 20 | 5.00 | -10.00-20.00 | 2 | 1 | 2.00 | 0.18-22.06 | 1.000 | Pairwise McNemar test |
| ChatGPT vs Copilot | 20 | 5.00 | 0.00-15.00 | 1 | 0 | 3.00 | 0.12-73.65 | 1.000 | Pairwise McNemar test |
| ChatGPT vs DeepSeek | 20 | 5.00 | 0.00-15.00 | 1 | 0 | 3.00 | 0.12-73.65 | 1.000 | Pairwise McNemar test |
| ChatGPT vs Doubao | 20 | 10.00 | 0.00-25.00 | 2 | 0 | 5.00 | 0.24-104.15 | 1.000 | Pairwise McNemar test |
| Gemini vs Copilot | 20 | 0.00 | -20.00-20.00 | 2 | 2 | 1.00 | 0.14-7.10 | 1.000 | Pairwise McNemar test |
| Gemini vs DeepSeek | 20 | 0.00 | -15.00-15.00 | 1 | 1 | 1.00 | 0.06-15.99 | 1.000 | Pairwise McNemar test |
| Gemini vs Doubao | 20 | 5.00 | -15.00-25.00 | 3 | 2 | 1.50 | 0.25-8.98 | 1.000 | Pairwise McNemar test |
| Copilot vs DeepSeek | 20 | 0.00 | -15.00-15.00 | 1 | 1 | 1.00 | 0.06-15.99 | 1.000 | Pairwise McNemar test |
| Copilot vs Doubao | 20 | 5.00 | 0.00-15.00 | 1 | 0 | 3.00 | 0.12-73.65 | 1.000 | Pairwise McNemar test |
| DeepSeek vs Doubao | 20 | 5.00 | -10.00-20.00 | 2 | 1 | 2.00 | 0.18-22.06 | 1.000 | Pairwise McNemar test |

Note: Pairwise comparisons were performed using McNemar tests with Benjamini-Hochberg correction. Safe-rate differences and their bootstrap 95% CIs are reported.

## Supplementary Table **S**4**.** Significant pairwise comparisons for non-binary outcomes

| **Metric** | **Comparison** | **N pairs** | **Mean difference** | **Mean difference 95% CI** | **Median difference** | **Median difference 95% CI** | **Rank-biserial correlation** | **Rank-biserial 95% CI** | **Adjusted P** |
| --- | --- | --- | --- | --- | --- | --- | --- | --- | --- |
| ARI | ChatGPT vs Gemini | 20 | -1.49 | -2.09--0.94 | -2.20 | -2.39--0.21 | -0.857 | -1.000--0.591 | 0.002 |
| ARI | ChatGPT vs Copilot | 20 | -2.03 | -2.88--1.20 | -1.86 | -3.33--1.03 | -0.867 | -1.000--0.562 | 0.002 |
| ARI | ChatGPT vs DeepSeek | 20 | -1.55 | -2.21--0.93 | -1.88 | -2.42--0.41 | -0.800 | -0.971--0.476 | 0.004 |
| ARI | Gemini vs Doubao | 20 | 1.70 | 1.05-2.36 | 1.90 | 1.00-2.31 | 0.886 | 0.619-1.000 | 0.002 |
| ARI | Copilot vs Doubao | 20 | 2.23 | 1.39-3.01 | 2.56 | 1.40-3.27 | 0.914 | 0.714-1.000 | 0.002 |
| ARI | DeepSeek vs Doubao | 20 | 1.76 | 0.78-2.78 | 1.53 | 0.38-2.84 | 0.737 | 0.362-0.958 | 0.009 |
| CL | ChatGPT vs Copilot | 20 | -1.51 | -2.22--0.78 | -1.46 | -2.75--0.84 | -0.832 | -1.000--0.505 | 0.008 |
| CL | Gemini vs Copilot | 20 | -1.14 | -1.56--0.68 | -1.33 | -1.72--0.77 | -0.876 | -1.000--0.629 | 0.006 |
| CL | Copilot vs Doubao | 20 | 1.41 | 0.70-2.07 | 1.66 | 0.54-2.44 | 0.781 | 0.467-0.981 | 0.008 |
| FKGL | ChatGPT vs Gemini | 20 | -1.73 | -2.19--1.25 | -1.85 | -2.51--1.04 | -0.971 | -1.000--0.857 | 0.001 |
| FKGL | ChatGPT vs Copilot | 20 | -1.40 | -2.08--0.72 | -1.26 | -2.79--0.40 | -0.762 | -0.971--0.419 | 0.005 |
| FKGL | ChatGPT vs DeepSeek | 20 | -1.64 | -2.14--1.11 | -1.97 | -2.35--0.80 | -0.905 | -0.991--0.657 | 0.001 |
| FKGL | Gemini vs Doubao | 20 | 1.81 | 1.23-2.43 | 1.81 | 1.22-2.23 | 0.952 | 0.819-1.000 | 0.001 |
| FKGL | Copilot vs Doubao | 20 | 1.48 | 0.83-2.18 | 1.49 | 0.52-2.19 | 0.829 | 0.543-0.991 | 0.003 |
| FKGL | DeepSeek vs Doubao | 20 | 1.72 | 0.88-2.56 | 1.86 | 0.40-2.35 | 0.800 | 0.476-0.971 | 0.004 |
| SMOG | ChatGPT vs Gemini | 20 | -1.30 | -1.63--0.94 | -1.42 | -1.86--0.78 | -0.971 | -1.000--0.857 | <0.001 |
| SMOG | ChatGPT vs Copilot | 20 | -0.87 | -1.31--0.43 | -0.66 | -1.51--0.30 | -0.781 | -1.000--0.438 | 0.004 |
| SMOG | ChatGPT vs DeepSeek | 20 | -1.39 | -1.82--0.98 | -1.38 | -2.08--0.61 | -0.962 | -1.000--0.829 | <0.001 |
| SMOG | Gemini vs Doubao | 20 | 1.68 | 1.24-2.12 | 1.82 | 0.94-2.32 | 0.976 | 0.886-1.000 | <0.001 |
| SMOG | Copilot vs Doubao | 20 | 1.26 | 0.82-1.73 | 0.96 | 0.61-1.94 | 0.962 | 0.829-1.000 | <0.001 |
| SMOG | DeepSeek vs Doubao | 20 | 1.77 | 1.22-2.35 | 1.93 | 0.70-2.60 | 0.971 | 0.857-1.000 | <0.001 |
| FRES | ChatGPT vs Copilot | 20 | 4.90 | 1.55-7.95 | 5.50 | 1.50-9.50 | 0.643 | 0.219-0.971 | 0.041 |
| FRES | ChatGPT vs DeepSeek | 20 | 6.65 | 3.05-10.05 | 8.00 | 4.00-11.00 | 0.737 | 0.348-0.988 | 0.041 |
| FRES | DeepSeek vs Doubao | 20 | -5.80 | -9.70--2.00 | -6.50 | -10.00--0.50 | -0.678 | -0.947--0.250 | 0.041 |

Note: Pairwise comparisons were performed using paired Wilcoxon signed-rank tests with Benjamini-Hochberg correction. Paired mean/median differences and rank-biserial correlations are reported with bootstrap 95% CIs. Only significant comparisons are shown.

## Supplementary Table S5. Significant pairwise comparisons of safety

**Note**

No significant pairwise safety difference after Benjamini-Hochberg correction.

Note: Pairwise comparisons were performed using McNemar tests with Benjamini-Hochberg correction. Safe-rate differences and their bootstrap 95% CIs are reported. Only significant comparisons are shown.

## **Supplementary Table S6. All pairwise comparisons for non-binary outcomes**

| **Metric** | **Comparison** | **N pairs** | **Mean difference** | **Mean difference 95% CI** | **Median difference** | **Median difference 95% CI** | **Rank-biserial correlation** | **Rank-biserial 95% CI** | **Adjusted P** | **Note** |
| --- | --- | --- | --- | --- | --- | --- | --- | --- | --- | --- |
| Accuracy | ChatGPT vs Gemini | 20 | 0.00 | -0.70-0.70 | 0.00 | -1.00-1.00 | 0.017 | -0.517-0.588 | 0.977 | Paired Wilcoxon signed-rank test |
| Accuracy | ChatGPT vs Copilot | 20 | 0.45 | -0.25-1.15 | 0.00 | -1.00-1.50 | 0.331 | -0.206-0.765 | 0.893 | Paired Wilcoxon signed-rank test |
| Accuracy | ChatGPT vs DeepSeek | 20 | 0.25 | -0.50-1.05 | 0.00 | -1.00-1.00 | 0.147 | -0.400-0.661 | 0.893 | Paired Wilcoxon signed-rank test |
| Accuracy | ChatGPT vs Doubao | 20 | 0.25 | -0.70-1.15 | 0.50 | -1.00-1.50 | 0.163 | -0.368-0.692 | 0.893 | Paired Wilcoxon signed-rank test |
| Accuracy | Gemini vs Copilot | 20 | 0.45 | -0.20-1.15 | 1.00 | -1.00-1.00 | 0.281 | -0.255-0.716 | 0.893 | Paired Wilcoxon signed-rank test |
| Accuracy | Gemini vs DeepSeek | 20 | 0.25 | -0.60-1.10 | 0.50 | -0.50-1.50 | 0.110 | -0.433-0.680 | 0.893 | Paired Wilcoxon signed-rank test |
| Accuracy | Gemini vs Doubao | 20 | 0.25 | -0.55-1.10 | 0.50 | -1.00-1.00 | 0.116 | -0.386-0.579 | 0.893 | Paired Wilcoxon signed-rank test |
| Accuracy | Copilot vs DeepSeek | 20 | -0.20 | -1.00-0.65 | 0.00 | -1.00-0.00 | -0.154 | -0.782-0.486 | 0.893 | Paired Wilcoxon signed-rank test |
| Accuracy | Copilot vs Doubao | 20 | -0.20 | -1.20-0.80 | -0.50 | -2.00-2.00 | -0.105 | -0.574-0.426 | 0.893 | Paired Wilcoxon signed-rank test |
| Accuracy | DeepSeek vs Doubao | 20 | 0.00 | -0.95-0.90 | 0.00 | -1.00-1.00 | 0.018 | -0.503-0.536 | 0.977 | Paired Wilcoxon signed-rank test |
| Empathy | ChatGPT vs Gemini | 20 | 0.40 | -0.40-1.20 | 0.50 | -1.00-1.00 | 0.216 | -0.314-0.675 | 0.529 | Paired Wilcoxon signed-rank test |
| Empathy | ChatGPT vs Copilot | 20 | 0.80 | 0.00-1.65 | 1.00 | -0.50-2.00 | 0.471 | -0.039-0.850 | 0.177 | Paired Wilcoxon signed-rank test |
| Empathy | ChatGPT vs DeepSeek | 20 | -0.15 | -0.90-0.75 | -0.50 | -1.00-0.00 | -0.117 | -0.736-0.426 | 0.750 | Paired Wilcoxon signed-rank test |
| Empathy | ChatGPT vs Doubao | 20 | 1.00 | 0.15-1.90 | 1.00 | -0.50-2.00 | 0.529 | 0.041-0.882 | 0.177 | Paired Wilcoxon signed-rank test |
| Empathy | Gemini vs Copilot | 20 | 0.40 | -0.45-1.20 | 0.50 | -0.50-1.50 | 0.235 | -0.333-0.714 | 0.529 | Paired Wilcoxon signed-rank test |
| Empathy | Gemini vs DeepSeek | 20 | -0.55 | -1.25-0.20 | -1.00 | -1.00-0.00 | -0.441 | -0.885-0.073 | 0.194 | Paired Wilcoxon signed-rank test |
| Empathy | Gemini vs Doubao | 20 | 0.60 | 0.15-1.10 | 0.50 | 0.00-1.00 | 0.637 | 0.167-1.000 | 0.177 | Paired Wilcoxon signed-rank test |
| Empathy | Copilot vs DeepSeek | 20 | -0.95 | -1.90-0.05 | -1.50 | -2.50-0.50 | -0.468 | -0.877-0.041 | 0.177 | Paired Wilcoxon signed-rank test |
| Empathy | Copilot vs Doubao | 20 | 0.20 | -0.50-0.95 | 0.00 | -1.00-1.00 | 0.100 | -0.507-0.641 | 0.750 | Paired Wilcoxon signed-rank test |
| Empathy | DeepSeek vs Doubao | 20 | 1.15 | 0.25-2.00 | 1.00 | 1.00-2.00 | 0.600 | 0.163-0.952 | 0.177 | Paired Wilcoxon signed-rank test |
| DISCERN | ChatGPT vs Gemini | 20 | 1.50 | -0.25-3.25 | 1.00 | -1.00-3.00 | 0.316 | -0.176-0.739 | 0.483 | Paired Wilcoxon signed-rank test |
| DISCERN | ChatGPT vs Copilot | 20 | 2.40 | 0.75-4.10 | 3.00 | -0.50-5.00 | 0.621 | 0.170-0.905 | 0.178 | Paired Wilcoxon signed-rank test |
| DISCERN | ChatGPT vs DeepSeek | 20 | 1.50 | -0.70-3.60 | 1.50 | -2.00-5.00 | 0.310 | -0.184-0.733 | 0.483 | Paired Wilcoxon signed-rank test |
| DISCERN | ChatGPT vs Doubao | 20 | 1.85 | -0.10-3.70 | 1.50 | -1.50-5.00 | 0.400 | -0.086-0.771 | 0.483 | Paired Wilcoxon signed-rank test |
| DISCERN | Gemini vs Copilot | 20 | 0.90 | -0.90-2.70 | 1.50 | -0.50-4.00 | 0.261 | -0.298-0.775 | 0.589 | Paired Wilcoxon signed-rank test |
| DISCERN | Gemini vs DeepSeek | 20 | 0.00 | -2.30-2.45 | -1.00 | -2.50-1.50 | -0.089 | -0.653-0.416 | 0.808 | Paired Wilcoxon signed-rank test |
| DISCERN | Gemini vs Doubao | 20 | 0.35 | -1.25-2.10 | 0.50 | -2.00-1.50 | 0.068 | -0.452-0.547 | 0.808 | Paired Wilcoxon signed-rank test |
| DISCERN | Copilot vs DeepSeek | 20 | -0.90 | -2.80-1.10 | -1.50 | -4.00-1.00 | -0.326 | -0.781-0.175 | 0.483 | Paired Wilcoxon signed-rank test |
| DISCERN | Copilot vs Doubao | 20 | -0.55 | -2.50-1.35 | -0.50 | -2.00-2.50 | -0.105 | -0.603-0.442 | 0.808 | Paired Wilcoxon signed-rank test |
| DISCERN | DeepSeek vs Doubao | 20 | 0.35 | -1.40-2.10 | -0.50 | -3.00-3.50 | 0.110 | -0.439-0.584 | 0.808 | Paired Wilcoxon signed-rank test |
| EQIP | ChatGPT vs Gemini | 20 | -5.25 | -12.51-2.00 | -5.00 | -10.00-2.50 | -0.346 | -0.801-0.175 | 0.740 | Paired Wilcoxon signed-rank test |
| EQIP | ChatGPT vs Copilot | 20 | 0.75 | -5.25-6.75 | 0.00 | -7.50-7.50 | 0.066 | -0.464-0.647 | 0.927 | Paired Wilcoxon signed-rank test |
| EQIP | ChatGPT vs DeepSeek | 20 | -1.75 | -8.25-4.50 | -2.50 | -10.00-5.00 | -0.150 | -0.705-0.411 | 0.858 | Paired Wilcoxon signed-rank test |
| EQIP | ChatGPT vs Doubao | 20 | -1.00 | -9.00-7.50 | 0.00 | -10.00-5.00 | -0.088 | -0.614-0.448 | 0.927 | Paired Wilcoxon signed-rank test |
| EQIP | Gemini vs Copilot | 20 | 6.00 | -1.75-13.50 | 7.50 | 0.00-15.00 | 0.386 | -0.147-0.846 | 0.740 | Paired Wilcoxon signed-rank test |
| EQIP | Gemini vs DeepSeek | 20 | 3.50 | -4.25-10.26 | 5.00 | -5.00-15.00 | 0.292 | -0.233-0.765 | 0.740 | Paired Wilcoxon signed-rank test |
| EQIP | Gemini vs Doubao | 20 | 4.25 | -3.00-11.75 | 5.00 | -5.00-12.50 | 0.275 | -0.271-0.737 | 0.740 | Paired Wilcoxon signed-rank test |
| EQIP | Copilot vs DeepSeek | 20 | -2.50 | -8.50-4.00 | -5.00 | -10.00-5.00 | -0.232 | -0.700-0.275 | 0.740 | Paired Wilcoxon signed-rank test |
| EQIP | Copilot vs Doubao | 20 | -1.75 | -9.00-6.25 | -5.00 | -12.50-7.50 | -0.210 | -0.700-0.310 | 0.740 | Paired Wilcoxon signed-rank test |
| EQIP | DeepSeek vs Doubao | 20 | 0.75 | -6.25-8.00 | 0.00 | -10.00-15.00 | 0.029 | -0.507-0.563 | 0.930 | Paired Wilcoxon signed-rank test |
| JAMA | ChatGPT vs Gemini | 20 | -0.50 | -1.05-0.10 | -1.00 | -1.00-0.00 | -0.375 | -0.853-0.154 | 0.719 | Paired Wilcoxon signed-rank test |
| JAMA | ChatGPT vs Copilot | 20 | -0.65 | -1.40-0.10 | -0.50 | -2.00-0.00 | -0.475 | -0.900-0.073 | 0.719 | Paired Wilcoxon signed-rank test |
| JAMA | ChatGPT vs DeepSeek | 20 | -0.35 | -0.90-0.25 | -0.50 | -1.00-0.00 | -0.308 | -0.795-0.263 | 0.719 | Paired Wilcoxon signed-rank test |
| JAMA | ChatGPT vs Doubao | 20 | -0.25 | -0.80-0.25 | 0.00 | -1.00-0.50 | -0.248 | -0.737-0.341 | 0.719 | Paired Wilcoxon signed-rank test |
| JAMA | Gemini vs Copilot | 20 | -0.15 | -1.00-0.60 | 0.00 | -1.00-1.00 | -0.046 | -0.544-0.497 | 0.884 | Paired Wilcoxon signed-rank test |
| JAMA | Gemini vs DeepSeek | 20 | 0.15 | -0.50-0.75 | 0.00 | 0.00-1.00 | 0.165 | -0.445-0.817 | 0.769 | Paired Wilcoxon signed-rank test |
| JAMA | Gemini vs Doubao | 20 | 0.25 | -0.35-0.85 | 0.00 | -0.50-1.00 | 0.200 | -0.400-0.713 | 0.719 | Paired Wilcoxon signed-rank test |
| JAMA | Copilot vs DeepSeek | 20 | 0.30 | -0.35-0.95 | 0.50 | -1.00-1.00 | 0.209 | -0.338-0.684 | 0.719 | Paired Wilcoxon signed-rank test |
| JAMA | Copilot vs Doubao | 20 | 0.40 | -0.30-1.05 | 1.00 | 0.00-1.00 | 0.301 | -0.218-0.809 | 0.719 | Paired Wilcoxon signed-rank test |
| JAMA | DeepSeek vs Doubao | 20 | 0.10 | -0.55-0.75 | 0.00 | -1.00-1.00 | 0.092 | -0.478-0.647 | 0.857 | Paired Wilcoxon signed-rank test |
| GQS | ChatGPT vs Gemini | 20 | -0.05 | -0.50-0.40 | 0.00 | -1.00-1.00 | -0.073 | -0.581-0.500 | 0.935 | Paired Wilcoxon signed-rank test |
| GQS | ChatGPT vs Copilot | 20 | -0.35 | -0.70-0.00 | 0.00 | -1.00-0.00 | -0.644 | -1.000--0.091 | 0.804 | Paired Wilcoxon signed-rank test |
| GQS | ChatGPT vs DeepSeek | 20 | -0.20 | -0.65-0.25 | 0.00 | -1.00-0.00 | -0.288 | -0.855-0.333 | 0.805 | Paired Wilcoxon signed-rank test |
| GQS | ChatGPT vs Doubao | 20 | -0.25 | -0.75-0.25 | 0.00 | -1.00-1.00 | -0.300 | -0.736-0.283 | 0.805 | Paired Wilcoxon signed-rank test |
| GQS | Gemini vs Copilot | 20 | -0.30 | -0.85-0.25 | -0.50 | -1.00-0.00 | -0.257 | -0.850-0.330 | 0.805 | Paired Wilcoxon signed-rank test |
| GQS | Gemini vs DeepSeek | 20 | -0.15 | -0.70-0.40 | -0.50 | -1.00-1.00 | -0.118 | -0.629-0.386 | 0.935 | Paired Wilcoxon signed-rank test |
| GQS | Gemini vs Doubao | 20 | -0.20 | -0.60-0.20 | 0.00 | -0.50-0.00 | -0.333 | -1.000-0.418 | 0.805 | Paired Wilcoxon signed-rank test |
| GQS | Copilot vs DeepSeek | 20 | 0.15 | -0.30-0.60 | 0.00 | 0.00-1.00 | 0.179 | -0.429-0.818 | 0.935 | Paired Wilcoxon signed-rank test |
| GQS | Copilot vs Doubao | 20 | 0.10 | -0.55-0.70 | 0.00 | 0.00-1.00 | 0.000 | -0.543-0.667 | 1.000 | Paired Wilcoxon signed-rank test |
| GQS | DeepSeek vs Doubao | 20 | -0.05 | -0.50-0.40 | 0.00 | -1.00-1.00 | -0.067 | -0.596-0.510 | 0.935 | Paired Wilcoxon signed-rank test |
| ARI | ChatGPT vs Gemini | 20 | -1.49 | -2.09--0.94 | -2.20 | -2.39--0.21 | -0.857 | -1.000--0.591 | 0.002 | Paired Wilcoxon signed-rank test |
| ARI | ChatGPT vs Copilot | 20 | -2.03 | -2.88--1.20 | -1.86 | -3.33--1.03 | -0.867 | -1.000--0.562 | 0.002 | Paired Wilcoxon signed-rank test |
| ARI | ChatGPT vs DeepSeek | 20 | -1.55 | -2.21--0.93 | -1.88 | -2.42--0.41 | -0.800 | -0.971--0.476 | 0.004 | Paired Wilcoxon signed-rank test |
| ARI | ChatGPT vs Doubao | 20 | 0.20 | -0.44-0.83 | 0.07 | -0.78-1.22 | 0.124 | -0.381-0.610 | 0.712 | Paired Wilcoxon signed-rank test |
| ARI | Gemini vs Copilot | 20 | -0.53 | -1.20-0.15 | -0.67 | -1.45--0.01 | -0.400 | -0.857-0.086 | 0.188 | Paired Wilcoxon signed-rank test |
| ARI | Gemini vs DeepSeek | 20 | -0.06 | -0.89-0.69 | 0.30 | -0.90-1.09 | 0.062 | -0.429-0.581 | 0.823 | Paired Wilcoxon signed-rank test |
| ARI | Gemini vs Doubao | 20 | 1.70 | 1.05-2.36 | 1.90 | 1.00-2.31 | 0.886 | 0.619-1.000 | 0.002 | Paired Wilcoxon signed-rank test |
| ARI | Copilot vs DeepSeek | 20 | 0.47 | -0.39-1.33 | 0.66 | -0.80-1.35 | 0.209 | -0.295-0.695 | 0.528 | Paired Wilcoxon signed-rank test |
| ARI | Copilot vs Doubao | 20 | 2.23 | 1.39-3.01 | 2.56 | 1.40-3.27 | 0.914 | 0.714-1.000 | 0.002 | Paired Wilcoxon signed-rank test |
| ARI | DeepSeek vs Doubao | 20 | 1.76 | 0.78-2.78 | 1.53 | 0.38-2.84 | 0.737 | 0.362-0.958 | 0.009 | Paired Wilcoxon signed-rank test |
| CL | ChatGPT vs Gemini | 20 | -0.37 | -0.92-0.22 | -0.69 | -1.03-0.11 | -0.329 | -0.781-0.162 | 0.341 | Paired Wilcoxon signed-rank test |
| CL | ChatGPT vs Copilot | 20 | -1.51 | -2.22--0.78 | -1.46 | -2.75--0.84 | -0.832 | -1.000--0.505 | 0.008 | Paired Wilcoxon signed-rank test |
| CL | ChatGPT vs DeepSeek | 20 | -0.81 | -1.50--0.09 | -1.12 | -1.83-0.40 | -0.495 | -0.838--0.038 | 0.109 | Paired Wilcoxon signed-rank test |
| CL | ChatGPT vs Doubao | 20 | -0.10 | -0.87-0.71 | -0.34 | -1.23-1.26 | -0.067 | -0.562-0.438 | 0.808 | Paired Wilcoxon signed-rank test |
| CL | Gemini vs Copilot | 20 | -1.14 | -1.56--0.68 | -1.33 | -1.72--0.77 | -0.876 | -1.000--0.629 | 0.006 | Paired Wilcoxon signed-rank test |
| CL | Gemini vs DeepSeek | 20 | -0.44 | -1.16-0.21 | -0.06 | -1.04-0.52 | -0.190 | -0.619-0.333 | 0.571 | Paired Wilcoxon signed-rank test |
| CL | Gemini vs Doubao | 20 | 0.27 | -0.30-0.87 | 0.16 | -0.54-1.01 | 0.171 | -0.343-0.610 | 0.571 | Paired Wilcoxon signed-rank test |
| CL | Copilot vs DeepSeek | 20 | 0.70 | 0.12-1.29 | 0.79 | -0.31-1.87 | 0.524 | 0.067-0.867 | 0.105 | Paired Wilcoxon signed-rank test |
| CL | Copilot vs Doubao | 20 | 1.41 | 0.70-2.07 | 1.66 | 0.54-2.44 | 0.781 | 0.467-0.981 | 0.008 | Paired Wilcoxon signed-rank test |
| CL | DeepSeek vs Doubao | 20 | 0.71 | -0.18-1.66 | 0.54 | -0.73-1.92 | 0.300 | -0.219-0.724 | 0.353 | Paired Wilcoxon signed-rank test |
| FKGL | ChatGPT vs Gemini | 20 | -1.73 | -2.19--1.25 | -1.85 | -2.51--1.04 | -0.971 | -1.000--0.857 | 0.001 | Paired Wilcoxon signed-rank test |
| FKGL | ChatGPT vs Copilot | 20 | -1.40 | -2.08--0.72 | -1.26 | -2.79--0.40 | -0.762 | -0.971--0.419 | 0.005 | Paired Wilcoxon signed-rank test |
| FKGL | ChatGPT vs DeepSeek | 20 | -1.64 | -2.14--1.11 | -1.97 | -2.35--0.80 | -0.905 | -0.991--0.657 | 0.001 | Paired Wilcoxon signed-rank test |
| FKGL | ChatGPT vs Doubao | 20 | 0.08 | -0.49-0.69 | -0.26 | -0.60-0.70 | 0.024 | -0.500-0.505 | 0.940 | Paired Wilcoxon signed-rank test |
| FKGL | Gemini vs Copilot | 20 | 0.33 | -0.24-0.90 | 0.28 | -0.26-0.54 | 0.305 | -0.200-0.724 | 0.342 | Paired Wilcoxon signed-rank test |
| FKGL | Gemini vs DeepSeek | 20 | 0.09 | -0.58-0.75 | 0.40 | -0.25-0.81 | 0.181 | -0.314-0.676 | 0.612 | Paired Wilcoxon signed-rank test |
| FKGL | Gemini vs Doubao | 20 | 1.81 | 1.23-2.43 | 1.81 | 1.22-2.23 | 0.952 | 0.819-1.000 | 0.001 | Paired Wilcoxon signed-rank test |
| FKGL | Copilot vs DeepSeek | 20 | -0.24 | -0.96-0.47 | -0.01 | -1.26-0.50 | -0.158 | -0.648-0.379 | 0.622 | Paired Wilcoxon signed-rank test |
| FKGL | Copilot vs Doubao | 20 | 1.48 | 0.83-2.18 | 1.49 | 0.52-2.19 | 0.829 | 0.543-0.991 | 0.003 | Paired Wilcoxon signed-rank test |
| FKGL | DeepSeek vs Doubao | 20 | 1.72 | 0.88-2.56 | 1.86 | 0.40-2.35 | 0.800 | 0.476-0.971 | 0.004 | Paired Wilcoxon signed-rank test |
| GFI | ChatGPT vs Gemini | 20 | -0.79 | -1.34--0.23 | -0.65 | -1.50--0.45 | -0.610 | -0.919--0.200 | 0.054 | Paired Wilcoxon signed-rank test |
| GFI | ChatGPT vs Copilot | 20 | -0.59 | -1.13--0.07 | -0.75 | -1.00--0.10 | -0.553 | -0.937--0.073 | 0.073 | Paired Wilcoxon signed-rank test |
| GFI | ChatGPT vs DeepSeek | 20 | -1.00 | -1.68--0.29 | -1.25 | -2.00--0.50 | -0.595 | -0.914--0.181 | 0.054 | Paired Wilcoxon signed-rank test |
| GFI | ChatGPT vs Doubao | 20 | -0.00 | -0.66-0.64 | 0.25 | -0.95-0.95 | -0.014 | -0.495-0.495 | 0.970 | Paired Wilcoxon signed-rank test |
| GFI | Gemini vs Copilot | 20 | 0.20 | -0.34-0.75 | 0.30 | -0.85-1.20 | 0.200 | -0.333-0.676 | 0.570 | Paired Wilcoxon signed-rank test |
| GFI | Gemini vs DeepSeek | 20 | -0.21 | -0.83-0.41 | -0.20 | -0.45-0.45 | -0.168 | -0.652-0.368 | 0.592 | Paired Wilcoxon signed-rank test |
| GFI | Gemini vs Doubao | 20 | 0.78 | 0.22-1.33 | 0.75 | 0.15-1.25 | 0.591 | 0.152-0.909 | 0.054 | Paired Wilcoxon signed-rank test |
| GFI | Copilot vs DeepSeek | 20 | -0.41 | -0.91-0.06 | 0.00 | -1.20-0.30 | -0.357 | -0.779-0.210 | 0.273 | Paired Wilcoxon signed-rank test |
| GFI | Copilot vs Doubao | 20 | 0.59 | 0.05-1.13 | 0.20 | -0.05-1.65 | 0.479 | 0.016-0.857 | 0.117 | Paired Wilcoxon signed-rank test |
| GFI | DeepSeek vs Doubao | 20 | 1.00 | 0.33-1.68 | 1.35 | -0.45-1.95 | 0.648 | 0.248-0.905 | 0.054 | Paired Wilcoxon signed-rank test |
| SMOG | ChatGPT vs Gemini | 20 | -1.30 | -1.63--0.94 | -1.42 | -1.86--0.78 | -0.971 | -1.000--0.857 | <0.001 | Paired Wilcoxon signed-rank test |
| SMOG | ChatGPT vs Copilot | 20 | -0.87 | -1.31--0.43 | -0.66 | -1.51--0.30 | -0.781 | -1.000--0.438 | 0.004 | Paired Wilcoxon signed-rank test |
| SMOG | ChatGPT vs DeepSeek | 20 | -1.39 | -1.82--0.98 | -1.38 | -2.08--0.61 | -0.962 | -1.000--0.829 | <0.001 | Paired Wilcoxon signed-rank test |
| SMOG | ChatGPT vs Doubao | 20 | 0.38 | -0.01-0.77 | 0.36 | -0.20-0.99 | 0.432 | -0.086-0.817 | 0.136 | Paired Wilcoxon signed-rank test |
| SMOG | Gemini vs Copilot | 20 | 0.42 | -0.04-0.87 | 0.34 | -0.12-0.80 | 0.414 | -0.086-0.819 | 0.136 | Paired Wilcoxon signed-rank test |
| SMOG | Gemini vs DeepSeek | 20 | -0.09 | -0.59-0.39 | -0.04 | -0.56-0.47 | -0.062 | -0.533-0.457 | 0.823 | Paired Wilcoxon signed-rank test |
| SMOG | Gemini vs Doubao | 20 | 1.68 | 1.24-2.12 | 1.82 | 0.94-2.32 | 0.976 | 0.886-1.000 | <0.001 | Paired Wilcoxon signed-rank test |
| SMOG | Copilot vs DeepSeek | 20 | -0.52 | -1.04--0.01 | -0.41 | -1.29-0.32 | -0.386 | -0.790-0.114 | 0.150 | Paired Wilcoxon signed-rank test |
| SMOG | Copilot vs Doubao | 20 | 1.26 | 0.82-1.73 | 0.96 | 0.61-1.94 | 0.962 | 0.829-1.000 | <0.001 | Paired Wilcoxon signed-rank test |
| SMOG | DeepSeek vs Doubao | 20 | 1.77 | 1.22-2.35 | 1.93 | 0.70-2.60 | 0.971 | 0.857-1.000 | <0.001 | Paired Wilcoxon signed-rank test |
| FRES | ChatGPT vs Gemini | 20 | 3.00 | 0.35-5.70 | 2.00 | 0.00-6.50 | 0.536 | 0.033-0.912 | 0.110 | Paired Wilcoxon signed-rank test |
| FRES | ChatGPT vs Copilot | 20 | 4.90 | 1.55-7.95 | 5.50 | 1.50-9.50 | 0.643 | 0.219-0.971 | 0.041 | Paired Wilcoxon signed-rank test |
| FRES | ChatGPT vs DeepSeek | 20 | 6.65 | 3.05-10.05 | 8.00 | 4.00-11.00 | 0.737 | 0.348-0.988 | 0.041 | Paired Wilcoxon signed-rank test |
| FRES | ChatGPT vs Doubao | 20 | 0.85 | -2.85-4.35 | 1.50 | -2.50-5.00 | 0.195 | -0.314-0.676 | 0.455 | Paired Wilcoxon signed-rank test |
| FRES | Gemini vs Copilot | 20 | 1.90 | -0.55-4.30 | 2.50 | -1.00-6.00 | 0.368 | -0.152-0.779 | 0.233 | Paired Wilcoxon signed-rank test |
| FRES | Gemini vs DeepSeek | 20 | 3.65 | 0.45-7.10 | 2.50 | -1.00-6.00 | 0.491 | -0.015-0.862 | 0.117 | Paired Wilcoxon signed-rank test |
| FRES | Gemini vs Doubao | 20 | -2.15 | -5.45-0.60 | -1.00 | -4.00-2.50 | -0.252 | -0.686-0.281 | 0.367 | Paired Wilcoxon signed-rank test |
| FRES | Copilot vs DeepSeek | 20 | 1.75 | -0.90-4.60 | 1.00 | -3.00-7.50 | 0.286 | -0.238-0.695 | 0.338 | Paired Wilcoxon signed-rank test |
| FRES | Copilot vs Doubao | 20 | -4.05 | -7.30--1.00 | -3.50 | -7.00--2.00 | -0.562 | -0.914--0.124 | 0.071 | Paired Wilcoxon signed-rank test |
| FRES | DeepSeek vs Doubao | 20 | -5.80 | -9.70--2.00 | -6.50 | -10.00--0.50 | -0.678 | -0.947--0.250 | 0.041 | Paired Wilcoxon signed-rank test |

Note: Pairwise comparisons were performed using paired Wilcoxon signed-rank tests with Benjamini-Hochberg correction. Paired mean/median differences and rank-biserial correlations are reported with bootstrap 95% CIs.
